# The ancient cline of haplogroup K implies that the Neolithic transition in Europe was mainly demic

**DOI:** 10.1038/s41598-017-11629-8

**Published:** 2017-09-11

**Authors:** Neus Isern, Joaquim Fort, Víctor L. de Rioja

**Affiliations:** 1 Complex Systems Laboratory, Universitat de Girona, C/Maria Aurèlia Capmany 61, 17003 Girona, Catalonia Spain; 2 Catalan Institution for Research and Advanced Studies (ICREA), Passeig Lluís Companys 3, 08010 Barcelona, Catalonia Spain

## Abstract

Using a database with the mitochondrial DNA (mtDNA) of 513 Neolithic individuals, we quantify the space-time variation of the frequency of haplogroup K, previously proposed as a relevant Neolithic marker. We compare these data to simulations, based on a mathematical model in which a Neolithic population spreads from Syria to Anatolia and Europe, possibly interbreeding with Mesolithic individuals (who lack haplogroup K) and/or teaching farming to them. Both the data and the simulations show that the percentage of haplogroup K (%K) decreases with increasing distance from Syria and that, in each region, the %K tends to decrease with increasing time after the arrival of farming. Both the model and the data display a local minimum of the genetic cline, and for the same Neolithic regional culture (Sweden). Comparing the observed ancient cline of haplogroup K to the simulation results reveals that about 98% of farmers were not involved in interbreeding neither acculturation (cultural diffusion). Therefore, cultural diffusion involved only a tiny fraction (about 2%) of farmers and, in this sense, the most relevant process in the spread of the Neolithic in Europe was demic diffusion (i.e., the dispersal of farmers), as opposed to cultural diffusion (i.e., the incorporation of hunter-gatherers).

## Introduction

The Neolithic transition was a major transformation that introduced agricultural economics, radically changed the environment, and led to increased population densities and new forms of social organization^1, 2^. The Neolithic spread from the Near East across Europe, from about 8,000 yr Before the Common Era (BCE) until about 3,000 yr BCE^3^. A crucial question is whether the spread of the Neolithic was due to a dispersal of farming populations (demic diffusion), to the learning of agricultural techniques by European hunter-gatherers (cultural diffusion), or to a combination of both mechanisms. The latter possibility is suggested by the comparison of archaeological data to mathematical wave-of-advance models, which indicate that demic diffusion was more important than cultural diffusion^4, 5^. Genome-wide studies also indicate a crucial role for demic diffusion, with very little cultural diffusion at the beginning of the Neolithic^6, 7^. Notwithstanding the unquestionable importance of genome-wide studies, it is also of interest to analyze specific genetic markers, for two reasons. First, genome-wide studies cannot provide any quantitative explanation for the observed spatial cline of a single marker. And secondly, genome-wide studies cannot yield a quantitative estimate for the percentage of farmers involved in cultural diffusion. In order to understand both limitations of genome-wide studies, consider first one marker that has not been affected by drift neither selection. If there is admixture between the populations of incoming farmers and indigenous hunter-gatherers (HGs), and the latter originally lacked this marker, then it will dilute progressively, i.e. its frequency will decrease with increasing distance from the spatial region of origin of the Neolithic front. Second, consider again a maker unaffected by drift neither selection, but such that HGs initially had higher frequencies than farmers. Its frequency will not decrease but increase with distance from the Neolithic origin. Thirdly, consider a marker that increased its frequency after some location during the spread of the Neolithic front (due, e.g., to surfing or other drift effects). If HGs originally lacked this marker, its frequency will decrease (due to admixture) up to some distance, and increase for larger distances. Fourthly, if several drift and/or selective effects took place, the cline can have even more complicated shapes. Thus, clearly the frequencies of different genetic markers have different spatiotemporal dependencies, because they are due to different processes. For this reason, in order to estimate the percentage of farmers involved in cultural diffusion we should not to include many arbitrary markers (as in genome-wide studies). Instead, we should consider very specific markers that satisfy the following conditions: (1) the frequency decreases with increasing distance from the spatial origin of the Neolithic front; (2) HGs lack the marker considered before the arrival of the first farmers (otherwise we would need to know the precise space-time variation of the marker initial frequency in HGs); (3) selection and (4) drift (including surfing) effects can be neglected. This makes it possible to compare the data to demic-diffusion models neglecting drift, selection, etc. (as done below). In the present paper we analyze mitochondrial haplogroup K because, as we shall see, the observed data for this marker satisfy conditions (1) and (2). In contrast, other markers that have been found in Early Neolithic European sites (e.g., N1a, J, T and X) have not been found in the Near East^8^, so condition (1) does not hold. Condition (3) can be also reasonably assumed, because there are no data indicating the existence of any selective pressure on haplogroup K, and analysis of the Early Neolithic K haplotypes does not show signs of selection (Supplementary Text S1). It is also reasonable to assume that condition (4) holds, because we will show that a simulated cline (neglecting drift) is consistent with the observed one for haplogroup K.

A totally independent reason why genome-wide studies cannot determine quantitatively the percentage of farmers involved in cultural diffusion is that, e.g., Mathieson *et al*.^6^ assume only two source populations, namely an Anatolian Neolithic one and Western HGs, and use *f*_4_-statistics to estimate, e.g., a 93% of Anatolian Neolithic ancestry and a 7% of Western HG ancestry for Early Neolithic farmers in Germany. But this result of 93% is not the *percentage of farmers involved in cultural diffusion*. Instead, it is the Anatolian fraction (${\alpha }_{{\rm{1}}}=0{\rm{.93}}$) of genetic drift (defined as a variance of allele frequencies^9^) of the German population considered (assuming that its drift is a linear combination of the drifts of the two presumed source populations). But there is no mathematical theory relating the proportions of genetic drift (i.e., the coefficients *α*_1_, *α*_2_, …, *α*_N_ of the *f*_4_-value of a test population in terms of the *f*_4_-values of its *N* presumed source populations^6, 9^) to the percentage of farmers involved in cultural diffusion. Similarly, in admixture analysis the fractions of the genome contributed by a set of presumed source populations are estimated, but again there is no theory relating them to the percentage of farmers involved in cultural diffusion. For totally analogous reasons, these and other previous methods (*f*_4_-statistics, admixture, principal components, structure analysis, *D-*statistics, etc.) can provide valuable qualitative indications on whether demic or cultural diffusion dominated the Neolithic spread, but they cannot yield any quantitative value for the percentage of farmers involved in cultural diffusion. Incidentally, we note that many such methods (e.g., *f*_4_-statistics and admixture) assume a few source populations, whereas here we will consider the more realistic case of populations distributed continuously in space (and also include the effect of seas and mountains). If clinal patterns are not observed in analyses based on principal components, admixture, *f*_3_, *f*_4_, *D*-statistics, etc. (where, instead, early Neolithic individuals tend to cluster together, e.g. with modern Sardinians), the reason is simply that those analyses are based on many markers but, as explained above, the spatial distribution of each one can be due to other processes in addition cultural diffusion (surfing, other kinds of drift, selection, etc.).

In this article we shall estimate the percentage of early farmers involved in cultural diffusion from an ancient DNA (aDNA) marker. We will perform our analysis at the continental scale, because aDNA data are not yet numerous enough to consider specific geographic regions. As we shall see, however, there are already sufficient data to obtain some first estimates of general trends. We consider mitochondrial DNA (mtDNA), because nuclear data are known for a substantially smaller number of ancient individuals. Mitochondrial DNA is inherited from the mother, thus its study will be related to the spread of maternal lineages. As all genetic sequences, mtDNA can be inherited with mutations, but similar sequences (haplotypes) with a common ancestor are usually grouped into haplogroups. Since the aDNA data are still limited in number, we perform our analysis below at the haplogroup level, grouping together the different haplotypes and subclades from each lineage (in Supplementary Text S1 we include analyses at the haplotype level, and they reinforce our conclusions). The mtDNA of European hunter-gatherers is composed mainly of U lineages (U, U4, U5, and U8), which are absent in early Neolithic populations^10–12^. Conversely, haplogroups N1a, T2, K, J, HV, V, W, and X have been proposed as potential Neolithic markers because they have been found in farmers of the Linearbandkeramic (LBK) culture, an early Neolithic culture in Central Europe, and are almost absent in neighboring hunter-gatherer samples^10, 13^. Haplogroup K has been identified in only three hunter-gatherers dated before the arrival of farming (two in Greece^14^ and one in Georgia^12^), but their subclades have not been found so far in any Neolithic farmer (see Supplementary Text S2 for a detailed discussion of the very few exceptions of Mesolithic individuals displaying K haplotypes). Thus haplogroup K was virtually absent in Europe before the arrival of farming, and condition (2) above is satisfied. On the other hand, as we shall see below, haplogroup K displays a cline of decreasing frequency with increasing distance from the spatial origin of the Neolithic expansion. Thus haplogroup K also satisfies condition (1) above, in contrast to other potential Neolithic markers (N1a, T2, J, HV, V, W, and X).

## Results and Discussion

**Figure 1:**
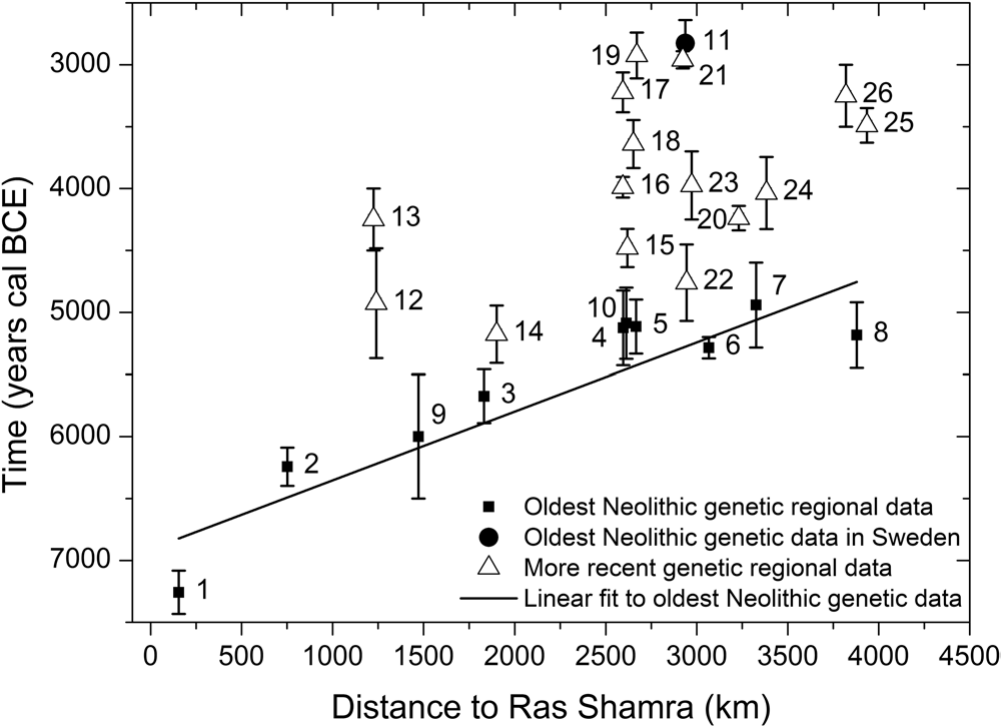
Dates versus great-circle distances from Ras Shamra (Syria) for 26 regional cultures with ancient mtDNA data. Squares correspond to the oldest regional Neolithic cultures, namely 1 Syria PPNB (15 individuals), 2 Anatolia (28 individuals), 3 Hungary-Croatia Starčevo (44 individuals), 4 Eastern Germany LBK (36 individuals), 5 Western Germany LBK (56 individuals), 6 Northeastern Spain Cardial (15 individuals), 7 Spain Navarre (36 individuals), 8 Portugal coastal Early Neolithic (10 individuals), 9 Romania Starčevo (5 individuals) and 10 Southern Germany LBK (4 individuals). The circle stands for 11 Sweden (9 individuals), which is substantially delayed due to the slowdown of the Neolithic front in northern Europe. Triangles correspond to more recent regional cultures, namely 12 Romania Middle Neolithic (29 individuals), 13 Romania Late-Middle Neolithic (9 individuals), 14 Hungary LBK (45 individuals), 15 Eastern Germany RSC (10 individuals), 16 Eastern Germany SCG/BAC (38 individuals), 17 Eastern Germany SMC (30 individuals), 18 Western Germany BAC (14 individuals), 19 Western Germany BEC (17 individuals), 20 Western France Prissé (3 individuals), 21 South-Eastern France Treilles (29 individuals), 22 Catalonia Epicardial (7 individuals), 23 Catalonia Late Epicardial (3 individuals), 24 Spain Basque country (7 individuals), 25 Portugal coastal Late Neolithic (3 individuals) and 26 Portugal inland Late Neolithic (7 individuals). The straight line is the regression fit to the 10 oldest regional data (squares). The symbols and extremes of each error bar give the averages of the mean, maximum and minimum calibrated dates, computed over all individuals with known mtDNA in the corresponding regional culture (Supplementary Data S1).

In order to study the existence of a genetic cline for haplogroup K in early Neolithic populations and subsequently compare it to our simulations, we have gathered all mtDNA information of Early and Middle Neolithic individuals reported in the literature, and we have grouped the data into regional cultures according to their location, date and reported culture (Supplementary Data S1). The Neolithic expansion in Europe begun in the Near East, and for this reason we have used the oldest pre-pottery Neolithic B (PPNB) date from Syria^3^, Ras Shamra, as a geographic reference for the origin of the spread. In Fig. 1 we represent, for each regional culture, the average date of its individuals whose mtDNA haplogroup has been determined against the distance from their average location to Ras Shamra. Figure 1 includes all regional cultures dated between the Early and the Middle Neolithic, such that the mtDNA haplogroup of more than two individuals is known (e.g., Greece could not be included; see Supplementary Text S3). The Southern Levant is not included for reasons explained in Supplementary Text S3. For each regional culture, the number of individuals whose mtDNA haplogroup has been determined is given in the caption to Fig. 1 (Supplementary Data S2–S3). We distinguish 3 different groups of regional cultures in Fig. 1. The first group is composed by the 10 *oldest* Neolithic regional cultures (from Syria to western and northern Europe) for which there are genetic data (squares in Fig. 1). The second group (triangles in Fig. 1) corresponds to 15 regional cultures that have *younger* dates than the oldest ones (squares) and that are located at similar distances from Syria (i.e., broadly in the same area). Thus, the triangles in Fig. 1 are not representative of the earliest local Neolithic cultures. Finally, the circle in Fig. 1 corresponds to Sweden. Its date and location are those of the earliest Neolithic individuals in Sweden whose mtDNA is known. It would be thus legitimate to consider this data point (circle in Fig. 1) simply as one of the oldest regional cultures (squares), and we will actually include it into our calculations below. But the date for Sweden is substantially delayed relative to other cultures located at similar distances (Fig. 1), so it will be useful to identify Sweden with a symbol (circle) different than the other oldest regional cultures (squares).

### Understanding the observed variations in the percentage of haplogroup K (%K)

**Figure 2:**
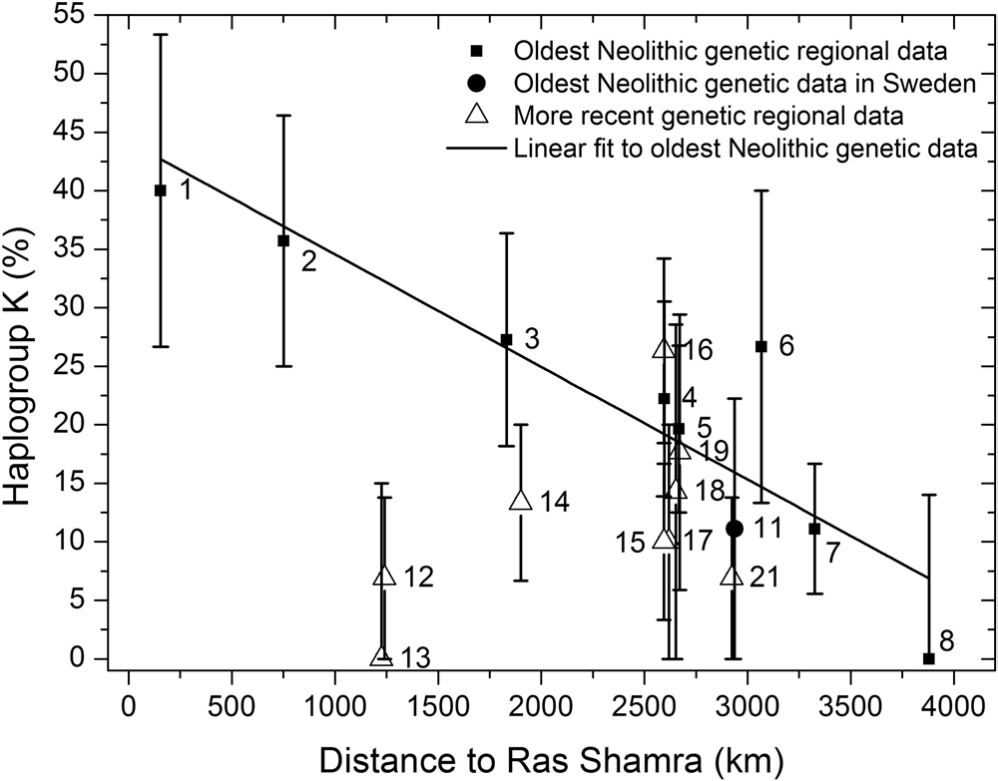
Observed percentage of mtDNA haplogroup K as a function of the great-circle distance from Ras Shamra (Syria). Each number denotes the same culture as in Fig. 1 (regions with fewer than 8 individuals have been ignored to avoid very large error bars). The straight line is the regression fit to the 10 oldest regional data (squares) and the oldest data in Sweden (circle). Error bars display 80% CL intervals (see Materials and Methods, Statistical analysis and Supplementary Text S10).

It is important to keep in mind that the *oldest* regional cultures displayed in Fig. 1 do not correspond to the oldest archaeological dates known for each Neolithic regional culture, but only to the oldest Neolithic individuals whose mtDNA haplogroup has been determined. In spite of this, those dates (squares in Fig. 1) show a highly linear dependence on distance (correlation coefficient $R=0.93$), as predicted for the oldest dates by wave-of-advance models^4^. In Fig. 2 we plot the %K as function of distance from Ras Shamra (Syria) for all the regional cultures in Fig. 1 that include at least 8 individuals (regions with fewer individuals have been ignored to avoid very large error bars). Because the total number of individuals per region is still small in many regions, in our analysis below we take into account the whole 80% confidence-level (80% CL) range, represented as error bars, rather than only mean values. Labels and symbols in Fig. 2 are the same as in Fig. 1. For the oldest Neolithic cultures, there is no theoretical reason to expect a linear dependency of the %K on distance (in other words, we should not expect a high value of *R* for the regression to the squares and circle in Fig. 2). However, the slope of this regression in Fig. 2 is highly significantly different from zero ($P=0.001$), and this gives statistical support to the existence of a genetic cline (similarly, low values of *P* also yield statistical support to the existence of phonemic clines^15, 16^). Additional support to the existence of a cline is obtained from an interpolation map and the analysis of the %K data by means of a Moran’s I correlogram, included in Supplementary Text S4.

As explained in the Introduction, it has been proposed that haplogroup K spread across Europe from the Near East with the Neolithic front^8, 10, 17–19^. We shall call this proposal the wave-of-advance model of haplogroup K. The analysis of the Early Neolithic haplotypes in our database also yields support to the assumption that the population with haplogroup K underwent a recent process of demographic and geographic expansion (Supplementary Text S1). Obviously demic diffusion, on its own, cannot explain the spatiotemporal distribution of haplogroup K (as displayed in Fig. 2), because purely demic diffusion predicts a uniform distribution (see (i) below and Sec. Demic versus cultural diffusion). Thus we ask whether cultural (in addition to demic) diffusion is a viable explanation. If HGs lacked haplogroup K (as justified by genetic data in the Introduction and Supplementary Text S2), and other effects (selection, drift, mutation, etc.) can be neglected, a demic-cultural model makes two testable predictions. (i) For the earliest Neolithic cultures, we should observe a decrease in the percentage of farmers with haplogroup K with increasing distance from the Near East (because interbreeding of pioneer farmers with local hunter-gatherers, and/or acculturation of the latter during the front propagation, will diminish the %K). This prediction is clearly observed in Fig. 2, because the earliest regional Neolithic cultures (squares and circle) show a clear decrease of the %K with increasing distance from Syria. (ii) For each region, this model also predicts that the earliest Neolithic regional culture will have a higher percentage of farmers with haplogroup K than later cultures (due to interbreeding and acculturation subsequent to the arrival of the Neolithic wave of advance). Prediction (ii) is also observed in Fig. 2, because of the 9 European cultures that do not correspond to the earliest Neolithic (triangles), only 1 (culture 16) has a larger %K than the expected regional maximum (the latter is given by the linear fit to the earliest regional Neolithic cultures in Fig. 2), and even culture 16 may be lower than the expected maximum, if the error bar is taken into account. However, we must caution that prediction (ii) refers to populations dated substantially later than the spread of the Neolithic front and it is therefore affected by population movements and other processes subsequent to the spread of the Neolithic. Thus, it is not reasonable to try to explain *quantitatively* prediction (ii) with a simulation model of the spread of the Neolithic. For this reason, although the model satisfies *qualitatively* both predictions, in the rest of this paper we shall be mainly concerned with prediction (i).

### Ancient cline of haplogroup K

**Figure 3:**
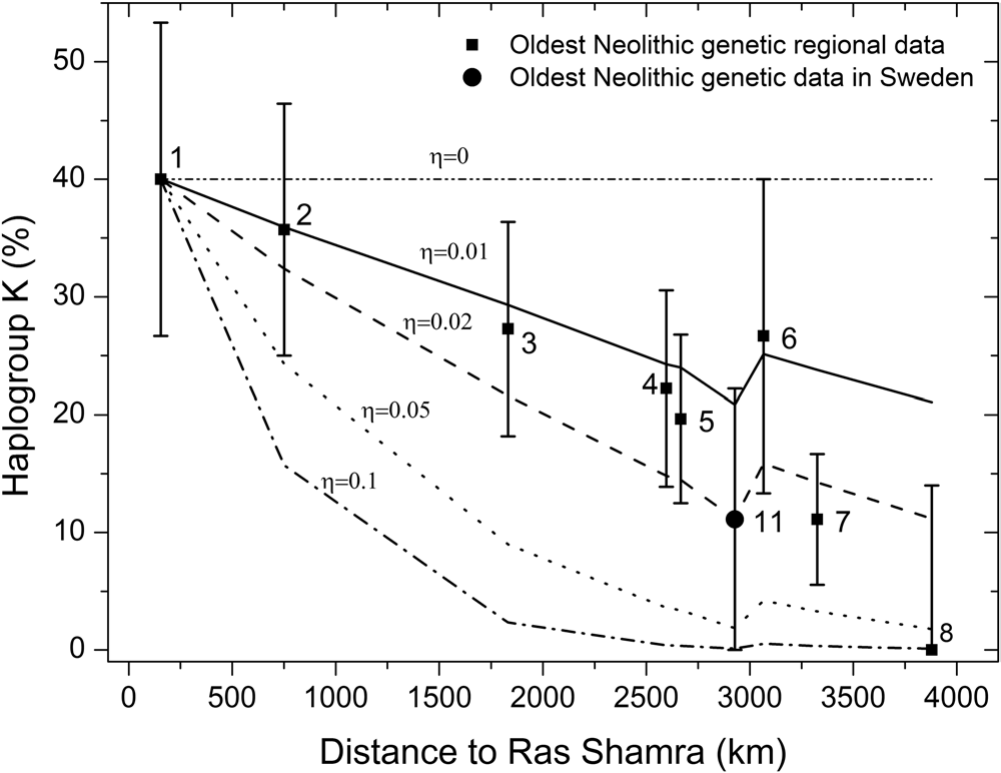
Observed and simulated percentage of mtDNA haplogroup K as a function of the great-circle distance from Syria. The data are shown with the same error bars as in Fig. 2, but only for the oldest regional cultures. The lines are the results of the mathematical simulation for several values of the cultural diffusion intensity *η*. The lines have been plotted by joining the simulation results for each of the 9 regional cultures, obtained at the average location and date of the individuals whose mtDNA haplogroup has been determined for each regional culture (Supplementary Data S1). Therefore, the simulation result for each region has been obtained at its average date (Fig. 1 and Supplementary Data S1). Numerical labels denote the same cultures as in Figs 1–2.

Figure 3 shows (lines) the clines obtained from our wave-of-advance simulations of the Neolithic and haplogroup K spread (see Materials and Methods and Supplementary Texts S5–S9), alongside the observed genetic data for the earliest regional Neolithic cultures (squares and circle) already depicted in Fig. 2. In Fig. 3 we have imposed the initial genetic conditions that all simulations predict the observed %K for Syria (square labelled 1; see more details on the implementation of the initial conditions in Materials and Methods and Supplementary Text S7). The simulated clines have been computed at the same 9 locations and dates as the genetic data (so the lines simply join the 9 data points), and for several values of the cultural diffusion intensity *η*.

We first observe that, similarly to the behavior of the data (symbols in Fig. 3) and in agreement with prediction (i) formulated above, when considering cultural diffusion (*η* ≠ 0), the %K from the simulations (lines) tends to decrease with increasing distance from the Near East. This behavior was to be expected, because more distance from the origin (Ras Shamra, Syria) implies more time for the farming populations to interact (via interbreeding or/and acculturation) with hunter-gatherers (who lack haplogroup K). However, we note that both the simulations and the data display a local minimum at region 11 (Sweden). This is due to the fact that, according to archaeology^3^ and ancient genetics^20, 21^, the spread of the Neolithic in Europe occurred following two main routes: one along the Mediterranean coast (corresponding to the Impressa and Cardial traditions) and the other through the Balkans and the Central European plains (corresponding to the Starčevo and LBK cultures). To see how this explains the minimum in Fig. 3, consider first the Neolithic front propagating along the Mediterranean coast. In this case, population dispersal is driven by jumps (maritime migrations) of about 150 km per generation (as shown in Materials and Methods and Supplementary Text S6, and in agreement with previous simulation results^3^). Conversely, the Neolithic front propagating inland is driven by jumps of about 50 km per generation (Materials and Methods). Therefore, in order for the Neolithic front to travel a given distance, a *coastal* propagation obviously implies fewer jumps, i.e., fewer generations, and therefore less time for interbreeding with hunter-gatherers (and/or acculturation of the latter) than an *inland* propagation. Thus a *coastal* route will lead, at a given distance, to a lower decrease of the %K than an *inland* route. This is why the Mediterranean route leads, in region 6 (NE Spain) in Fig. 3, to higher values of the %K than the central-northern European route in region 11 (Sweden), in spite of the fact that the former is further away from Syria than the latter. This explains the minimum in the simulation curves (and in the observed data) in Fig. 3 (see Supplementary Text S8 for a more detailed discussion, and Fig. S15 for a plot of the simulated clines along both routes).

### Demic versus cultural diffusion

What does the observed cline of haplogroup K for Early Neolithic cultures (error bars in Fig. 3) imply about the importance of cultural diffusion in the spread of the Neolithic? First, let us examine how the intensity *η* of cultural diffusion is related to the steepness of the genetic cline. Note that, in the absence of cultural diffusion (i.e., without interbreeding neither acculturation), the %K at all farming populations would remain approximately constant at the value observed for the original (PPNB) population in Syria (assuming that drift and other processes do not have a strong effect). Thus, in a purely demic model (*η* = 0), such a cline would not be observed. Accordingly, the simulation for *η* = 0 leads to a uniform distribution in Fig. 3. We also expect that the stronger the intensity of cultural diffusion, the more important the decrease in the frequency of haplogroup K, and the steeper its geographic cline. This intuitive expectation agrees with the simulation results in Fig. 3, where for any given distance from the origin, a higher value of the cultural transmission intensity *η* yields a lower %K.

By comparing the data (symbols) to the demic-cultural space-time simulations (lines), we observe that Fig. 3 implies that the intensity of cultural diffusion was $\eta \approx 0.02$ (because higher or lower values of *η* lead to lines that are not within all of the error bars obtained from the aDNA data). The maximum possible value of this parameter is $\eta =1$ ^22^ (see the text below equation (2)). Therefore, although the observed cline cannot be explained without cultural diffusion (*η* = 0, horizontal line in Fig. 3), such a low value ($\eta \approx 0.02$) implies that cultural diffusion was remarkably weak. Indeed, the cultural diffusion intensity *η* can be interpreted as the proportion of pioneering farmers that mate a hunter-gatherer^22^ or, alternatively, that teach agriculture to a hunter-gatherer^4^ (Supplementary Text S9). Thus, our result that $\eta \approx 0.02$ (Fig. 3) implies that cultural diffusion involved only a tiny fraction (about 2%) of farmers and, in this sense, the most relevant process in the Neolithic spread in Europe was demic diffusion. Modifying the initial conditions so that the whole 80% CL for Syria is considered refines this estimate of the percentage of farmers involved in cultural transmission to the range 2% ± 1% (Supplementary Text S7). The primacy of demic diffusion has been noted in genome-wide studies (see, e.g., previous work by Mathieson *et al*.^6^), but those studies could not quantify the percentage of farmers involved in cultural diffusion (see our Introduction). In contrast, we quantify that about 98% of farmers did not take part in cultural diffusion.

Our main result, namely that a very small amount of cultural transmission is enough to produce a continent-wide genetic cline, agrees with previous simulations^23–25^, which however did not use the equations of cultural transmission theory recently derived^4, 22^ (see equations (1)-(3) and Supplementary Texts S5, S9 and S11) nor could compare to aDNA data (which were then also unavailable). Therefore, in none of those previous studies was it possible to estimate quantitatively the percentage of farmers involved in cultural diffusion.

## Conclusions

In this paper we have analyzed the genetic implications of a mathematical model that combines demic dispersal, population growth, and cultural transmission theory. Using anthropologically realistic assumptions and parameter values, we have performed, to the best of our knowledge, the first qualitative and quantitative comparison of a mathematical model to an observed Neolithic genetic cline. Although the ancient genetic data currently available are still limited, especially those corresponding to the Early Neolithic, they cover a wide enough area (see Supplementary Text S4, Fig. S7) to allow us to analyze the geographical cline of genetic markers at the continental level, even if regional variations cannot be detected. In addition, the data are numerous enough so that we can observe a cline, and reach conclusions valid at least at the 80% CL (error bars in Fig. 3 and in Supplementary Text S7, Figs S12–S14). A Moran’s I correlogram confirms the existence of the cline (Supplementary Text S4, Fig. S8). We have focused our attention on haplogroup K, mainly because it is virtually absent in hunter-gatherer populations and its frequency has a maximum in the Near East (specifically in Syria). Both points make it possible to attempt a description based on a simple mathematical model.

Qualitatively, the model predictions agree with the data in two ways: (i) both the data and the simulations show that the %K tends to decrease with increasing distance from Syria (Fig. 3); (ii) for each region, the %K tends to decrease with increasing time after the arrival of farming (Fig. 2).

Quantitatively, comparison between the model and the data shows that: (i) both the model and the data display a local minimum of the genetic cline, and for the same regional culture (Sweden, i.e. symbol 11 in Fig. 3); (ii) the ancient cline of haplogroup K can be explained if about 98% of farmers were not involved in cultural diffusion. However, we stress that the observed cline cannot be understood assuming that 100% of farmers were not involved in cultural diffusion. Thus, the observed cline implies that some farmers took part in cultural transmission (either by interbreeding or by teaching agriculture to hunter-gatherers). But only a tiny fraction (about 2%) of farmers were involved in cultural diffusion. In this sense, the most relevant process in the expansion of Neolithic culture in Europe was demic diffusion, i.e. the reproduction and dispersal of farmers, as opposed to the incorporation of hunter-gatherers (cultural diffusion).

Recently, the conclusion that the spread of the Neolithic in Europe was driven mainly by demic diffusion has been also obtained from comparing non-genetic, demic-cultural models to the spread rate of the Neolithic front, as estimated from archaeological data^4^. However, using only archaeological data has severe limitations. The reason is the following. Archaeological data make it possible to estimate the spread rate of the Neolithic wave of advance, and this can be compared to the results of the mathematical model. But the dependence of the spread rate on the intensity of cultural transmission is weak^4, 22^ and, for this reason, the spread rate can be used only to estimate an upper bound for the intensity of cultural transmission (namely $0 < C < 2.5$ ^4^, equivalent to $0 < \eta  < 2.5$ here, see Supplementary Text S9). In contrast, here we have shown that genetic data make it possible to know a function that depends strongly on the intensity *η* of cultural transmission (Fig. 3), namely the percentage of the considered haplogroup as a function of distance (i.e., the genetic cline shown in Fig. 3). This strong dependency has made possible a much more precise estimation of the percentage of farmers involved in cultural diffusion, namely $\eta =0.02$ (Fig. 3), i.e. about 2%. This shows the tremendous potential of combining genetics, archaeology and mathematical modelling. On the other hand, the high number of archaeological data has allowed the identification of regional variations^5^, something that is still not possible on the basis of ancient genetic data.

Our findings agree with genome-wide results, in the sense that demic diffusion was the main driver of the Neolithic spread in Europe (see, e.g. the results by Mathieson *et al*.^6^). However, genome-wide studies cannot estimate the percentage of farmers involved in cultural diffusion (see our Introduction). In contrast, our methodology yields the first quantitative estimation for this percentage (about 2%). This is possible because, in contrast to genome-wide studies, our approach has two crucial features: first, we compare to cultural-demic wave-of-advance mathematical models; second, we use a marker that shows decreasing frequency with increasing distance from the Near East. This estimate arises from comparing our model to the data at the 80% CL, leading to a confidence interval for the importance of cultural diffusion of 2% ± 1%. Of course, if additional such markers are identified in future work, they will yield more precise results and will also allow the study of regional variabilities. Thus the present paper is a first step, which also provides a plausible explanation for the observed cline of haplogroup K at a continental scale. We stress that such an explanation cannot be provided by genome-wide studies. For simplicity, our models assume the same dispersal behavior for males and females. If future studies detect ancient clines of decreasing frequency for additional genetic markers, and they consistently show differences between maternal and paternal markers, they could be used to infer different dispersal behaviors for females and males, using trivial extensions of our models.

Ancient DNA data indicate that cultural diffusion was more important in some specific regions, such as Scandinavia^26^ or the Paris Basin^27^. Thus, it has been recently suggested that the effect of cultural diffusion increased as farmers migrated farther west in Europe^27^. This suggestion agrees nicely with: (i) our simulated clines (lines in Fig. 3); (ii) the observed cline of haplogroup K (symbols in Fig. 3); and (iii) the intuitive expectation that longer distances from the spatial origin of the Neolithic imply more time for interbreeding and/or acculturation and, therefore, a stronger effect of cultural diffusion.

## Materials and Methods

### Archaeological and genetic data

We gathered a database of all individuals from farming cultures dated between 8,000 and 3,000 calibrated years BCE for which the mtDNA haplogroup have been reported in the literature. For all 513 individuals in the database, we report the haplogroup, date, latitude, longitude, bibliographical references and additional data (Supplementary Data S1). We grouped them into regional cultures according to their geographical and cultural closeness (*e.g*., Syria PPNB, Anatolia, Hungary-Croatia Starčevo, Hungary LBK, etc.). The data from Syria are from PPNB sites, which makes them especially relevant because PPNB/C are the Near-Eastern Neolithic cultures that later spread into Europe^3^. We selected for further analysis the 26 regional cultures with more than two individuals (comprising 508 individuals), and discarded the others (see Supplementary Text S3 for a discussion on Neolithic individuals not included in the analysis). For each of the 26 selected regional cultures, we calculated the percentage of individuals with K haplotypes (Supplementary Data S2–S3), the average date of its individuals, and the average great-circle distance of its individuals to the site of Ras Shamra (Supplementary Data S3). This is the oldest PPNB Syrian site used in previous simulations studies^3^, and we therefore use it as a reasonable geographic reference from the origin of the Neolithic range expansion in our simulations (see below).

### Statistical analysis

For each of the 26 regional cultures, we estimated the error intervals of its average date and %K. The time error bar (Fig. 1) was estimated  by averaging the reported maximum and minimum dates for all individuals in the considered regional culture whose mtDNA is known. The error bar for the %K (Figs 2–3) was estimated by the bootstrap method, computing the 80% CL interval of 10,000 replicates, except for the two regions where none of the sampled individuals have haplogroup K (‘Portugal coastal Early Neolithic’ and ‘Romania Late-Middle Neolithic’). Then the bootstrap method cannot be applied directly (because the error would be exactly zero, which is not reasonable), and thus we applied a different statistical method, explained in detail in Supplementary Text S10. We have established the existence of the cline in 3 ways: linear regression (Fig. 2), interpolation map and Moran’s I correlogram (Supplementary Text S4).

### Analysis of K haplotypes

We have applied several statistical and phylogenetic analysis to the K haplotypes found in the 9 Early Neolithic regional cultures: we have computed Tajima’s *D* and Fu’s *F*_*s*_ neutrality tests; analyzed the geographical variation in the haplotype diversity, mismatch distributions, and first principal component; correlated genetic and geographical distances through Mantel test; performed network analysis; and constructed a Bayesian Skyline Plot (Supplementary Text S1). The obtained results show clear signs of a recent demographic and spatial expansion, in agreement with our assumption that haplogroup K spread with the Neolithic wave. These analyses have also shown as that, in principle, the regions displaying high values of %K are not the result of sampling individuals from a single family (see Supplementary Text S1, sec. 2) Haplotype diversity).

### Space-time genetic simulations

We use a rectangular grid of square cells that covers the European continent, the Near East and part of Asia and Africa, with each cell classified as inland, coast, mountain or sea^3^. We use cells of 50 km × 50 km, since 50 km is the value corresponding to the mobility per generation according to ethnographic data of preindustrial populations^28^. At each cell we can have individuals of three populations: farmers who *have* haplogroup K, *P*_*N*_; farmers who do *not have* haplogroup K, *P*_*X*_; and hunter-gatherers, *P*_*HG*_ (no hunter-gatherer has haplogroup K). Each population would in principle include several different haplotypes, but since we are not interested in the evolution of any individual haplotypes, for simplicity the model used in the main paper does not consider any lower level subgroups. Below we describe the most important processes of the model, but we include a more detailed description in Supplementary Text S5.

#### Initial conditions

We applied the initial condition that at 8,233 yr BCE, the date of Ras Shamra (the oldest PPNB site in Syria from previous work^3^), all of the grid was empty of farmers except the cell that contains this site. In this cell, we set at 8,233 yr BCE the hunter-gatherer population density to zero, and the farmer population density to its saturation value (${P}_{F\max }=3,200\,\,\mathrm{individuals}/\mathrm{cell}$, from ethnographic data^3, 25^). The PPNB Syrian archaeological and genetic data have different times and locations (the archaeological data is dated at 8,233 yr BCE and the genetic data at 7,258 yr BCE). For this reason, we have to set the %K at the cell containing Ras Shamra by trial and error so that the simulation yields the adequate value of the %K at the time and location of the genetic data in Syria (see details in Supplementary Text S7). In all grid cells (except for the initial one), the hunter-gatherer population is initially set at its saturation value (${P}_{HG\max }=160\,\mathrm{individuals}/\mathrm{cell}$, from ethnographic data^25^), assuming that none of them has haplogroup K (see the Introduction and Supplementary Text S2).

Defining a generation as the mean age of the parents at the time one of their offspring is born (not necessarily the first), in simulations we use the mean value $T=32\,{\rm{yr}}$ obtained from ethnographic data^29^. Let *t* stand for the number of generations elapsed since the beginning of the simulation (8,233 yr BCE). For $t=1,\,2,\,\mathrm{3...}$ we apply the following cycle of 3 steps (changing their order would yield the same results):

1. ***Dispersal***. At each cell, we update the values of ${P}_{N}$ and ${P}_{X}$ by computing how many farmers of both kinds arrive at the cell from other cells. We do this, as in previous work^3, 22, 28^, with a simple model in which, for each cell, a fraction ${p}_{e}$ (which is called the persistence in demography) of the population of farmers (independently of their genes) stays at the cell, and a fraction $(1-{p}_{e})$ relocates to the four nearest neighbor cells, each receiving a fraction $(1-{p}_{e})/4$. We use the mean value ${p}_{e}=0.38$ obtained from ethnographic data^28^. We expect that including a set of distances and probabilities would lead to similar results^4^. If one or more of the nearest neighbors are mountain cells, they cannot receive population and each of the remaining neighbors receives a higher fraction. If one or more neighbors are sea cells, the corresponding fraction of the population (that would move there) travels by sea, and is equally distributed among coast cells that can be reached by sea in straight lines of up to 150 km (this is the adequate distance to obtain agreement with archaeological data, as seen in Supplementary Text S6 and in previous work^3^). We do not update the number of HGs in each cell due to their dispersal, because exchange of HGs between saturated cells has no effect (since all HGs lack haplogroup K) and we assume that they do not disperse appreciably into cells in which their number has been lowered due to cultural transmission (see step 2 below).
2. ***Cultural transmission***. This is the only step that was not included in our previous non-genetic simulations on a real map of Europe^3^, because they considered only purely demic models. There are 3 modes of cultural transmission^30^. Vertical transmission is due to interbreeding (i.e., cross-matings between farmers and HGs). Horizontal (oblique) transmission is due to learning of agriculture by HGs from farmers of the same (the previous) generation. The latter two modes can be combined in a single mathematical model, namely horizontal/oblique transmission^4^. Here we shall consider only vertical transmission for simplicity, but we would reach the same conclusions if we considered, instead, any combination of vertical and horizontal/oblique transmission (Supplementary Text S9).
  After dispersal, in each cell there is a population of ${P}_{HG}$ hunter-gatherers and ${P}_{N}+{P}_{X}$ farmers. To determine the population numbers of the new generation, we have to compute the matings that take place between and within those 3 population groups, and then apply the reproduction step. We assume that children of cross matings between farmers and HGs are farmers, in agreement with ethnographic observations^31, 32^. The number of cross matings between HGs and each group of farmers is^22^
  1 $$couples\,HN=\eta \frac{{P}_{HG}\cdot {P}_{N}}{{P}_{HG}+{P}_{N}+{P}_{X}},$$
  2 $$couples\,HX=\eta \frac{{P}_{HG}\cdot {P}_{X}}{{P}_{HG}+{P}_{N}+{P}_{X}},$$
  where ${P}_{HG}+{P}_{N}+{P}_{X}$ is the total population present at the cell, and parameter *η* is the intensity of interbreeding^22^. The case *η* = 1 corresponds to random mating. The case *η* > 1 corresponds to more cross matings than under random mating^22^, which is not realistic for farmers and HGs according to ethnographic data^32, 33^ (moreover, *η* > 1 can lead to negative population numbers^22^). Therefore, in practice $0\le \eta \le 1$.
  From equations (1)-(2) it is very easy to find the number of individuals $P{\text{'}}_{HG}$, $P{\text{'}}_{N}$, and $P{\text{'}}_{X}$ who do not take part in HN neither NX matings. We can use them to compute the number of matings between *farmer* individuals of different genetic groups (i.e., between populations $P{\text{'}}_{N}$ and $P{\text{'}}_{X}$) by using again vertical cultural transmission theory, and taking into account we have no reason to assume that farmers of a genetic group (i.e., with or without haplogroup K) will have a preference for (neither against) mating with farmers of the same genetic group. Thus we apply random mating (*η* = 1)^22^ for matings between farmers,
  3 $$couples\,NX=\frac{{P}_{N}^{{}^{{\rm{^{\prime} }}}}\cdot {P}_{X}^{{}^{{\rm{^{\prime} }}}}}{{P}_{N}^{{}^{{\rm{^{\prime} }}}}+{P}_{X}^{{}^{{\rm{^{\prime} }}}}},$$
3. ***Reproduction***. We apply the following rules. (i) Each couple will have $2{R}_{0,i}$ children, because ${R}_{0,i}$ (the net fecundity) is computed per parent and there are two parents per mating ($i=F,HG$). Ethnographic data indicate that the children of cross matings with one HG parent are farmers^31, 32^, thus we use ${R}_{0,HG}$ for HH matings and ${R}_{0,F}$ for HN, HX, NN, XX and NX matings. If the number of individuals computed for some population group, cell, and time step is larger than its corresponding maximum (${P}_{F\max }$ or ${P}_{HG\max }$), then we set it to the corresponding maximum value (Supplementary Text S5, sec. 3. Reproduc﻿tion). We expect that a logistic model would yield similar results. In our simulations we use, from ethnographic data, ${R}_{0,F}=2.45$ ^34^, indicating that after a generation, the size of the new population is 2.45 times the size of the parent population. We assume that ${R}_{0,HG}=1$, i.e. that the HG populations have reached a stationary state and they do not grow in number (not even after some HGs mate into the farming community, because converted HGs will still need part of the cell space after they become farmers); we do not expect our conclusions to change for other reasonable values of ${R}_{0,HG}$. (ii) For each kind of mixed genetic mating (HN and NX), in our simplest model we assume that the mother belongs to *P*_*N*_ in 50% of the matings, whose children will also carry haplogroup K since mtDNA is inherited from the mother (i.e., a 50% of the total offspring of mixed genetic matings will belong to *P*_*N*_). A more complicated model, assuming that mothers in HN and HX matings are always HGs (which is closer to ethnographic observations^32^) yields very similar results (Supplementary Text S11).

All the steps in the model are computed using real values for the population numbers. If we used a stochastic procedure to approximate them to integer values (at every cell, iteration, and process step), we expect that in average we would obtain the same results. We run our simulation program for 200 iterations (generations of 32 yr) for each set of parameter values, so that it covers the time from the start of the spread (Syria, 8,233 cal yr BCE) until the latest genetic data in the database (Sweden, 2,825 cal yr BCE; Supplementary Data S3). At each iteration we compute the number of HG, N and X individuals at each cell and record the latter two, so that we can compute the simulated %K (namely, $\tfrac{{P}_{N}}{({P}_{N}+{P}_{X})}\cdot 100$) and compare it to the observed one from the reported mtDNA data at each regional culture and its average date (Supplementary Data S3). This is done in Fig. 3.

## Electronic supplementary material

Supplementary Texts S1-S14

Supplementary Data S1-S7

Program S1

Program S2

Program S3

Program S4

Program S5
