# Supplementary material for: The ancient cline of haplogroup K implies that the Neolithic transition in Europe was mainly demic
Source: Sci Rep. 2017 Sep 11;7:11229. doi: 10.1038/s41598-017-11629-8 (PMC5594011; doi:10.1038/s41598-017-11629-8)
Supplement: Supplementary file 1 — Supplementary Texts S1-S14 [file 41598_2017_11629_MOESM1_ESM.pdf]

Supplementary Information Texts S1-S14

to Isern, Fort & de Rioja*, The ancient cline of haplogroup K implies that the Neolithic transition in Europe was mainly demic*

Text S1. Analysis of K haplotypes. Signs of spatial expansion 2
Text S2. Mesolithic samples with haplogroup K 11
Text S3. Neolithic individuals not included in the study 12
Text S4. Geographic cline of haplogroup K 12
Text S5. Mathematical details of the computational model 15
Text S6. Estimation of the characteristic sea-travel distance from archaeological data 20
Text S7. Implementation of the genetic initial conditions in the simulations 23
Text S8. Understanding the minimum in the simulated clines 28
Text S9. Horizontal/oblique transmission 34
Text S10. Calculation of the error bars of the percentages of haplogroup K 37
Text S11. A more complicated simulation model 40
Text S12. Approximate, one-dimensional model 45
Text S13. The speed of waves of advance in homogeneous space 48
Text S14. Pre-Neolithic haplogroups in Neolithic communities 50
References 53

Text S1. Analysis of K haplotypes. Signs of spatial expansion

Text S1 is devoted to independent analyses that confirm some claims made in our main paper. Therefore, the reader interested in details on the data or the model used in the main paper can jump directly to Text S2 or S5, respectively.

As explained in the Introduction of the main paper, and as we shall see in Text S2 below, haplogroup K was virtually absent in pre-Neolithic Europe, whereas numerous Early Neolithic farmers carry haplotypes belonging to this haplogroup. This leads to the hypothesis that haplogroup K spread demically with the Neolithic wave, and we have applied this hypothesis to build the simulations reported in the main paper. Note that while the Neolithic spread could have been partially cultural (in the sense that hunter-gatherers could have contributed K individuals to farmer populations), the spread of haplogroup K, if absent in the local hunter-gatherer populations, must have been purely demic (in the sense that hunter-gatherers did not contribute K haplotypes to farmer populations). Therefore, if haplogroup K spread demically with the Neolithic front, one would expect to find signs of demographic and spatial expansion in the diversity of K haplotypes found in the Early Neolithic populations.

Our database includes 56 Early Neolithic individuals presenting mitochondrial haplotypes identified as belonging to haplogroup K (see Supplementary Data S1-S2). For 55 of these individuals, at least part of the HVS-I region had been sequenced and the sequences were available in the respective sources cited in Supplementary Data S1 (the exception is sample deb29II, from the region '5 Western Germany LBK', for which the sequence for the HVS-I region could not be determined[1]). The range shared by all sequences spans nucleotide positions 16106-16390. Because the HVS-II region is not sequenced for all individuals, and different authors test different coding region SNPs, in this section we shall apply our analyzes over this HVS-I range (see Supplementary Data S7).

Therefore, in this section we shall study only the 55 Early Neolithic individuals in Supplementary Data S1 identified as presenting haplogroup K and for which the HVS-I region has been sequenced, and apply some statistical and phylogenetic analyses at the haplotype level to provide additional support to the hypothesis that haplogroup K spread demically with the Neolithic front. Our results will show clear signs of a recent expansion. Thus, given that haplogroup K was apparently absent from pre-Neolithic populations, and that there is no archeological record of other large demographic movements close in time to our data, the most reasonable conclusion from our results is the assumption made in our main paper that haplogroup K spread into Europe with the Neolithic front.

1) Tajima's D and Fu's F_s neutrality tests

We have analyzed the 55 sequences of Early Neolithic individuals with haplogroup K using Arlequin 3.5 [2], and computed the results for two neutrality tests: Tajima's D [3] and Fu's F_S [4]. For nucleotide positions 16106-16390 we can identify 12 different haplotypes (see Table S1 below), and we obtain significantly negative values for both statistics, $D = -2.10171$ and $F_S = -11.69788$. A negative value of *D* can be a result of selection, but it can also be due to a recent bottleneck or a process of population growth[3], and a negative value of F_s is often used as indicative of population expansion[4,5].

Therefore, those results would be consistent with a recent process of demographic expansion[3,6,7], which is to be expected if we assume that haplogroup K spread demically with the Neolithic, so that farming populations underwent a process of demographic expansion.

Haplotype	HVS-I polymorphisms (16106-16390) [a]	Number of individuals	Regions found [b]
H01	T16224C T16311C	37	1, 2, 3, 4, 5, 6, 7
H02	T16311C	2	1
H03	T16224C T16311C C16366T	3	1
H04	T16224C T16311C G16290A	1	2
H05	T16189C T16224C T16311C	4	2, 3
H06	A16166G T16224C T16311C	1	3
H07	T16172C T16224C T16311C	1	3
H08	T16224C C16261T T16311C	1	3
H09	T16224C T16249C T16311C	2	4, 5
H10	T16209C T16224C T16311C	1	4
H11	T16224C T16311C G16319A	1	4
H12	T16224C T16311C T16362C	1	11

Table S1. K haplotypes in Early Neolithic regions.

[a] Polymorphisms relative to rCRS [8].

[b] Region numbers correspond to the geographical region labels used in all figures and the Supplementary Data.

The mitochondrial region that we have used may in principle present a limitation as it does not include the polymorphic site at 16093, often used to discriminate K1a sub-haplogroups. Therefore we have repeated the analysis over the HVS-I range 16056-16390 for the 46 samples such that this range is sequenced (thus we have had to leave out of the analysis the 6 samples from '1 Syria PPNB', sample I0727 from '2 Anatolia', and samples 1CH0102 and CSA152223 from '6 North-Eastern Spain Cardial'). Using this reduced dataset we now obtain a value of Tajima's *D* not significantly different from zero at the 95% CL, which would indicate neutrality of mutations, while Fu's F_s is still significantly negative ($F_S = -7.90046$). Because Fu's statistic is especially sensitive to processes of population expansion[4,5], and since Tajima's *D* is not positive in this analysis and neither in that in the previous paragraph, these results reinforce the proposal in our main paper that the observed diversity is the result of a demographic expansion, rather than of any possible process of background selection.

2) Haplotype diversity

The analysis of the evolution of haplotype diversity[9] can also help in identifying processes of population expansion. Here we shall analyze the evolution of haplotype diversity over space to identify signs of geographical expansion, that is, a decrease in the diversity with distance from the assumed source (see e.g. reference [10]).

Because of the low number of Early Neolithic individuals with haplogroup K in our database, to increase the significance of the samples in this section (and in the following sections) we have pooled the

samples from geographically close regions (namely, regions 4-5 in Germany, and 6-7 in Iberia). We have computed the regional haplotype diversity indices using Arlequin 3.5 [2]. The results (Fig. S1) show a general decreasing trend with distance from Syria (with the exception of Anatolia, which shows relatively low haplotype diversity), and are thus indicative of a geographical spread from Syria[10]. This reinforces the conclusion in our main paper that haplogroup K spread.

The low haplotype diversity found in Anatolia is in fact consonant with the fact that most samples in Anatolia present haplotype H01 (Table S1), and could in principle indicate that the samples correspond to a single family unit. However, upon examining the source, this does not seem to be the case for three reason: (i) the samples correspond to two different sites; (ii) the analysis of the whole mtDNA sequences performed by Matieson *et al*. [11] does not seem to indicate that the individuals are directly related; and, more conclusively, (iii) they display different subclades of haplogroup K. Therefore, the low Anatolian diversity is probably due to the short nucleotide range that we are able to analyze in this study, as well as to sampling hazards. Similarly to Fig. S1, we expect the data from Anatolia will not follow the general trend in any of the regional analyses of mtDNA sequences performed in the next subsections. Below we shall find that this is indeed the case.

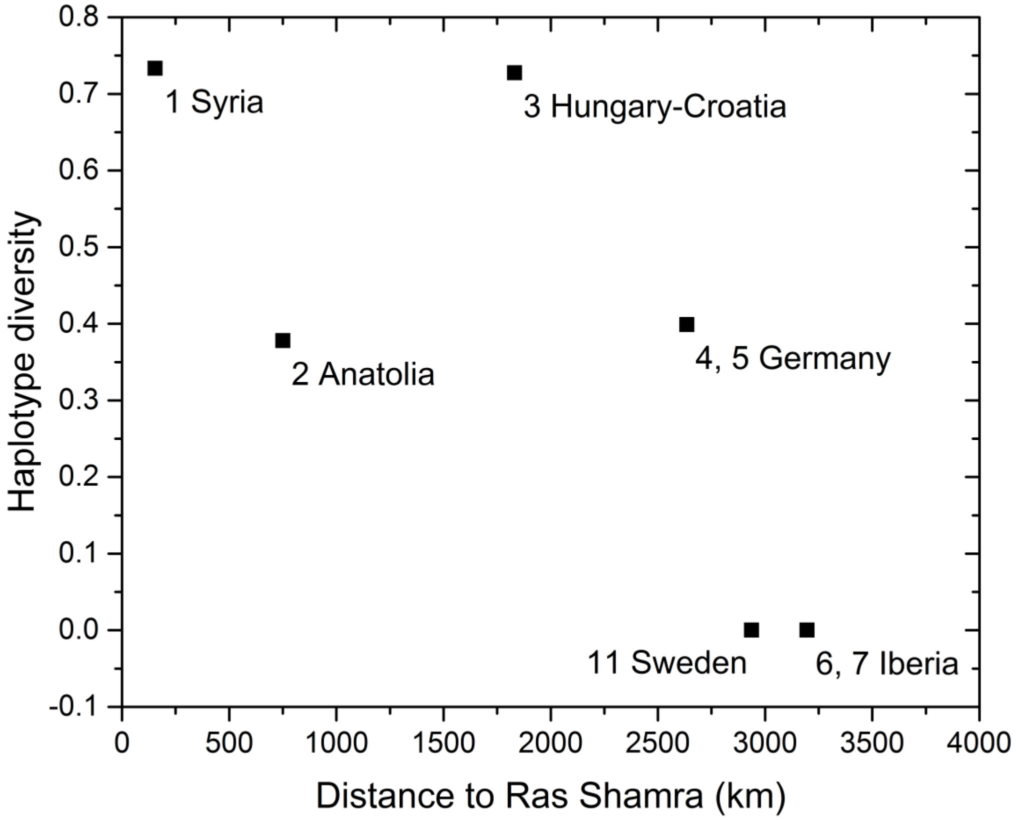

Figure S1. Haplotype diversity versus distance for Early Neolithic regions. This index shows a global decreasing trend, in agreement with a process of spatial expansion.

3) Mismatch distribution

The distribution of nucleotide site differences between pairs of individuals in a population can provide evidence of past demographic expansions undergone by this population[12]. Likewise, population range expansions can also leave similar traces in the distribution of pairwise genetic differences[13-15], with spatial signatures that can vary depending on the demic or demic-cultural nature of the expansion process[13].

Firstly, we have plotted the distribution of genetic differences including all 55 Early Neolithic individuals with haplogroup K, using the shared range of the HVS-I region 16106-16390. The result is shown in Fig. S2a. Because of the limitation of the analyzed range, the maximum number of differences if low, but the plot shows a distribution with a maximum close to zero differences, which would be consistent with a recent demographic or spatial expansion of the considered population (early Neolithic farmers with mtDNA haplogroup K)[12,15].

As we have explained at the beginning of this section (Text S1), on the basis of the genetic evidence, in our simulations in the main paper we have assumed that haplogroup K spread demically with the Neolithic wave (because it was absent in European hunter-gatherer populations). In that case, one would in principle expect differentiated mismatch distributions of K haplotypes at different regions, with a maximum closer to zero in the case of populations located further away from the source, as shown by means of simulations by Currat and Excoffier (Figure 4.a-b in their results) [13]. In Figs. S2b-f we show the mismatch distributions for different geographical regions (to increase the significance of each sample, we have pooled geographically close regions 4-5 and 6-7 as in the previous subsection; Sweden cannot be analyzed here as it has only one individual with haplogroup K). The results, while clearly limited by the low number of individuals and analyzed nucleotide positions, do show a trend in which the maximum identified in Syria (Fig. S2b) moves closer to zero at geographically distant locations (again with the exception of Anatolia, which shows a peak closer to 0 differences than expected for a region close to the source). We conclude that the general trend observed in the distributions is as expected from previous simulations[13] and can thus be interpreted as a result of a recent geographic expansion of individuals with haplogroup K. This agrees with our assumption that haplogroup K spread demically with the Neolithic front.

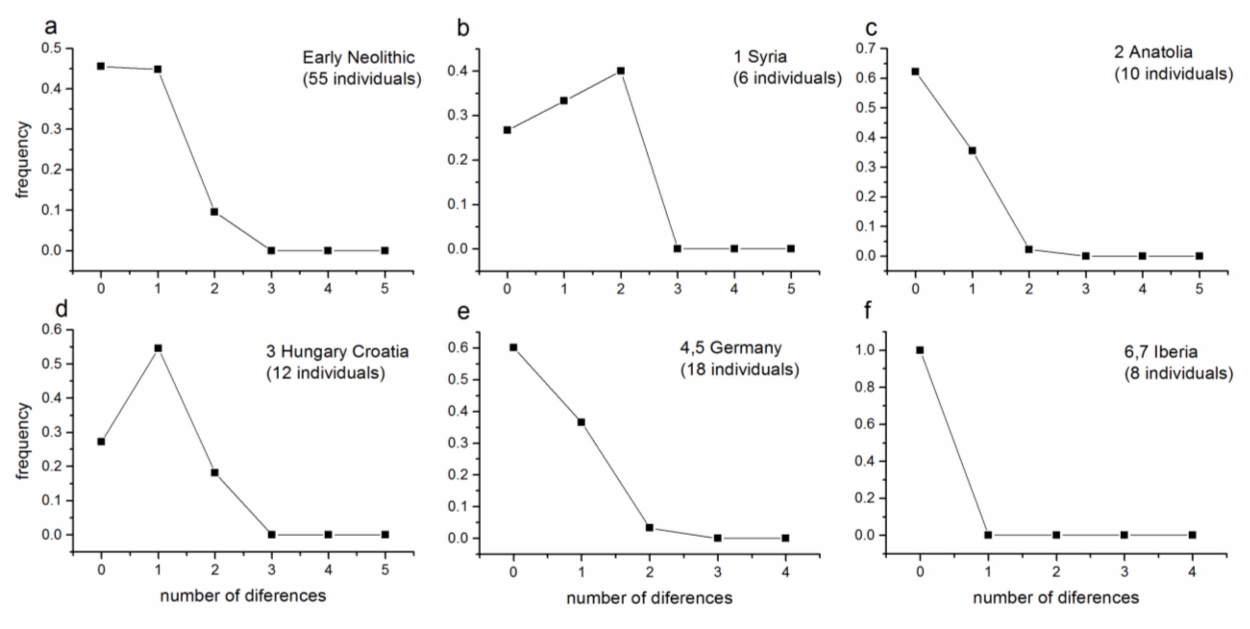

Figure S2. Mismatch distributions for K haplotypes identified in all Early Neolithic samples (upper left) and in specific regions. The distributions have been obtained from mtDNA sequences for the HVS-I region at nucleotide positions 16106-16390. (a) includes all 55 early Neolithic individuals with haplogroup K, whereas (b)-(f) correspond to regional samples.

4) Mantel test

It is well-known that a process of geographic expansion leads to a strong increase of genetic distance with increasing geographic distance[16,17]. For this reason, we have computed the pairwise genetic distance F_{ST} between the Early Neolithic regional cultures (considering only K haplotypes) and performed a Mantel test[18,19] to evaluate the correlation between genetic and geographic distance matrices[16,20,21]. Genetic distances and Mantel tests were computed with Arlequin 3.5 [2] performing 10,000 permutations. In order to increase the significance of each sample we have pooled the 55 Early Neolithic individuals presenting haplogroup K into six geographic areas (as done in the previous subsection): Syria, Anatolia, Hungary-Croatia, Germany, Iberia, Sweden.

Surprisingly, the results of applying a Mantel test to the genetic and geographic distance shows a very low matrix correlation value $R = 0.15$. Examining the data, the reason for this low value can be partially attributed to the fact that there is only a single K haplotype in Sweden, which in turn differs from all other K haplotypes analyzed, thus leading to a very high value of the genetic distance to other close regions. The sample from Sweden is also dated considerably later than the other samples (see Fig. 1 in the main paper), so the genetic distance could be due not only to geographical distance, but also to temporal distance (indeed, applying a Mantel test to genetic and temporal distances yields a much better correlation value $R = 0.67$). For this reason we have computed anew a Mantel test for genetic and geographic distances leaving Sweden out of the analysis, which leads an increases value of the correlation between matrices, $R = 0.45$.

In Fig. S3 we have plotted the genetic versus geographic distances to Syria, in order to visualize the correlation between both distances[20]. This plot shows that Anatolia diverges considerably from the overall behavior, similarly to the observation from the previous subsection where the mismatch distribution for Anatolia also diverged from our expectations. Thus we applied a Mantel test without Anatolia (nor Sweden), which leads to a much higher correlation value $R = 0.88$.

Therefore, we see that there is a spatial correlation with genetic distances, although the results when considering all regions are affected by the very late date for the sample in Sweden, and by the K samples from Anatolia, which seem to present a higher divergence than would have been expected. As mentioned when analyzing the haplotype diversity, this exception may be due to the low number of individuals and analyzed nucleotide positions in the data available at present.

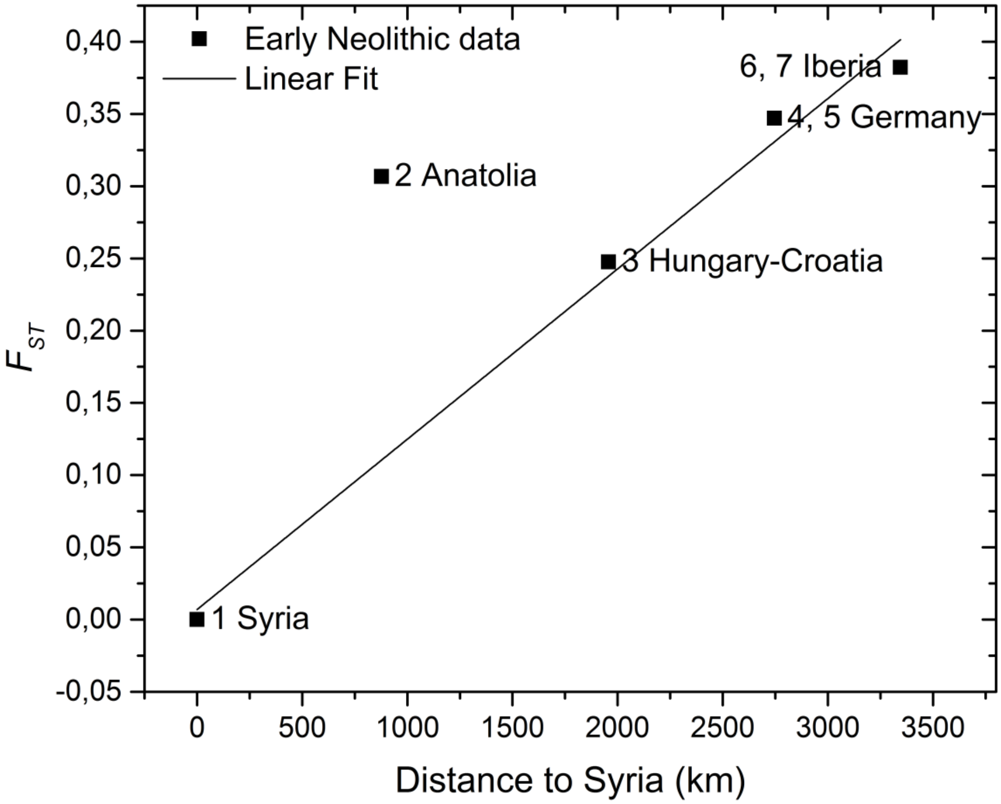

Figure S3. Genetic distances to the Syrian population versus geographic distances for Early Neolithic regions. The line corresponds to the linear fit without region 2 Anatolia. Note that these data correspond to the first column of the matrices of genetic (F_{ST}) and geographic distances used in the Mantel tests (Sweden is not included).

5) PCA Analysis

Alongside the Mantel test, we can also test the correlation between genetic and geographic distances by performing Principal Component Analysis (PCA) on the K haplotypic data. We have performed PCA between groups using PAST 3.15 software[22] for the different geographical regions (as in the previous subsections, we have pooled the data from geographically close regions).

We find that the first principal component (PC) explains a 63% of the variability between groups (the second PC explains a 22% of the variability), so below we plot the first PC against distance (Fig. S4). We see that, similarly to the results obtained above, there is a very clear spatial correlation between Syria, Hungary-Croatia, Germany and Iberia, while Anatolia (region 2) and Sweden (region 11) fall clearly out of this trend. There is a clear overall correlation between genetic differentiation and distance (Fig. S4), and this is consistent with the involvement of haplogroup K in the Neolithic demic flow.

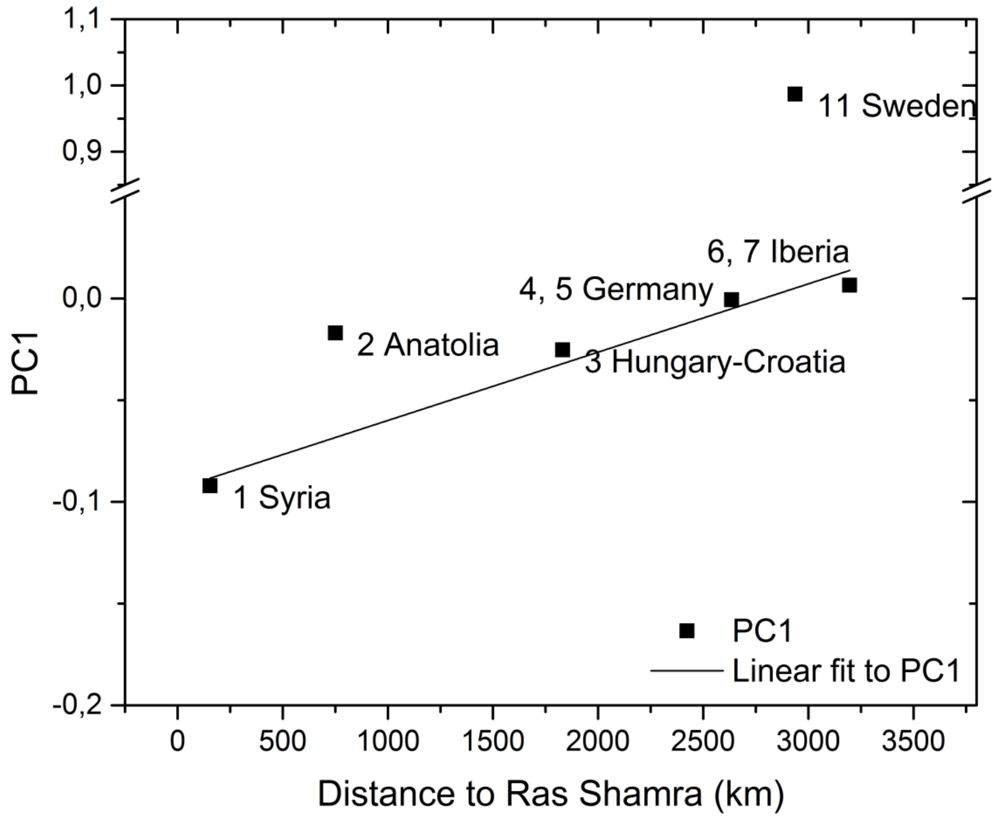

Figure S4. Variation of the first principal component (PC1) with distance from Ras Shamra. The line corresponds to the linear fit obtained excluding regions 2 (Anatolia) and 11 (Sweden).

6) Network analysis

When a population undergoes an expansion process, it has been shown that phylogenetic network analysis leads to star-shaped genealogies[23]. Figure S5 shows the median-joining network obtained with Network 5 software[24] (www.fluxus-engineering.com) for the 55 Early Neolithic HVS-I sequences (nucleotide positions 16106-16390). The obtained results are clearly star-shaped, although to reinforce this observation we have computed the star index introduced by Torroni *et al*. to evaluate the *starness* of a phylogeny[25]. This index is defined as the relative frequency of pairs of sequences that coalesce at the assumed root (in our case, haplotype H01), and a value >0.95 is considered to reflect a highly star-like group[25,26]. From the data in Table S1 we obtain that only 11 of the 1485 possible pairs of sequences do not coalesce at the root, thus the star index for the early Neolithic haplogroup K is 0.99. This indicates that we have indeed a very star-like phylogenetic network (in agreement with a process of population expansion), and that haplogroup K was involved in the Neolithic demic flow (as assumed in our main paper).

Figure S5 also provides a supplementary visualization to Table S1 above, which shows clearly that the most abundant haplotype is H01. This haplotype H01 is present in all regions but Sweden, while all of the other haplotypes are present in only one or two close regions (see Table S1). Therefore, haplotype H01 would have been carried on along the whole expansion, while other haplotypes might have appeared locally but not spread in the process of spatial expansion.

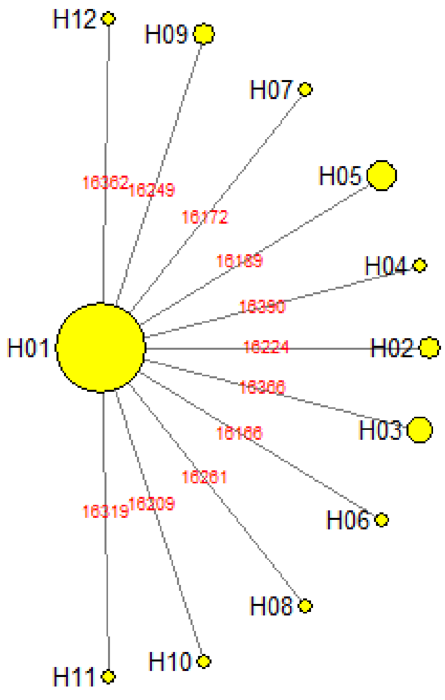

Figure S5. Median-joining network of K haplotypes present in Early Neolithic cultural regions. The nodes correspond to the haplotypes listed in Table S1 and their sizes are proportional to the number of individuals. The mutated nucleotide positions are indicated at the links.

7) Bayesian Skyline Plot

In this section, we have applied Bayesian coalescent inference to study the variation in time of the effective population of individuals bearing K haplotypes at the Early Neolithic front. We have generated a Bayesian skyline plot (BSP) [27] for the Early Neolithic HVS-I sequences (positions 16106-16390) corresponding to individuals carrying K haplotypes, each one dated with its calibrated date (see Data S7). The BSP was generated using BEAST 2 [28] and Tracer 1.6 [29]. The Markov chain Monte Carlo (MCMC) samples were based on a run of 40,000,000 generations, sampled every 40,000 generations, and with the first 10% discarded as burn-in. We used a JC69 substitution model (although using a HKY yields very similar results) and a strict clock with a mutation rate 1.62×10^{-7}, as reported by Soares *et al*. [30] for the HVS-I region.

Figure S6 shows the BSP obtained for the Early Neolithic individuals presenting haplogroup K. Because the individual with haplogroup K in Sweden is dated about 2,000 yr later than the other Early Neolithic data (see Supplementary Data S7), we have not included Sweden in the results shown in Fig. S6. Figure S6 shows that the effective population size remains mostly stationary with a decreasing trend throughout the considered period. Whereas the Neolithic spread is associated with a process of population growth, we have seen in the main text (and we shall further discuss in Text S4) that the percentage of the population carrying K haplotypes decreased at the Neolithic front, thus a stationary evolution of the population size of haplogroup K is a reasonable result. In addition, while rapid population growth processes are often related to the retention of genetic diversity[31-33], stationary populations (Fig S6) have been related with a loss of haplotype diversity[34], in agreement with our observations from Fig. S1.

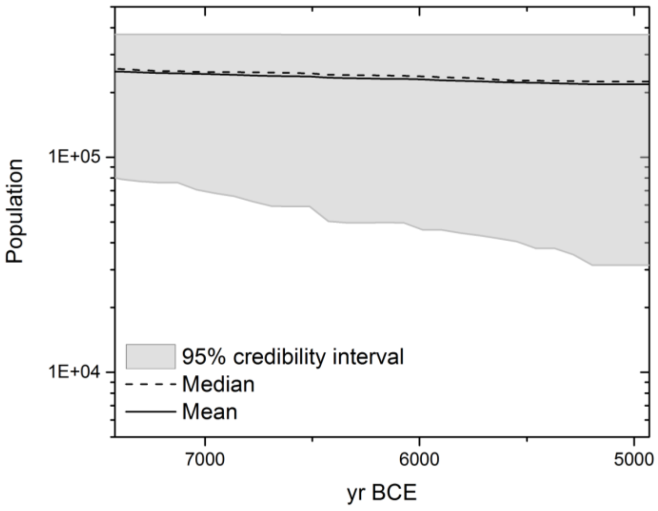

Figure S6. Bayesian skyline plot showing the evolution of the effective population size of K haplotypes in Early Neolithic groups in through time. Sweden is not included because its single date is from 2,000 yr after the youngest extreme of the range in this figure. The solid and dashed lines indicate, respectively, the mean and median population sizes, and the shaded region corresponds to the 95% credibility interval.

Text S2. Mesolithic samples with haplogroup K

As explained in the main paper: (i) haplogroup K has been found in ancient farmers in many sites of Europe, as well as in Anatolia and the Near East; (ii) in contrast, no Western neither Central European hunter-gatherer has been found so far with haplogroup K before the Neolithic period; (iii) there are very few cases of hunter-gatherers with haplogroup K. For reasons (ii) and (iii), it is very reasonable to consider haplogroup K as virtually absent in pre-Neolithic Europe. Still more, there are even reasons to disregard the very few cases of hunter-gatherers with haplogroup K mentioned in point (iii). We explain these reasons in this section. Up to date, a total of 8 Mesolithic individuals with haplogroup K have been found. One is from Germany, four from Sweden, two from Greece and one from Georgia. We discuss them in turn.

One hunter-gatherer (*OstorfSK28a*) with haplogroup K (no subclade was reported by Bramanti *et al.* [35]) was found in Ostorf, a Mesolithic site in northern Germany, and dated 3,200 cal BCE. However, as noted by Bramanti *et al.* [35], it is very remarkable that Ostorf is a Mesolithic enclave surrounded by farmers (of the Funnel-beaker culture). Moreover, Ostorf is precisely the single hunter-gatherer site where individuals with non-U mtDNA haplogroups were found[35]. Thus, it is reasonable to consider the possibility that haplogroup K was introduced in Ostorf by interbreeding with farmers.

Four hunter-gatherers (*Ire9, Fri28, GE76, Vis7B*) have been found in Sweden (Pitted Ware culture, PWC) with subclades K1a and K1a1, and dated 3,200–2,400 cal BCE [36,37]. Despite its hunter-gatherer economy, the PWC overlapped chronologically with farmers during almost a millennium, first of the Funnel-beaker culture (*Trichterbecherkultur*, TRB) and later of the Battle Axe complex, a variant of the Corded Ware culture[38,39]. This is why some authors refer to the PWC as 'Neolithic' hunter-gatherers[40]. Thus it is again reasonable to consider the possibility that this small sample of hunter-gatherers with haplogroup K (4 of 32 PWC individuals) is due to interbreeding with contemporaneous farmers living in the same region.

Two hunter-gatherers (*Theo1* and *Theo5*) displaying subclade K1c were discovered in Theopetra, a site in Thessaly (Greece) and dated 7,605–6,771 years BCE [41]. However, subclade K1c (as well as subclades K2b and K2c) has been never found among Neolithic farmers to date. Thus these two Mesolithic individuals do not affect the subclades of haplogroup K that were presumably introduced into Europe by the Neolithic population wave of advance.

Similarly, a hunter-gatherer (*Satsurblia*) from Georgia (associated with the Epigravettian culture) has been dated 11,380–11,130 cal BCE [42] and displays the K3 subgroup, which has been never found among Neolithic farmers to date.

In view of these considerations, current evidence makes it very reasonable to believe that haplogroup K or, more precisely, the subclades of haplogroup K that have been found in European Neolithic individuals (see Supplementary Data S1 for the complete list), were absent in Europe before the spread of farming, and were introduced there by incoming farmer populations of Near Eastern origin.

Text S3. Neolithic individuals not included in the study

In this work, we have gathered a database of all individuals from farming cultures dated between 8,000 and 3,000 calibrated years BCE for which the mtDNA haplogroup have been reported in the literature. We have grouped these individuals into regional cultures according to their geographical and cultural closeness, but we have only selected for further analysis (Fig. 1) the 26 regional cultures with more than 2 individuals (Supplementary Data S1). Therefore, we have discarded 5 individuals from the database. In particular, we discarded 'Spain (Valencia and Alacant)' with only two individuals[43], and all data from Greece, because the one Early Neolithic individual is dated about 2,000 yr earlier than the two Late Neolithic individuals, and therefore they cannot be considered a single group (Supplementary Data S1).

Very recently, the first mtDNA data from ancient farmers in the southern Levant (Jordan and Israel) have been reported[44]. As mentioned in the main paper, we have not included them. The reason is that haplogroup K has been found in only 23% (3 of 13) PPNB/C individuals[44], and this is substantially lower than the value 40% that we obtain for the Syrian PPNB sites[45]. If future studies (based on larger databases) confirm a low %K in the southern Levant, it may have several causes. One possibility is simply that, as suggested by the genetic analyses by Lazardis *et al.* [44], the ancient farming population from the southern Levant did not lead to the Early Neolithic populations in the Near East and Europe. A second possibility is that a drift effect could have increased the %K during the spread of the Neolithic from the Southern Levant to northern Syria. This second possibility is an open issue and would, in any case, require a substantially more complicated model (based on additional assumptions), which is out of the scope of the present paper. Thus we consider ancient mtDNA data from Syria, Anatolia and Europe, which (as we have seen) do show a fairly gradual spatial decrease (i.e., a cline) in the %K, in agreement with our simple model. Admittedly, we expect that future work will lead to more general models that can describe more complicated clines.

Text S4. Geographic cline of haplogroup K

Similarly to Text S1, this section presents some analyses that are independent of the method used in the main paper but reinforce an important claim made in our study.

In the main text (Figs. 2-3) we have visualized the geographic cline of haplogroup K by representing its measured relative presence in different regions as a function of the great-circle distance to Ras Shamra (Syria), the oldest PPNB archaeological dating in reference[46] (the great-circle distance is the shortest distance between two points on the surface of a sphere; in this case, on the surface of the Earth). This representation is the most effective option to take into account the effect of low samples (since we take into account the whole 80% CL range, plotted as error bars) and to compare the simulation results with the measured results (e.g., Fig. 3). However, it might not be the most intuitive way to understand the distribution of haplogroup K throughout the European continent. In Fig. S7 we have represented the locations of the 9 Early Neolithic regions used in Fig. 3, labeled with the percentage of population presenting haplogroup K, which we have interpolated using Ordinary Kriging with the software ESRI ArcGIS 10.4. The interpolation results show clearly that there is a spatial gradient on the presence of K haplogroup, both along the Mediterranean as well as along the interior spread route.

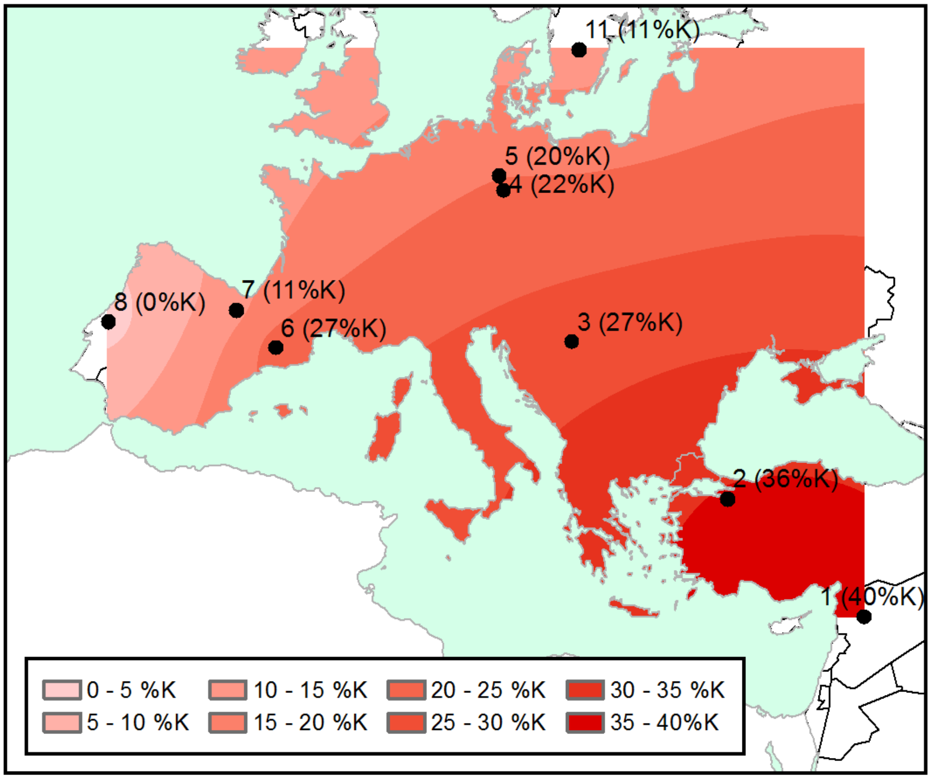

Figure S7. Spatial gradient of hapogroup K in Early Neotlihic populations. Circles represent the location of the 9 Early Neolithic cultural regions shown in Fig. 3, labeled with the %K in each of them. The kirging interpolation shows the spatial decrease in the presence of K haplogroups away from Syria. Map created with ArcMap 10 and the Spatial Analyst 10 extension (http://desktop.arcgis.com/es/desktop/).

An alternative technique to detect the presence of a spatial cline is by studying the spatial autocorrelation of the data through a Moran's I correlogram[47], as has been previously done to analyze geographic patterns from genetic data[48]. When the data display a spatial cline, the correlogram should show a decreasing behavior, with positive autocorrelation at short distances and negative autocorrelation at long distances[47,48] (i.e., nearby points are similar whereas distant points differ). On the other hand, a random spatial distribution of observed values (i.e., a non-clinal pattern) would display a flat correlogram with an expected value of Moran's I given by (see reference [47], Eq. (13.6))

$$E(I) = -1/(N-1), \tag{S1}$$

where N is the number of data points (9 regions in our case, so $E(I) = -0.125$). Note that, for a random (thus non-clinal) spatial distribution of observed values, $E(I) \rightarrow 0$ if $N \rightarrow \infty$ [47].

Figure S8 shows the correlogram obtained with PASSaGE 2 [49] for the %K present at the same 9 Early Neolithic regions as in Fig. 3 and Fig. S7. We have grouped the great-circle distances between pairs of

regions into 6 distance classes (in agreement with Struge's rule; equation 13.3 in reference [47]), chosen so that there is an equal (or nearly equal) number of observations per class. The correlogram is significant over the entire range of classes (P<0.005 Bonferroni corrected[47,50]) and shows a clinal trend, as expected if there is a spatial gradient of the presence of haplogroup K [48]. Repeating the same computation but using 6 distance classes of equal width, also yields a significant cline (results not shown; P<0.05 Bonferroni corrected).

Therefore, the results obtained here reinforce our conclusion from Figs. 2-3 that there is a spatial cline in the percentage of Early Neolithic farmers carrying haplotypes from haplogroup K.

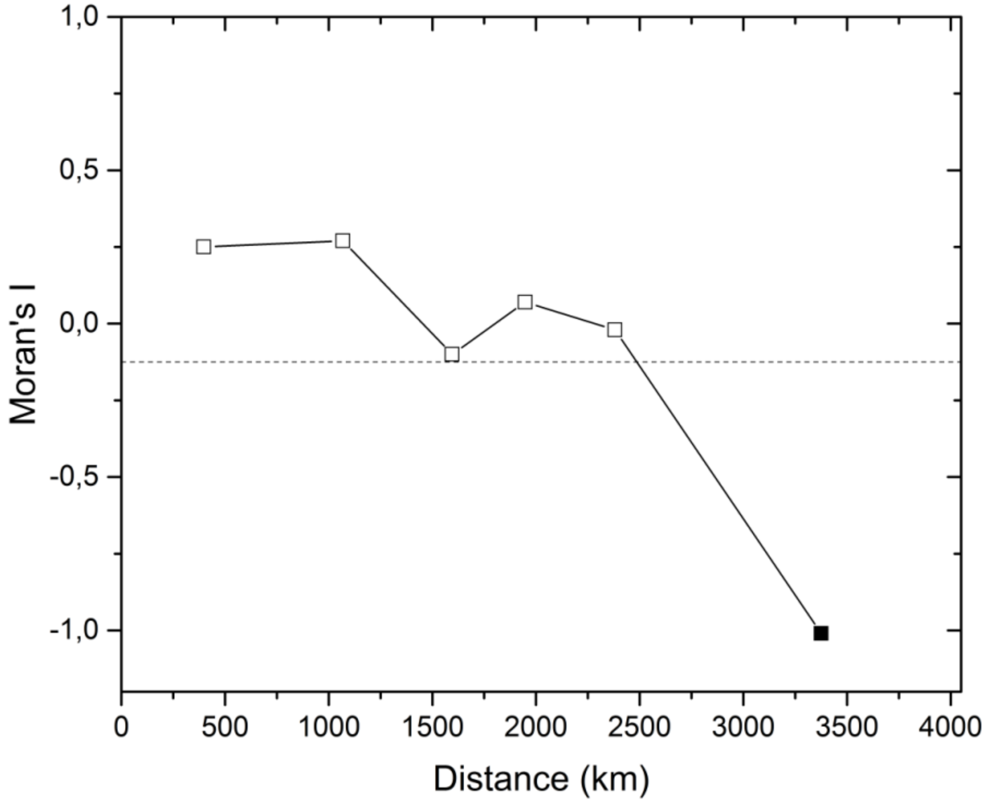

Figure S8. Spatial correlogram for the presence of haplogroup K in Early Neolithic cultural regions. The dashed line shows the expected value of I under a random (i.e., non-clinal) spatial distribution, $E(I) = -0.125$, from equation (S1) (see reference [47], Eq. (13.6)). Black dots correspond to class-specific significant values. The correlogram is significant over the entire range of classes (P<0.005 Bonferroni corrected[47,50]) and displays a clinal behavior.

Text S5. Mathematical details of the computational model

The Fortran code for the model used in the main paper, and described below, is available as Program S1 at the journal web or at http://copernic.udg.es/QuimFort/2017_08_07r__Program_S1.zip.

As explained in the Materials and Methods section, the model runs on a grid of 50x50 km^2 square cells (180x120=18,360 cells). Elevation data from the SRTM30 near-global elevation model were used to determine the main type of terrain (inland, mountain, coast or sea) of each cell[46]. For coast cells, one of the four nearest neighbors must be a sea cell, while inland cells cannot have a sea cell as one of its nearest neighbors. Neolithic and Mesolithic individuals can only inhabit inland or coast cells. Each of these cells can have a maximum famer population of $P_{F\,max} = 3{,}200$ individuals/cell [46], which includes farmers with and without haplogroup K (this value was computed from the ethnographic data on the maximum density[13], 1.28 individuals/km^2, and the area of the cell, 2,500 km^2), and a maximum hunter-gatherer population $P_{HG\,max} = 160$ individuals/cell (obtained from the ethnographic maximum density[13], 0.064 individuals/km^2). Here we consider areas higher than 1,750 m above sea level as mountain barriers. However, the results are very similar changing the value of 1,750 m by other values, and also if neglecting mountain effects altogether, as previously observed for non-genetic simulations[46].

Each cell is assigned an initial population of farmers with haplogroup K, $P_N(x, y, t = 0)$, farmers who do not have haplogroup K, $P_X(x, y, t = 0)$ and hunter-gatherers $P_{HG}(x, y, t = 0)$, as follows. Initially, $P_{HG} = 0$, $P_N + P_X = P_{F\,max}$ at the cell with coordinates (112, 31) that contains Ras Shamra, the oldest PPNB site in Syria (the values of P_N and P_X will depend on the parameters used; see details in Text S7), and $P_{HG} = P_{HG\,max}$, $P_N = 0$ and $P_X = 0$ at all other cells. Given these initial conditions, the model updates each of the three populations (N, X, HG) at every iteration (generation of 32 yr [51]) $t = 1,2,3 \ldots$, according to three steps: dispersal, interaction, reproduction (changing the order of these 3 steps would yield the same results). Note that at any instant, the total farming population per cell is given by $P_F(x, y, t) = P_N(x, y, t) + P_X(x, y, t)$. Each of the three populations considered in the model would comprise several different haplotypes, but since we are only interested in there results at the haplogroup level, we do not further subdivide the population. All computations are performed using real values, though we expect that, in average, we would obtain the same results if we used a stochastic procedure to approximate them to integers at each of the following three steps of the process.

1) Dispersal

Under the reasonable assumption that farmers have the same dispersal behavior independently of their mtDNA haplogroup, in this step we apply the following rules to each of both subpopulations.

Persistency. A fraction p_e of the subpopulation initially present at each cell remains in it (p_e is called the persistence in demography). The rest (fraction $1 - p_e$) moves to other cells, as follows. In the model we use the mean value $p_e = 0.38$ obtained from ethnographic data[52].

Land travel. The farmers that move from a cell (which may be inland or coast) can travel by land to some of its four nearest neighbor cells. We could consider a set of more than two inland travel distances (0km and 50km in our model) and their corresponding probabilities, with all distances and probabilities

estimated from ethnographic data[53,54], but this would require substantially more computer time, and we expect it would lead to similar results (so we consider only the characteristic distance moved per generation according to ethnographic data, namely 50 km [52]). As said above, Neolithic populations can only settle on inland or coast cells (mountain cells cannot be inhabited and act as barriers that cannot be penetrated; sea cells cannot be inhabited either, but allow individuals to travel by sea to other locations). Therefore, if none of the four nearest neighbors to an inland cell are mountains, each of the 4 inland or coast neighbors receives 1/4 of the population that relocates, i.e. a fraction $(1 - p_e)/4$ of the population at the initial inland cell. If one of the neighbors is a mountain, it acts as a barrier, and no population will move to this cell; as a result, each inland or coast neighbor receives a fraction $(1 - p_e)/3$. Similarly, if two of the nearest neighbors are mountains, each remaining inland or coast cell receives a fraction $(1 - p_e)/2$. In general, the fraction of the population that moves to *each* inland or coast neighbor is given by

$$\frac{(1 - p_e)}{(4 - \#mountain\ neighbors)}. \tag{S2}$$

Sea travel. Consider population leaving a coast cell. If only one of its neighbors is a sea cell, the fraction of the population that would travel by land to this cell (according to equation (S2)) travels by sea to other coast cells. If the initial coast cell has two sea neighbors, the fraction of the population that travels by sea is twice the value given by equation (S2), i.e. the number of individuals that would travel by land to both sea cells. In general, the *total* fraction of the population that travels by sea from a given cell is

$$\frac{(1 - p_e) \cdot \#sea\ neighbors}{(4 - \#mountain\ neighbors)}. \tag{S3}$$

For example, if a coast cell has one sea neighbor, two coast neighbors and one mountain neighbor, according to equation (S2) each coast neighbor would receive a fraction $(1 - p_e)/3$ of the population in the origin cell, and, according to equation (S3), an equal fraction $(1 - p_e)/3$ would travel by sea. As another example, if a coast cell has two coast and two sea neighbors, according to equation (S2) each coast neighbor would receive a fraction $(1 - p_e)/4$ of the population in the origin cell, while now a fraction $(1 - p_e)/2$ would travel by sea, according to equation (S3).

Sea travel takes place in straight lines across the sea to other coastal cells within a given range. We select as sea-travel destinations all coastal cells within the sea-travel range (measured along straight lines), that can be reached following a linear route that crosses only sea cells; i.e. those coastal cells within line of sight across the sea (and within the maximum sea travel distance). Each possible destination receives an equal fraction of the population that travels by sea. Therefore, if there are for example 5 possible destinations, each one receives 1/5 of the fraction of the population that travels by sea, which is given be equation (S3). In the simulation we use a sea travel range of 150 km. See Text S6 for details on how we determined this range.

We do not update the number of HGs at each node due to their dispersal, because the exchange of HGs between saturated cells has no effect (since the HG population lacks haplogroup K) and we assume that

they do not disperse appreciably into cells in which their number is lower than the saturation value (due to cultural transmission, see below).

2) Cultural transmission

After dispersal, in each cell there is a population of $P_{HG}(x,y,t)$ hunter-gatherers and a population of $P_F(x,y,t)$ farmers. As mentioned above, $P_F(x,y,t) = P_N(x,y,t) + P_X(x,y,t)$, with $P_N(x,y,t)$ the number of farmers who have haplogroup K and $P_X(x,y,t)$ the number of farmers who do not have haplogroup K. As mentioned in our main paper, for simplicity we consider only interbreeding (vertical transmission), but we would reach the same conclusions if we considered, instead, acculturation (horizontal/oblique transmission), or both interbreeding and acculturation (see Text S9 for a detailed justification of this point). Under vertical transmission, to determine the population that will conform the new generation, we have to compute the matings that take place between and within those 3 population groups, and then apply the reproduction step.

Cross-matings between cultural groups. We assume that children of cross matings between farmers and HGs are farmers, in agreement with ethnographic observations[55,56]. The number of cross matings between HGs and farmers is then given by[57]

$$couples\ HF = \eta \frac{P_{HG}(x,y,t) \cdot P_F(x,y,t)}{P_{HG}(x,y,t) + P_F(x,y,t)}, \quad \text{(S4)}$$

where $P_{HG} + P_F = P_{HG} + P_N + P_X$ is the total population present at the cell, and parameter η is the intensity of interbreeding[57]. The value of the interbreeding parameter lies in the range $0 \leq \eta \leq 1$, with the case $\eta = 1$ corresponding to random mating ($\eta > 1$ would correspond to more cross matings that under random mating, which is not realistic for farmers and HGs according to ethnographic data[56,58] and, moreover, $\eta > 1$ can lead to $P_{HG} < 0$ for $P_{HG} \ll P_F$ [57]).

Here we are interested in the genetics of the offspring. In order to compute this, we need to consider separately the matings of HGs and farmers who have (P_N) or not (P_X) haplogroup K. Therefore, we separate the number of matings given by equation (S4) into two terms,

$$couples\ HN = \eta \frac{P_{HG}(x,y,t) \cdot P_N(x,y,t)}{P_{HG}(x,y,t) + P_F(x,y,t)}, \quad \text{(S5)}$$

$$couples\ HX = \eta \frac{P_{HG}(x,y,t) \cdot P_X(x,y,t)}{P_{HG}(x,y,t) + P_F(x,y,t)}. \quad \text{(S6)}$$

Note that $couples\ HF = couples\ HN + couples\ HX$, since $P_F = P_N + P_X$.

Within each population, the number of individuals who do not take part in HN neither HX matings is given by

$$P'_{HG}(x,y,t) = P_{HG}(x,y,t) - couples\ HN - couplesHX, \quad \text{(S7)}$$

$$P'_N(x,y,t) = P_N(x,y,t) - couples\ HN, \quad \text{(S8)}$$

$$P'_X(x,y,t) = P_X(x,y,t) - couples\ HX. \tag{S9}$$

Cross-matings between genetic groups of farmers. Let us next compute the number of matings between *farmer* individuals of different genetic groups, i.e. between populations P'_N and P'_X. Again, we can compute the number of mixed genetic couples using vertical cultural transmission theory. However, we have no reason to assume that farmers of a genetic group will have a preference for (neither against) mating with farmers of the same genetic group. Thus we apply random mating ($\eta = 1$) [57] for matings between farmers. Therefore, the number of NX matings is

$$couples\ NX = \frac{P'_N(x,y,t) \cdot P'_X(x,y,t)}{P'_N(x,y,t) + P'_X(x,y,t)}. \tag{S10}$$

Note that we are indeed dealing with an equation equivalent to equation (S4), although now the total population we are considering is just the farmer population that does not mate with HGs, i.e. $P'_N + P'_X$. This completes the computation of the numbers of all possible cross-matings.

Matings within groups. All remaining individuals, i.e. those that do not mate with individuals of a different group, will mate with individuals of their group (individuals that do not mate are not explicitly considered, since their effect is already taken into account by the net reproduction rate used in the next step). In these cases, obviously there are 2 individuals of the same group per mating, and the corresponding numbers of matings are

$$couples\ HH = P'_{HG}(x,y,t)_{HG}/2, \tag{S11}$$

$$couples\ NN = [P'_N(x,y,t) - couples\ NX]/2, \tag{S12}$$

$$couples\ XX = [P'_X(x,y,t) - couples\ NX]/2. \tag{S13}$$

where we have taken into account that matings NX have 1 N individual and 1 X individual, so the number of individuals N (or X) in matings NX is equal to the number of couples NX.

3) Reproduction

Finally, we apply reproduction to compute the new populations at each node a generation later. To do so we set the following rules. (i) Each couple will have $2R_{0,i}$ children, because $R_{0,i}$ is computed per individual and there are two individuals per mating. However, the net growth rate $R_{0,i}$ is different for farmers than for HGs ($i = F, HG$). Ethnographic data indicate that the children of cross-matings with one HG parent are farmers[55,56], thus we use $R_{0,HG}$ for matings in which both parents are HGs, and $R_{0,F}$ for HN, HX, NN, XX and NX matings. (ii) For each kind of mixed genetic matings (HN and NX), in our simplest model we assume that the mother is N in 50% of matings, i.e. that 50% of the children from genetic mixed matings have haplogroup K (because mtDNA is inherited from the mother, and thus only the offspring from mothers bearing haplogroup K will have this haplogroup). Classical cultural transmission theory[59] assumes that $R_{0,F} = R_{0,HG} = 1$ (no population growth) but this is not our case, because we are dealing with a population expansion of farmers, so their number increases and we used instead $R_{0,F} = 2.45$, obtained from ethnographic data[54]. Under assumptions (i) and (ii), the number of individuals of each population group the next generation is related to the numbers of matings as

$$P_{HG}(x,y,t+1) = R_{0,HG}[2 \cdot couples\ HH], \tag{S14}$$

$$P_N(x,y,t+1) = R_{0,F}[2 \cdot couples\ NN + couples\ NX + couples\ HN], \tag{S15}$$

$$P_X(x,y,t+1) = R_{0,F}[2 \cdot couples\ XX + 2 \cdot couples\ HX + couples\ NX + couples\ HN]. \tag{S16}$$

where the factor 2 before the number of couples HH, NN and XX comes from the fact that each of those matings leads, the next generation, to $2R_{0,i}$ individuals of the same group as their parents. Similarly, the factor 2 in front of the number of couples HX takes into account that each such mating leads to $2R_{0,F}$ farmers of genetic type X (with haplogroups different than K) the next generation. In contrast, each of NX or HN matings leads to $R_{0,F}$ farmers of genetic type N and $R_{0,F}$ farmers of genetic type X, because of assumption (ii), so the factor 2 does not appear before the number of such couples. Finally, although this is not necessary to perform the simulations, we can relate the population numbers at generation $t+1$ to those at the previous generation t by using equations (S7)-(S9) into equations (S11)-(S13), and the results into equations (S14)-(S16). This yields

$$P_{HG}(x,y,t+1) = R_{0,HG}[P_{HG}(x,y,t) - couples\ HX - couples\ HN], \tag{S17}$$

$$P_N(x,y,t+1) = R_{0,F}\ P_N(x,y,t), \tag{S18}$$

$$P_X(x,y,t+1) = R_{0,F}[P_X(x,y,t) + couples\ HX + couples\ HN]. \tag{S19}$$

Besides this mathematical derivation of equations (S17)-(S19), it is also important to understand intuitively why, e.g., the number of couples *NX* does not appear in equations (S18)-(S19). The reason is that, although each *NX* couple implies that, e.g., one N individual less takes part in *NN* couples, i.e. that there are $R_{0,F} couples\ NX$ individuals of type N less the next generation, this is compensated by the fact that 50% of the $couples\ NX$ will also lead to individuals of type N (due to assumption (ii) above), thus contributing $0.5(2 \cdot R_{0,F} couples\ NH) = R_{0,F} couples\ NH$ individuals of type N to the next generation (remember that each couple has $2R_{0,F}$ children). For the same reason, NX couples do not appear in equation (S19), nor do HN couples appear in equation (S19). The latter do appear in equation (S19) because a HN couple does not imply that one X individual less takes part in *XX* couples, and thus its effect is not compensated. Couples *HX* do not appear in Eq. (S18) because all offspring of *HX* couples are farmers without haplogroup K, i.e. they all belong to group X (not to group N). They appear in Eq. (S19) because, although each *HX* couple implies that one X individual less takes part in *XX* couples (i.e., $R_{0,F} couples\ HX$ individuals less of type X the next generation), it also leads to $2R_{0,F} couples\ HX$ individuals of type X the next generation. We stress that equations (S17)-(S19) have been derived mathematically from equations (S11)-(S16), but we think that these explanations help to understand them intuitively.

If the number of individuals computed for some population group, cell, and time step is larger than its corresponding maximum ($P_{F\,max} = 3{,}200$ individuals/cell or $P_{HG\,max} = 160$ individuals/cell), then the simulation program sets it to the corresponding maximum value (this is applied, as in previous work[46,52], to avoid population densities above saturation, which would not be biologically realistic). If $P_N + P_X > P_{F\,max}$, then P_N and P_X are both multiplied by $\frac{P_{F\,max}}{P_N+P_X}$, so that the new values satisfy that $P_N + P_X =$

$P_{F\,max}$ and the proportion $\frac{P_N}{P_X}$ does not change. In equations (S14)-(S19), as in previous work[46,52], we do not use a logistic growth function because it could lead to negative population numbers due to the fact that we are dealing with finite-difference equations (not with differential equations)[52,60]. The solution of a logistic growth function (as applied in previous works[53,54]) could be another alternative to avoid this problem, but we expect that it would yield similar results, so we do not apply it for mathematical simplicity.

Text S6. Estimation of the characteristic sea-travel distance from archaeological data

Previous work has shown the importance of long-distance sea travel in the spread of the Neolithic along the Mediterranean coast[46,61,62]. For this reason, our simulations include sea travel as a separate dispersal mechanism, in addition to inland travel. As in previous research by several authors[52,54,55,63], we have estimated the characteristic distance of inland travel (50 km per generation) from ethnographic data for preindustrial farmers[52,55]. Sufficiently detailed ethnographic data for sea travel distances of preindustrial farmers are unfortunately unavailable. In spite of this, we have estimated the characteristic distance of sea travel in the following way. Similarly to previous work[46,62], we have required that the arrival times of the Neolithic at several regions along or near the Mediterranean (as predicted by our simulations) agree with that of the oldest archaeological data in each region, and that the spread routes correspond with those implied from archaeological data. In the simulations, sea travel takes place toward all coastal cells that can be reached in a straight line across the sea within a certain range.

For the sake of clarity, we stress that the genetic data available (Supplementary Data S1) do not necessarily correspond to the earliest Neolithic sites in each region. The reason is that the genetic data, i.e. the individuals whose mtDNA haplogroup has been determined, have later (in some cases, substantially later) dates than those of the first Neolithic sites. Therefore, in order to compare to the arrival time obtained from our simulations, we cannot use the genetic dates. Instead, we have to use the observed arrival time of the Neolithic (i.e., the oldest archaeological data of Neolithic sites in the region considered). In Fig. S9, black squares correspond to the arrival dates of the Neolithic in the eight regions where we have the oldest genetic data. Note that in the main paper, Fig. 1, each square gives the time and distance of the oldest Neolithic *genetic* data in a region, whereas in Fig. S9 each square gives the time and distance of the oldest Neolithic *archaeological* site in that region (for this reason, the dates and distances in Figs. S9 and 1 are different).The information of the dates used in Fig. S9 is listed in its caption and in Supplementary Data S4.

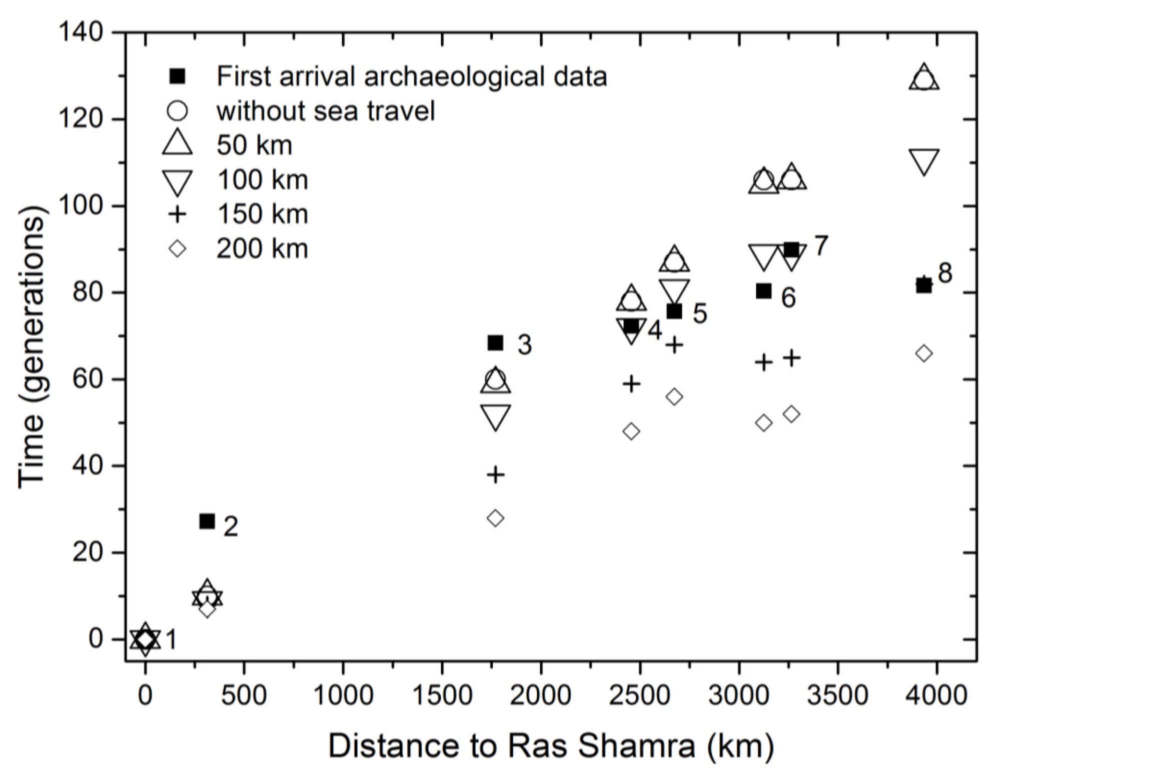

Figure S9. Estimation of the characteristic sea-travel range. Black squares: 1 oldest dates of the PPNB culture in Syria (Ras Shamra, 8,233 cal BCE [46], recall that PPNB is the Near-Eastern Neolithic culture that later spread into Europe [46]); earliest Neolithic dates in: 2 Anatolia (Hayaz Höyük, 7,361 cal BCE [46]), 3 Hungary-Croatia Starčevo (Gudnja, 6,044 cal BCE [46]), 4 Eastern Germany LBK (Dresden-Prohlis, 5,920 cal BCE [46]), 5 Western Germany LBK (Eilsleben, 5,811 cal BCE [46]), 6 North-Eastern Spain Cardial (Forcas, 5,661 cal BCE [64]), 7 Spain Navarre (Aizpea [at Basque Country], 5,357 cal BCE [64]), and 8 Portugal coastal Early Neolithic (Vale Pincel I, 5,620 cal BCE [64]). White symbols show the corresponding arrival times of our simulations with no sea travel (circles) and with sea travel of 50 km (up triangles), 100 km (down triangles), 150 km (crosses) and 200 km (rhombuses). The vertical axis is the time elapsed since the start of the simulations (8,233 BCE), measured in generations (1 generation = 32 yr [51]).

We have performed our simulations with origin at Ras Shamra (oldest PPNB site in Syria) and different sea-travel ranges, assuming no population interaction. In Fig. S9 we show these results as white symbols, which correspond to our simulations with no sea travel (circles) and with sea travels up to 50 km (up triangles), 100 km (down triangles), 150 km (crosses) and 200 km (rhombuses). The arrival time of the Neolithic into a cell is recorded by the simulations as the generation when the farmer population of the cell reaches about a 10% of its maximum (this seems a reasonable percentage because it is unlikely that the archaeological record corresponds to the earliest farmers per region, and this values is close to the minimum size required for a human reproductive network to be viable[65]; however, changing this percentage would not change our conclusions). We can see in Fig. S9 (and in Supplementary Data S4) that apparently the best agreement between archaeological data (black squares) and the simulations is attained for sea travels up to 100 km (down triangles), since it provides a lower divergence between

results. However, the results for this sea-travel range present two problems. (i) Simulations with sea travels up to 100 km arrive to regions 5, 6 and 8 later than the archaeological earliest data, which means that these results cannot really explain the earliest Neolithic evidences known, since the model arrives too late. (ii) A very important limitation of considering sea travels up to 100 km is that southern Italy is reached from the North (Fig. S10a), which is inconsistent with the archaeological dates that indicate very clearly that southern Italy was reached by sea from Albania or Greece (see Fig. 6 in reference [46]). In contrast, if we consider sea travels up to 150 km: (i) all regions are reached by the time of the earliest archeological date (see Fig. S9 and Supplementary Data S4). (ii) Crucially, southern Italy is appropriately reached before northern Italy through sea travel from Albania (see Fig. S10b). This is due simply to the fact that, in the simulation grid, the distance between the centers of the closest 50x50 km cells in Albania and Southern Italy is between 100 km and 150 km, so sea travels of at least 150 km are necessary for the front to enter Italy by this route. For reasons (i) and (ii) above, we consider that the best results are attained with sea travels of up to 150 km. It is interesting that the same result (i.e., 150 km) had been obtained previously by comparison to hundreds of individual sites (table 1 and Fig. 8 in reference [46]). More detailed models, e.g. with a different sea travel distance in the Western[62] than in the Eastern Mediterranean could be considered, but we expect that they would not change our main result (namely, that the cline of haplogroup K implies that few farmers were involved in cultural diffusion).

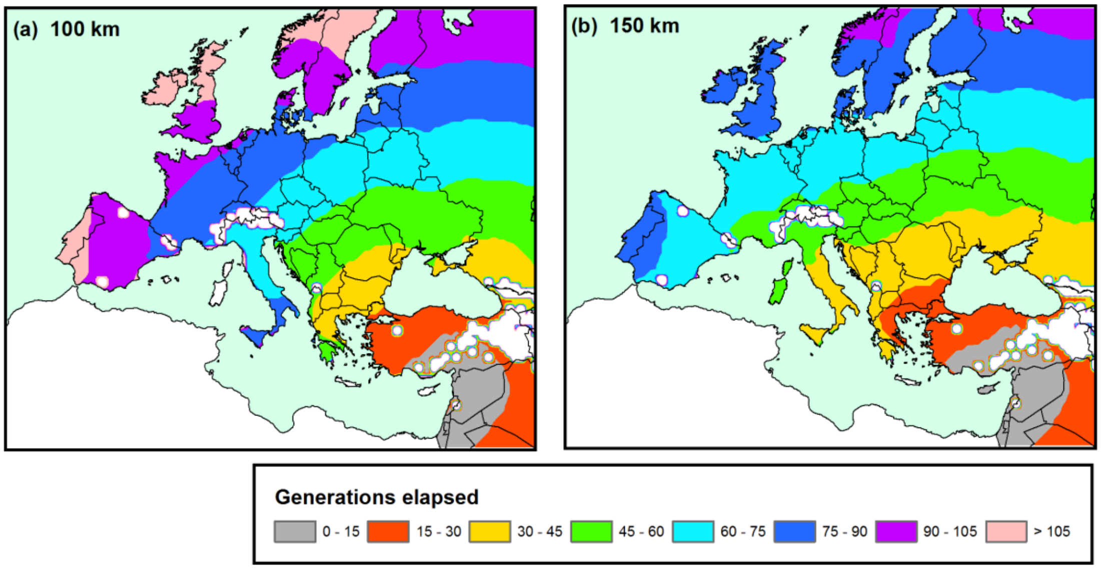

Figure S10. Predicted Neolithic arrival times computed with no interaction and for sea travel ranges of 100 km (a) and 150 km (b). White areas correspond to mountains, and the colors give the intervals of generations elapsed since the start of the simulations in Ras Shamra (Syria). Note that including interaction (cultural transmission) would not change the conclusion that the front enters Italy from the North in (a) and from the South in (b). Maps created with ArcMap 10 and the Spatial Analyst 10 extension (http://desktop.arcgis.com/es/desktop/).

Text S7. Implementation of the genetic initial conditions in the simulations

In order to compare the percentages of haplogroup K (%Ks) from our simulations to those from genetic data, we have to compute the %Ks from the simulations at the times of the genetic data (as given in the caption to Fig. S11), i.e. at the time when the fraction of ancient farmers bearing haplogroup K is known for each region (not at the time when the Neolithic arrived to it, which is obviously older and is given in the caption to Fig. S9).

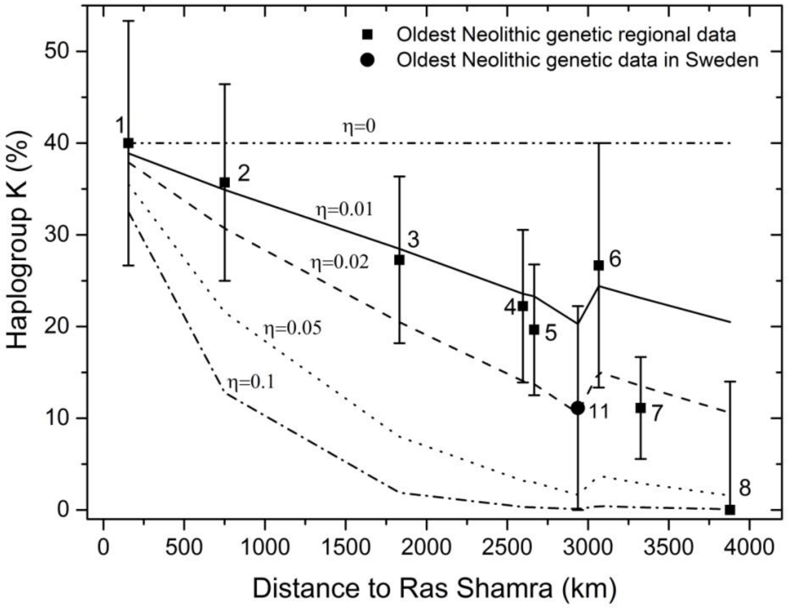

Figure S11. The lines are the model predictions when applying 40%K at the time of the oldest PPNB/C archaeological data in Syria (8,233 cal yr BCE). Symbols (with error bars) correspond to the observed percentages of haplogroup K in the 9 oldest regional cultures. Lines are the results from the simulations for different values of the interbreeding intensity η. The lines have been plotted by joining the simulation results for each of the 9 regional cultures (at its average location and date of its individuals). Here and in the rest of figures, for each regional culture, the date used to compute the results of the simulations is not that of the regional arrival of farming (as in Fig. S10) but the average date of the ancient individuals whose mtDNA haplogroup is known. In this way, we can compare simulated and observed %Ks. The regional cultures (and their average dates, as calculated in Supplementary Data S1) are: 1 Syria PPNB (7,258 cal yr BCE), 2 Anatolia (6,243 cal yr BCE), 3 Hungary-Croatia Starčevo (5,675 cal yr BCE), 4 Eastern Germany LBK (5,125 cal yr BCE), 5 Western Germany LBK (5,115 cal yr BCE), 6 North-Eastern Spain Cardial (5,286 cal yr BCE), 7 Spain Navarre (4,941 cal yr BCE), 8 Portugal coastal Early Neolithic (5,184 cal yr BCE) and 11 Sweden (2,802 cal yr BCE). The lines show the results of the simulations assuming that the %K in the Syrian region with PPNB sites was 40% at 8,233 cal yr BCE. However, according to the ancient DNA data available, this happened about 1,000 yr later (at 7,258 cal yr BCE). The problem is that in this figure, we do not obtain a 40% of haplogroup K in Syria at 7,258 cal yr BCE (see the values of the lines at region 1) except if $\eta = 0$ (no interbreeding and, therefore, no cline). In the main paper we applied a different implementation of the initial conditions to avoid this inconsistency (see Fig. S12).

As explained in the main paper (Materials and Methods), we began our simulations at the date and location of the oldest Syrian PPNB site, namely Ras Shamra at 8,233 cal yr BCE [46]. Since this location is only about 150 km away from the average location of the Syrian sites with available mtDNA data, at first sight one might expect that we could directly apply the value (40%K) measured at the latter (Supplementary Data S2-S3, estimated from the data reported by Fernández *et al.* [45]) also as initial genetic conditions at Ras Shamra. However, if we did so, we would obtain the results shown in Fig. S11. Note that in this figure the %K of PPNB Syrian sites (region 1) is not 40% but lower (except if $\eta = 0$). There are two reasons for this. The less important one is that the cell where we record the genetic information, located at the average location of the PPNB Syrian individuals in Supplementary Data S1, is 4 land-travel steps (50 km each) away from the origin of the simulation (i.e., the cell that contains Ras Shamra). Therefore, there is some interbreeding between the farmer population expanding from the original cell and the hunter-gatherer populations (which lack haplogroup K) at those other 4 cells. However, the most important reason is that the simulation starts at 8,233 cal yr BCE (the date of Ras Shamra) but we compute the simulation results (lines in Fig. S11) for Syria (region 1) at 7,258 cal yr BCE (because 7,258 cal yr BCE is the average date of all PPNB individuals whose mtDNA haplogroup is known in this region, as computed in Supplementary Data S1 from the data in reference [45]). Therefore, the fact that the %K in region 1 in Fig. S11 is below 40% (except if $\eta = 0$) is mostly due to interbreeding between farmers and hunter-gatherers during the 1,000 yr elapsed since the beginning of the Neolithic (8,233 cal yr BCE) until the time when we have genetic data to compare to the simulations (7,258 cal yr BCE). Note that the decrease in %K in region 1 (Fig. S11) is larger the more intense the interbreeding (i.e. the higher the value of η), as it should.

In order to avoid this inconsistency, i.e. in order to avoid values of the percentage of haplogroup K below 40% at region 1 at time 7,258 cal yr BCE (lines in Fig. S11, region 1), we repeated the simulations by finding (by trial and error), for each value of η, an initial value (at time 8,233 cal yr BCE) for the %K in the starting cell (Ras Shamra) higher than 40% and such that the simulations yielded 40% of haplogroup K in region 1 at time 7,258 cal yr BCE (in agreement with the genetic data[45]). The results are shown in Fig. S12, which is the same as Fig. 3 in the main paper. Note that, as opposed to the results in Fig. S11, by taking into account the time lag between the first archaeological and genetic evidence, in Fig. S12 all lines predict a 40% of haplogroup K in region 1 (at 7,258 cal yr BCE), in agreement with the genetic data (Supplementary Data S1-S3) reported by Fernández *et al.* [45]. Therefore, in Fig. S12 the observed genetic initial condition (40%K in region 1, i.e. Syria) has been applied at the correct time (7,258 cal yr BCE). In contrast, in Fig. S11 the same genetic initial condition has been applied, but at an incorrect time (8,233 cal yr BCE).

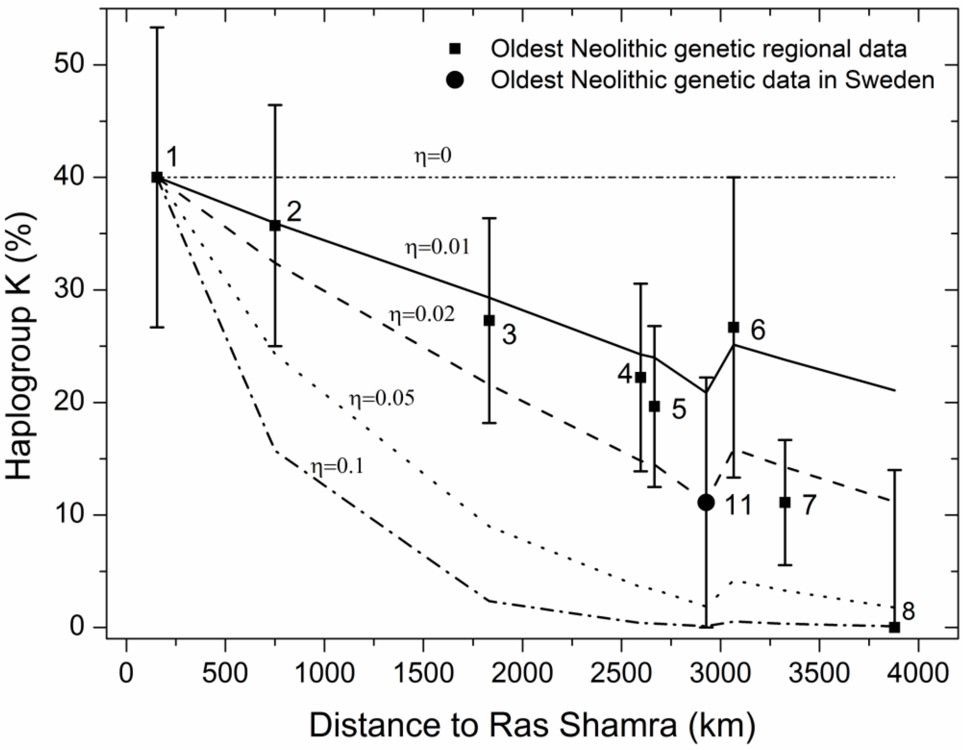

Figure S12. This figure is the same as Fig. 3 in the main paper. The lines are the model predictions when applying the adequate %K in Syria at 8,233 cal yr BCE to obtain a 40%K in Syria (region 1) at 7,258 cal yr BCE. Thus this figure shows the results of the simulations (lines) assuming that the percentage of haplogroup K in the Syrian region with PPNB sites was 40% at 7,258 yr BCE, in agreement with the ancient DNA data (symbol for region 1; percentage computed in Supplementary Data S1-S3 from the genetic data by Fernández *et al.* [45]). Compare to Fig. S11, where the %K at the initial cell is assumed to be 40% at 8,233 yr BCE instead. The regional cultures and dates are the same as in Fig. S11.

In all of our simulations, the maximum population density is 3,200 individuals/cell (see Text S5). Therefore, the initial genetic condition that at 7,258 cal yr BCE we had a 40%K in Syria (region 1) means that 1,280 of the 3,200 early farmers in this cell have haplogroup K. However, in the genetic dataset (Supplementary Data S1) we only have 6 of 15 individuals carrying K haplotypes, a value considerably lower than in our simulations. Unfortunately, we have not been able to find further aDNA data for Early Neolithic individuals in Europe to improve our dataset (beyond the 15 individuals reported by Fernández *et al.* [45], already included in Supplementary Data S1). To check the representativeness of the dataset used, we have repeated our simulation but now using as initial genetic conditions the two extreme values (maximum and minimum) of the 80% CL error bar (note that the error bars have been computed, using the bootstrap method, precisely to take into account the small size of the available samples; see Text S10).

We have first repeated the computations in Fig. S12 (or Fig. 3) but using as initial %K in Syria (at 7,258 yr BCE) the lowest extreme of the error bar (the lowest extreme of the 80% CL range), i.e. 26.67%K, as shown in Fig. S13. Under these initial conditions we see that now the best fit between model and data is obtained for $\eta = 0.01$, since it is the value of the cultural transmission parameter for which the modelled results cross most of the error bars (in fact, all of them except '2 Anatolia'). Assuming a lower intensity of cultural transmission would yield a better prediction for Anatolia, but then the predictions would fall out of the measured range for the regions furthest from the origin. Therefore, if we initially had a 26.67%K in Syria (i.e, about 850 of the 3,200 early farmers), the observed cline could be explained assuming $\eta = 0.01$, an intensity of cultural transmission lower than the value $\eta = 0.02$ obtained when using the mean %K measured for Syria. This is as expected because a lower value of η leads to a smoother cline.

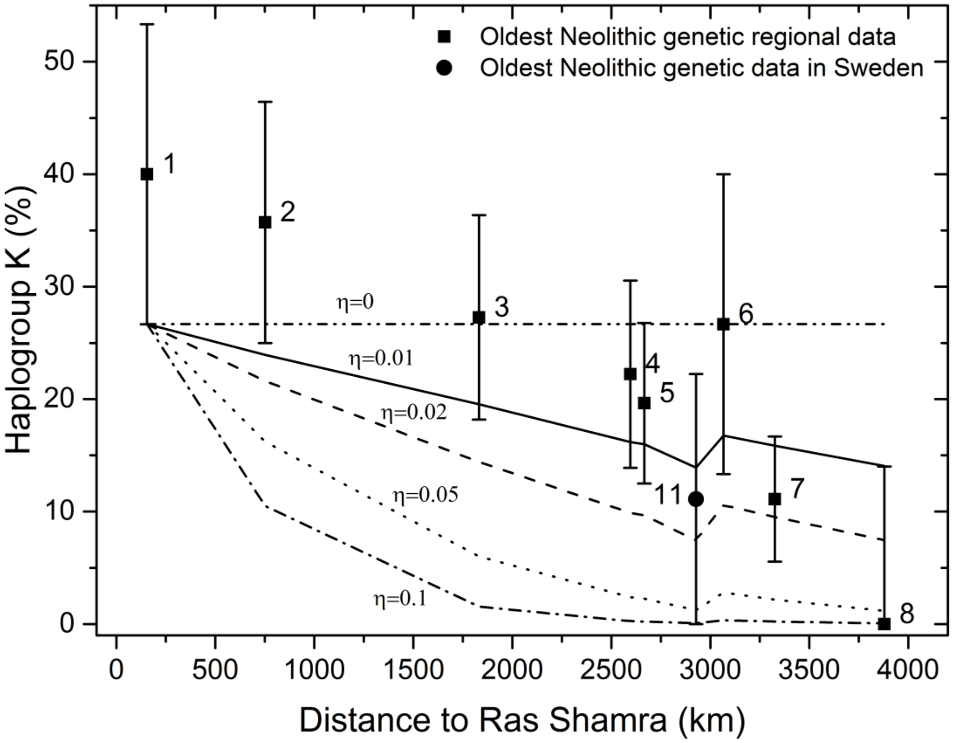

Figure S13. Model predictions when applying as initial genetic conditions in Syria (region 1) the *lower extreme* of the error bar of the observed %K (this is why the % in region 1 is not 40% but lower). This figure shows the results of the simulations (lines) assuming that the %K in the Syrian region with PPNB sites was 26,67% at 7,258 yr BCE, computed from the aDNA data as the lower extreme of the 80% CL bootstrap range (error bar for region 1; range computed in Supplementary Data S6 from the genetic data by Fernández *et al.* [45]). A good agreement with the data is obtained for $\eta \approx 0.01$. The regional cultures and dates are the same as in Fig. S11.

On the other hand, when we consider as initial genetic condition in Syria the upper extreme of the 80% CL range, i.e. 53.33%K, we see in Fig. S14 that the dashed line ($\eta = 0.02$) overestimates the percentage of the farmer population with haplogroup K at the regions furthest away from the origin. Thus, the intensity of cultural transmission needed to explain the cline is now higher than $\eta = 0.02$ (also as expected). The cline for $\eta = 0.03$, shown in Fig. S14 correctly predicts the observed percentages at the more distant populations(although it slightly underestimates the %K at regions 4 and 5). Therefore, the level of cultural transmission needed to explain the observed cline when assuming a 53.33%K in Syria at 7,258 BCE (i.e., about 1,700 of the 3,200 early farmers), is not higher than $\eta = 0.03$.

Thus, in summary, when considering the whole 80% CL range for the initial conditions, we have found that the observed genetic cline can be explained for intensities of cultural transmission in the range $\eta = 0.01 - 0.03$. Therefore, the conclusions in the main paper (that about 2% of farmers we involved in cultural transmission) is maintained (and refined by the range $2\% \pm 1\%$).

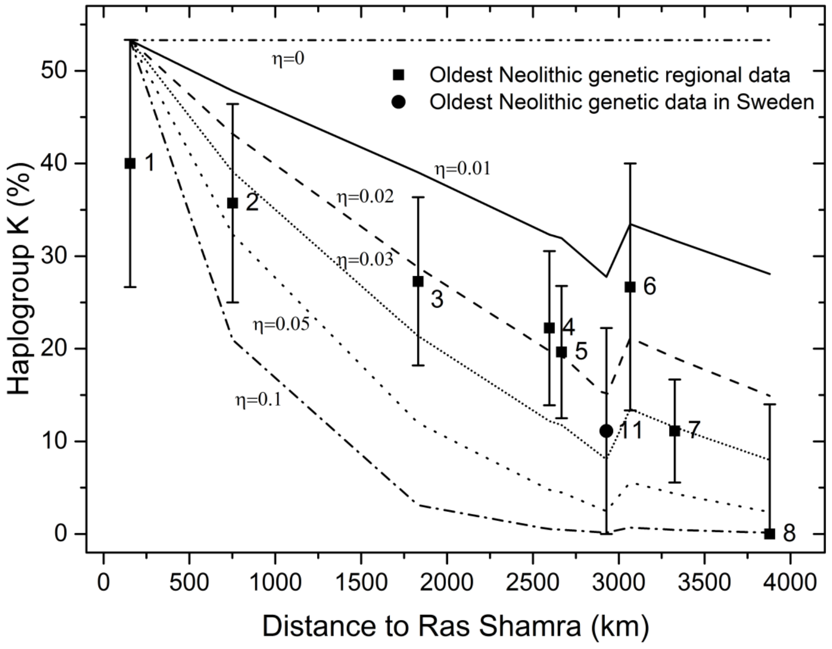

Figure S14. Model predictions when applying as initial genetic conditions in Syria (region 1) the *upper extreme* of the error bar of the observed %K (this is why the % in region 1 is not 40% but higher). This figure shows the results of the simulations (lines) assuming that the %K in the Syrian region with PPNB sites was 53,33% at 7,258 yr BCE, computed from the aDNA data as the upper extreme of the 80% CL bootstrap range (error bar for region 1; range computed in Supplementary Data S6 from the genetic data by Fernández *et al.* [45]). A good agreement with the data is obtained for $\eta \approx 0.03$. The regional cultures and dates are the same as in Fig. S11.

Text S8. Understanding the minimum in the simulated clines

In Fig. S12 (i.e., Fig. 3 in the main paper), we observe that the curves obtained from the simulations have a local minimum for region 11, i.e. Sweden. Interestingly, a minimum in Sweden is also seen for the genetic data (squares and circle in Fig. S12). The general shape of the curves in Fig. S12 is easy to understand, as follows. As the distance (horizontal axis in Fig. S12) increases, we are considering regions further and further away from Syria (e.g., region 2 is Anatolia, region 3 is Hungary-Croatia, etc.). Since the time elapsed for the Neolithic front to reach a region tends to be larger the further away it is from Syria, there was more time for interbreeding between farmers and hunter-gatherers. This is why the percentage of haplogroup K (vertical axis in Fig. S12) tends to diminish with increasing distance (recall that hunter-gatherers lack haplogroup K).

We note in Fig. S12 (i.e., Fig. 3 in the main paper) that the tendency of decreasing percentage of haplogroup K with increasing distance from Syria is not always satisfied (there is a minimum in Sweden, region 11). The explanation of this subtle point is the following. As it is well-known, the Neolithic spread from Syria to Anatolia, then to Greece, and from there it followed two different routes. One was a Mediterranean route to Italy, France, Spain and Portugal. The other was a central/northern European route to Croatia, Germany, Denmark and Sweden[46,66]. In order to see how this explains the minimum in Fig. S12, consider first a Neolithic front propagating along a coast. In this case, population dispersal can reach locations up to 150 km away (Text S6), measured in a straight line and across the sea (Materials and Methods in the main paper and Text S5). Now consider a Neolithic front propagating inland. In this case, dispersal is driven by jumps of about 50 km per generation (Materials and Methods and Text S5). Therefore, in order for the Neolithic front to travel a given distance, a *coastal* propagation obviously implies fewer jumps, i.e., fewer generations, and less time for interbreeding with hunter-gatherers than an *inland* propagation. Thus we should expect that a *coastal* propagation will lead, at a given distance, to a lower decrease of the percentage of haplogroup K (%K) than an *inland* propagation.

In Fig. S15 we have plotted the results of the simulations (for $\eta = 0.02$) for the two routes mentioned above (i.e., the Mediterranean and the central/northern European ones) separately. Up to Greece, both routes are the same and thus lead to the same %K as a function of distance. However, after Greece the Mediterranean route is mostly coastal (to France, Spain and Portugal), in sharp contrast with the central/northern European route, which is mostly inland (to Germany, Denmark and Sweden). Thus the %K of the central/northern European route becomes smaller than that of the Mediterranean route, for the reasons argued in the previous paragraph (see the slope change in the central/northern European route after its coastal spread ends up in Trieste in Fig. S15).

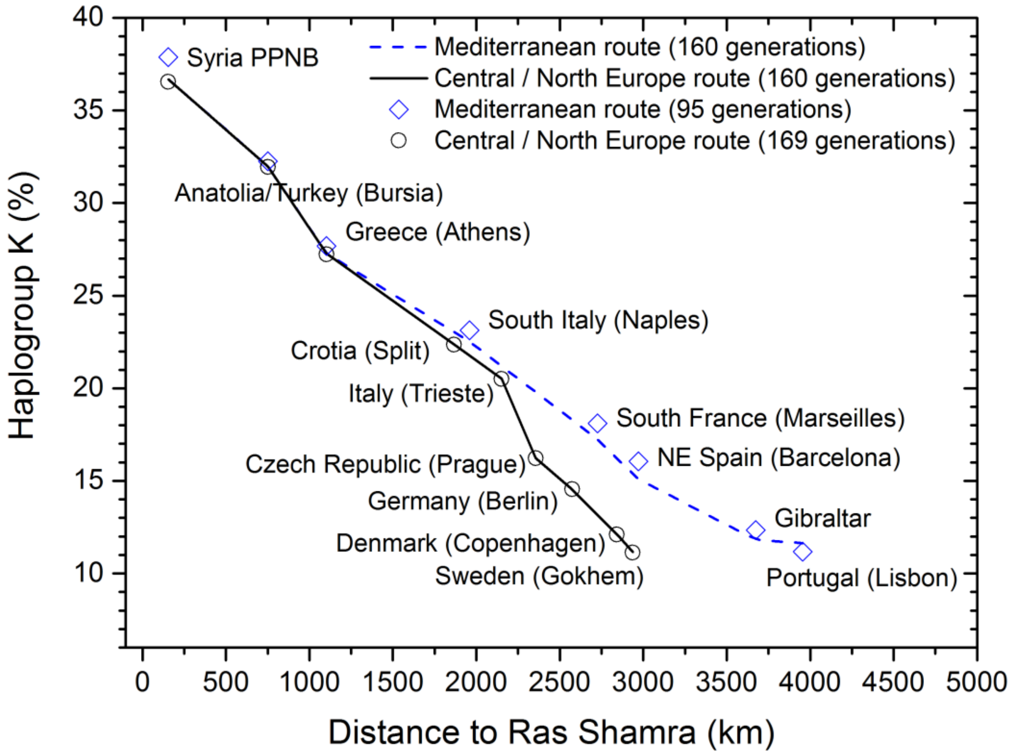

Figure S15. Results of the simulations for $\eta = 0.02$ along two spread routes. This figure is similar to, e.g., Fig. S12 (i.e., Fig. 3 in the main paper), but instead of considering the regions for which the DNA of ancient farmers has been determined, here we consider several locations on the two main routes along which the Neolithic spread, namely the Mediterranean route (dashed blue line) and the central/northern European route (solid black line). Another difference with Fig. S12 is that both lines in this figure have been obtained from our simulations at a single time, namely 3,113 yr BCE (i.e., 160 generations after the departure of the wave of advance from Ras Shamra, Syria). We have chosen this time so that the Neolithic wave of advance has reached all of Europe. However, in order to make sure that this figure can be used to understand the minimum in Fig. S12 (in spite of having used a value of time different from those used in Fig. S12), we also include the following results at other times. Blue rhombuses are results at locations on the Mediterranean route obtained at the most recent genetic date on that route, i.e. at the time of the genetic data of Portugal in Fig. S12 (5,184 yr BCE or 95 generations). Empty circles are the results at locations on the central/northern European route obtained at the most recent genetic date on that route, i.e. at the time of the genetic data of Sweden in Fig. S12 (2,802 yr BCE or 169 generations). We can see that considering different times leads to almost the same results, so the explanation of the minimum in Fig. S12 remains valid.

Now that we have understood the shape of the curve for each route (Fig. S15), we can explain the minimum in Fig. S12, as follows. If in Fig. S15 we joined the three points Germany-Sweden-NE Spain, we would obtain a minimum. This is precisely the minimum in Fig. S12 (where regions 4-5 are again Germany, region 11 is Sweden, and region 6 is NE Spain). Thus, the minimum in Fig. S12 is due to the existence of two propagation routes for the European Neolithic. These are the Mediterranean and the central/northern European routes, which are respectively (for large distances) a coastal route (with high %Ks) and an inland route (with lower %Ks), as seen in Fig. S15. Hence, the minimum in Fig. S12 is a purely geographical effect, due to the presence of the Mediterranean Sea. Below we will check this last point in another way (namely, by showing that simulations without sea display no minimum). However, before doing so, it is important to consider several related issues.

Genetic data from modern populations display distinct clines along the Mediterranean and central/northern European directions, and it has been suggested that this difference may be due to the respective routes of Neolithic dispersal[67]. Unfortunately, ancient mtDNA *data* are not yet numerous enough to distinguish whether both routes led to distinct ancient clines of haplogroup K or not. However, the presence of the minimum in Sweden, both according to the model *simulations* and to the data available (squares and circle in Fig. S12, i.e. Fig. 3 in the main paper), strongly suggests this possibility (see Fig. S15). Nevertheless, we cannot yet plot both *observed* clines (in contrast to the *simulated* ones in Fig. S15) due to the paucity of aDNA data available at present. Indeed, in Italy there are no mtDNA data from ancient farmers yet. In Greece, the mtDNA of only one early Neolithic individual is known[41]. In many other regions, there are data only from a small number of individuals (Fig. 2).

In Fig. S15, at the beginning of the spread, e.g. in Syria, the %K of the rhombuses is higher than that of the circles. This is reasonable, because rhombuses correspond to the %K at an earlier time than circles, thus less interbreeding with HGs has taken place. Note that this decrease (from the rhombus to the circle) is lower in Anatolia or Greece than in Syria, because populations of farmers with higher %K than the local frequency arrive to Anatolia and Greece but not to Syria (the origin of the expansion). On the other hand, Portugal is the only region where the %K increases with time (rhombus and dotted line in Fig. S15), because populations with higher %K arrive from the North, the South and the East into Portugal (see Fig. S10b).

Sweden is the latest region in Europe where the Neolithic arrived, and therefore the region with most recent DNA data in Fig. S12. Since more time implies more interbreeding with hunter-gatherers, and therefore a larger decrease in the %K, we could expect that the presence of the minimum in Sweden in Fig. S12 is due to the fact that we have plotted the %K as a function of distance, not of time. In order to check this point, in Fig. S16 we plot the %K as a function of time (not of distance as in Fig. S12). We observe that a minimum still appears. However, now the minimum does not correspond to Sweden (as in Fig. S12) but to Portugal (Fig. S16).

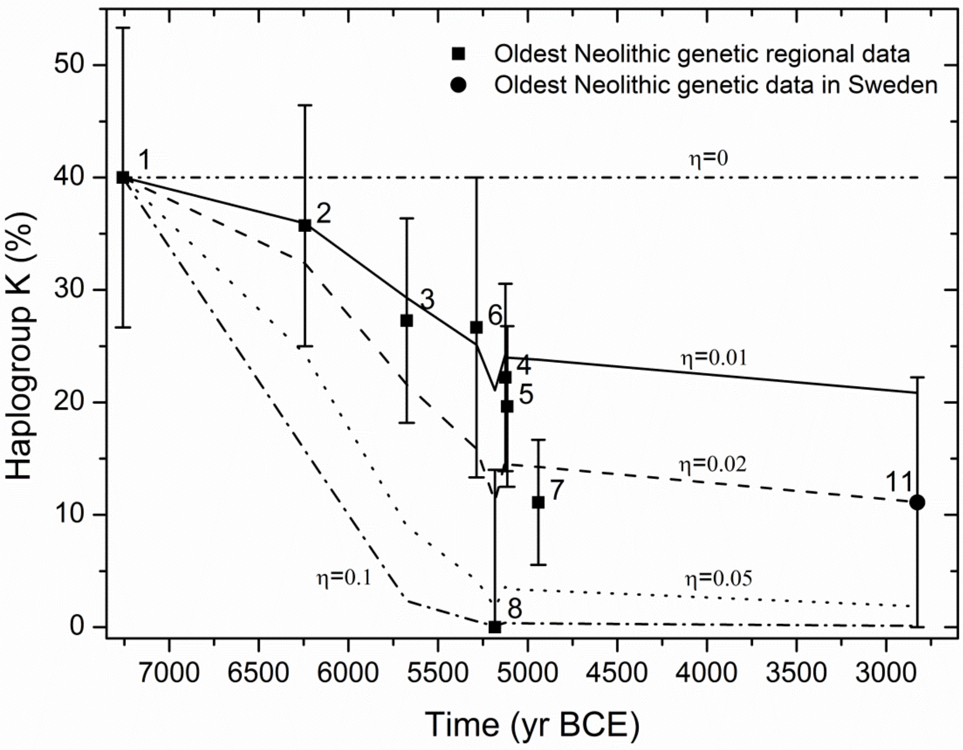

Figure S16. Percentage of mtDNA haplogroup K, *as a function of time*. The data points correspond to the same 9 regional cultures which have been plotted *as a function of distance* in Fig. S12, namely: 1 Syria PPNB, 2 Anatolia, 3 Hungary-Croatia Starčevo, 4 Eastern Germany LBK, 5 Western Germany LBK, 6 North-Eastern Spain Cardial, 7 Spain Navarre, 8 Portugal coastal Early Neolithic and 11 Sweden. The error bars are the 80% CL for the %K (i.e., the same as in, e.g., Fig. S12 or Fig. 3 in the main paper). The lines join the results of the simulations for different values of the cultural diffusion intensity η. Note that, in contrast to Fig. S12, region 11 (Sweden) appears at the right-hand side, and region 8 (Portugal) in the middle of the plot, where the minimum is now located.

We can explain the minimum in Fig. S16, again in terms of the Mediterranean and central/northern European routes, as follows. In Fig. S17 we plot the results of the simulations (for $\eta = 0.02$) for the two routes separately but as a function of time (not of distance as in Fig. S15). Similarly to our explanation above of the minimum in Fig. S12 (from the two routes in Fig. S15), we note that a minimum would appear in Portugal (region 8) in Fig. S17 if we joined regions 6-8-4/5. This is precisely the reason why we now see a minimum in Portugal (region 8) in Fig. S16 (rather than in Sweden as in Fig. S12).

The fact that the minimum appears in Portugal if time is the horizontal axis (Figs. S16-S17) whereas it appears in Sweden if distance is the horizontal axis (Fig. S12) can be understood as follows. Note that the value of the vertical axis (simulated %K) for each regional culture is the same in Figs. S16-S17 as for the dashed line in Fig. S12. Regional cultures 4, 5 (Germany) and 11 (Sweden) appear to the left of culture 8 (Portugal) in Fig. S12 because their distances are lower (so the minimum is in Sweden). However, they appear to the right of culture 8 (Portugal) in Figs. S16-S17 because their dates are later (so the minimum is in Portugal), simply because the average dates of the ancient individuals whose mtDNA haplogroup is known are more recent for cultures 4, 5 and 11 than for 8.

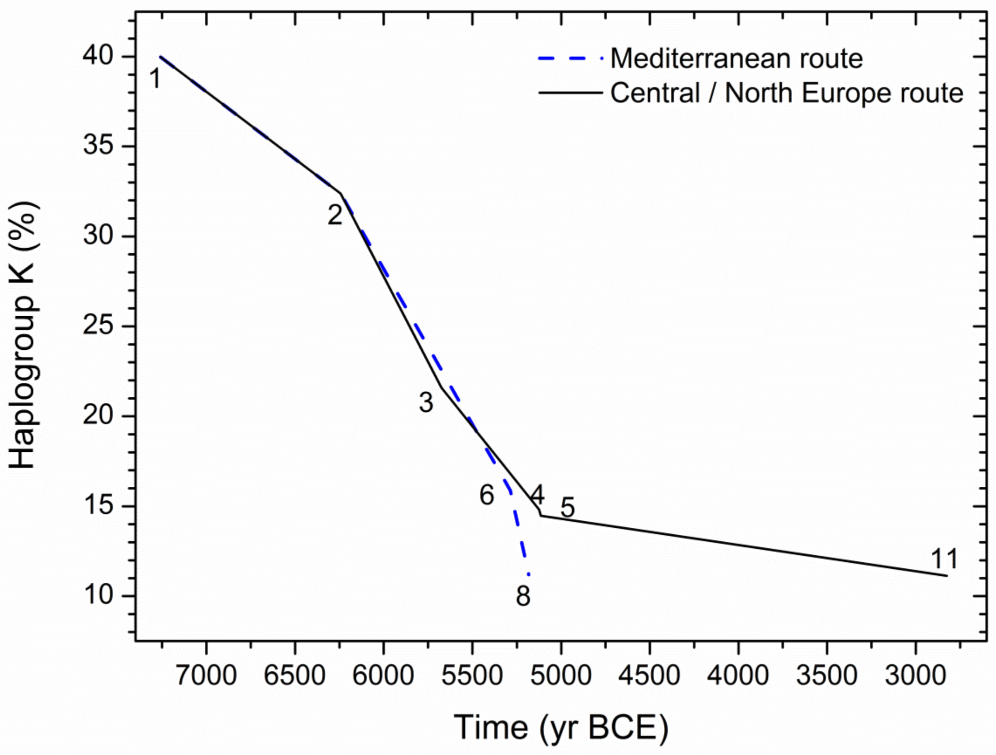

Figure S17. Results of the simulations for $\eta = 0.02$, as a function of time, for the regional cultures in Fig. S16 located on the Mediterranean route (dashed blue line) and the central/northern European route (solid black line). In contrast to Fig. S15, here we cannot consider a single value of time (because here the horizontal axis is time, not distance). Thus we consider the same regions and their values of time as in Fig. S16 (the value of time in each region being equal to the average date of the individuals whose mtDNA is known). A minimum would appear in Portugal (region 8) if we joined regions 6-8-4/5. These are precisely the regions where the minimum also appears in Fig. S16. This explains the minimum in Fig. S16.

Finally, we checked in another way that the minimum of the percentage of haplogroup K is indeed due to the geography of Europe. We simulated the spread of the Neolithic and its genetic dynamics using, instead of a map in Europe, a homogeneous space, i.e., a grid with only land nodes (without any seas neither mountains), so that all individuals that change their residence move 50 km (this simulation can be performed by modifying only the grid and initial conditions in Program S1, but for convenience we have made all of the necessary files available at the journal web as Program S4, or at http://copernic.udg.es/QuimFort/2017_08_07r__Program_S4.zip). We run our simulations on a grid of the same size as the geographically realistic grid (180x102 cells of 50kmx50km each). For simplicity, we have set the origin of the spread at the same node and with the same initial genetic conditions as in Fig. S12 (for $\eta = 0.02$) and Figs. S15-S17, namely, the node containing Ras Shamra, which has coordinates (112, 31) and initially (8,233 cal yr BCE) a percentage of farmers with haplogroup K equal to 42.2%K, which is the percentage needed in the previous simulations (in real geography) so that the 40%K in 1 Syria is correctly predicted at 7,258 cal yr BCE when $\eta = 0.02$ (see Figs S12 and S16-S17). In Fig. S18 we show the results of our simulations for $\eta = 0.02$ at the same cells used in the previous figures (e.g. Fig. S12), which we have labelled accordingly. However, since we are now dealing with homogeneous space, the results do not really correspond to the regional cultures in the previous figures (e.g., “1 Syria PPNB” or “11 Sweden”), but to points located at the same radial distance from the origin as them, for which we have computed the %K at 200, 500 and 900 generations after the beginning of the spread (Fig S18). For clarity we mention that in the node corresponding to the average location of region '1. Syria PPNB', i.e. node (115, 32), we find at 7,258 cal yr BCE a percentage equal to exactly 39.98% in real geographies (Fig. S12 for $\eta = 0.02$ and Figs. S16-S17) and 39.91% in homogeneous space (i.e., about 40% in both cases). However, in Fig S18 (homogeneous space) we obtain for the upper line about 36% (rather than 40%) because this result is after 200 generations (whereas the percentage 40% is obtained at 7,258 cal yr BCE, i.e. 30 generations after 8,233 cal yr BCE). For a Neolithic wave spreading in homogeneous space, we simply expect that the percentage of haplogroup K will diminish with increasing distance, and that this cline will gradually disappear as time passes (both features being due to interbreeding). This is precisely what can be observed from our simulations in Fig. S18, but most importantly, in contrast with Fig. S12, there is no local minimum in Fig. S18. Thus the minimum in, e.g., Fig. S12 indeed arises due to the presence of the Mediterranean sea in Europe, which leads to the existence of two expansion routes with differentiated dispersal behavior, namely the central/northern European route (which is mainly inland and has thus jumps of 50 km per generation) and the Mediterranean one (which is mainly coastal and has thus jumps of up to 150 km per generation).

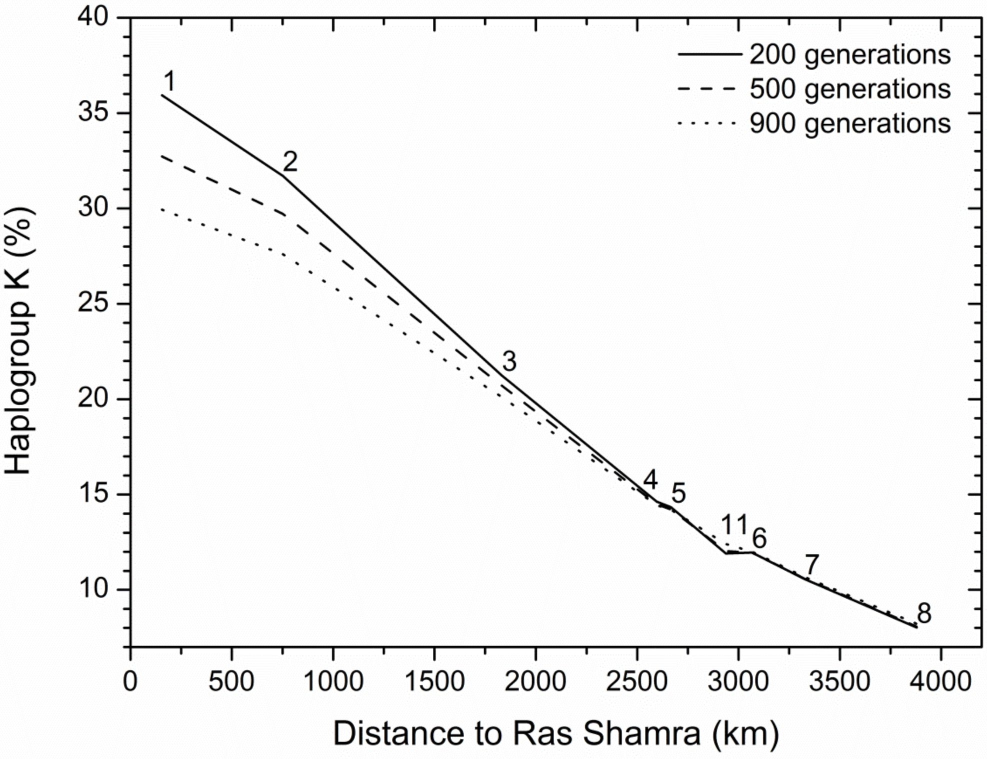

Figure S18. This figure shows the results of the simulations (for $\eta = 0.02$) without seas neither mountains in the simulation grid. We have set the start of the spread at the same cell and initial genetic conditions as in Figs. S12 and S15-S17, and the simulated results plotted correspond to the same cells as the cultural regions in Figs S12-S14 and S16-S17 (labelled accordingly in the figure) but at 200, 500 and 900 generations after the origin of the spread. As expected, the %K decreases with increasing distance from the origin of the spread, and the cline is gradually erased with time (1 generation=32 yr [51]). In contrast to the line in Fig. S12 for $\eta = 0.02$, no minimum appears here (homogeneous space). This confirms that the minimum in, e.g., Fig. S12 (which is the same as Fig. 3 in the main paper) is a purely geographical effect, due to the existence of the Mediterranean sea.

Text S9. Horizontal/oblique transmission

All models in the main paper and other sections in this Supplementary Information use the equations of vertical transmission, i.e. interbreeding between farmers and hunter-gatherers. In this section we show that the conclusions would not change if we considered, instead, acculturation, i.e. learning of agriculture by hunter-gatherers from farmers of the same generation (horizontal transmission) and/or the previous one (oblique transmission).

Vertical transmission leads to the following new population numbers (in each spatial cell) after one generation (see equations (S17)-(S19)),

$$P_{HG}(x,y,t+1) = R_{0,HG}[P_{HG}(x,y,t) - couples\ HX - couples\ HN], \tag{S20}$$

$$P_N(x,y,t+1) = R_{0,F}\ P_N(x,y,t)\ , \tag{S21}$$

$$P_X(x,y,t+1) = R_{0,F}[P_X(x,y,t) + couples\ HX + couples\ HN], \tag{S22}$$

where, for the cell considered, P_{HG} is the number of hunter-gatherers, P_N is the number of farmers who have haplogroup K, and P_X is the number of farmers who do not have haplogroup K. The numbers of mixed couples are given by equations (S5)-(S6), namely

$$couples\ HN = \eta \frac{P_{HG}(x,y,t) \cdot P_N(x,y,t)}{P_{HG}(x,y,t) + P_F(x,y,t)}, \tag{S23}$$

$$couples\ HX = \eta \frac{P_{HG}(x,y,t) \cdot P_X(x,y,t)}{P_{HG}(x,y,t) + P_F(x,y,t)}, \tag{S24}$$

and the total number of farmers in the spatial cell considered is $P_F(x,y,t) = P_N(x,y,t) + P_X(x,y,t)$.

We can interpret the meaning of η by noting that for pioneering, low-density populations of farmers ($P_N \approx 0$, $P_X \approx 0$ and thus $P_F \approx 0$), equations (S21)-(S24) for the special case $R_{0,F} = 1$ (no net reproduction) lead to $P_F(x,y,t+1) - P_F(x,y,t) \approx \eta P_F(x,y,t)$, so that η can be interpreted as the relative increase in the number of farmers per generation due to interbreeding with HGs (i.e., the proportion of farmers that take part in vertical cultural transmission).

For horizontal/oblique transmission[53], the first three equations are valid replacing the number of couples by the number of hunter-gatherers who learn farming (which we call converts and do not have haplogroup K, i.e. they belong to population X) from each population of farmers (X and N), i.e.

$$P_{HG}(x,y,t+1) = R_{0,HG}[P_{HG}(x,y,t) - converts\ HX - converts\ HN], \tag{S25}$$

$$P_N(x,y,t+1) = R_{0,F}\ P_N(x,y,t)\ , \tag{S26}$$

$$P_X(x,y,t+1) = R_{0,F}[P_X(x,y,t) + converts\ HX + converts\ HN], \tag{S27}$$

where[53]

$$converts\ HN = f \frac{P_{HG}(x,y,t) \cdot P_N(x,y,t)}{\gamma P_{HG}(x,y,t) + P_F(x,y,t)}, \tag{S28}$$

$$converts\ HX = f \frac{P_{HG}(x,y,t) \cdot P_X(x,y,t)}{\gamma P_{HG}(x,y,t) + P_F(x,y,t)}. \tag{S29}$$

Analogously to the paragraph below Eq. (S24), we can interpret the meaning of C by noting that for pioneering populations of farmers ($P_N \approx 0$, $P_X \approx 0$ and thus $P_F \approx 0$), equations (S26)-(S29) for the special case $R_{0,F} = 1$ (no net reproduction) lead to $P_F(x, y, t + 1) - P_F(x, y, t) \approx CP_F(x, y, t)$, so that $C \equiv f/\gamma$ can be interpreted as the relative increase in the number of farmers per generation due to acculturation with HGs (which is the same, if $C < 1$, as the proportion of farmers that take part in horizontal/oblique cultural transmission)[53].

In the simple case $\gamma = 1$ (which corresponds to random copying of behavior between individuals[53]), it is easy to see that $0 \leq f \leq 1$ (otherwise, $P_{HG}(x, y, t + 1)$ could become negative for $P_{HG} << P_F$). Then equations (S25)-(S29) for horizontal/oblique transmission are the same as equations (S20)-(S24) for vertical transmission, with η replaced by f. Recall also that for vertical transmission $0 \leq \eta \leq 1$ [57]. Thus, the same model as in the main paper can be used for horizontal/oblique transmission, instead of vertical transmission. Obviously, for horizontal/oblique transmission the conclusion (from Fig. 3, or Fig. S12) would be that $f = 0.02$ (instead of $\eta = 0.02$), i.e. that about 2% of new farmers join the pioneering farming populations per generation due to acculturation of hunter-gatherers (instead of due to interbreeding with hunter-gatherers) or, equivalently, that about 2% of farmers teach agriculture to a hunter-gatherer (instead of mating a hunter-gatherers).

A more general case is to consider both horizontal/oblique transmission (acculturation) and vertical transmission (interbreeding). In such an instance, the corresponding equations (as given above) should be applied sequentially in the simulations, in general with different values for parameter f (horizontal/oblique transmission) and η (vertical transmission). Accordingly, the equations are a bit more complicated, because vertical transmission makes the frequencies of parent and children different, so it must be taken into account explicitly that the teachers belong to the parental generation in oblique transmission but not in horizontal transmission (compare Eqs. (3.4.1) to (3.1.3) in Ref.[59]). We do not perform such simulations, for the following reason. We have estimated the value of η (namely $\eta \approx 0.02$) in Fig. 3 in the main paper, by assuming only vertical transmission. Alternatively, if we considered only horizontal/oblique transmission, we would estimate the same value for f (i.e., $f \approx 0.02$). But if we considered a model with both kinds of transmission, we would have at least two independent parameters (η and f), and we cannot estimate both of them univocally from the genetic data available (i.e., from the error bars in Fig. 3 in the main paper). However, we next show that the conclusions in the main paper would not change under such more complicated models. Clearly, values $\eta \approx 0.02$ and $f \approx 0.02$ or higher would yield more cultural transmission than the case considered in the main paper (Fig. 3), i.e. $\eta \approx 0.02$ and $f = 0$. Therefore, for values $\eta \approx 0.02$ and $f \approx 0.02$ or higher, obviously the simulated cline will be too steep to be consistent with the genetic data (error bars in Fig. 3). Thus, we can assure that more complicated models (i.e., with both vertical and horizontal/oblique transmission) will be consistent with the genetic data only if $\eta < 0.02$ and $f < 0.02$ (more precisely, we should expect e.g. $\eta + f \leq 0.02$). These values are very small compared to the maximum possible ones (namely $\eta = 1$ and $f = 1$). Noting that, in regions where the first famers arrived ($P_N \approx 0$ and $P_X \approx 0$), the equations above simplify to $couples\ HN \approx \eta P_N$, $couples\ HX \approx \eta P_X$ (for vertical transmission) and $converts\ HN \approx f P_N$, $converts\ HN \approx f P_X$ (for horizontal/oblique transmission), we can interpret the result above ($\eta + f < 0.02$) by stating that less than 2% of farmers took part in cultural transmission,

either by mating with hunter-gatherers or by teaching agriculture to them. Thus, about 98% of the population did not take part in cultural transmission. In this sense, cultural diffusion was of little importance. Therefore, the main conclusion of our work remains the same, regardless that we consider vertical, horizontal, oblique, or any combination of these three kinds of cultural transmission.

Text S10. Calculation of the error bars of the percentages of haplogroup K

For each sample (e.g. Syria PPNB, Anatolia, Hungary-Croatia Starčevo, etc.), we calculated the 80% confidence-level (CL) range of its percentage of haplogroup K (hereafter called %K), which we represent as error bars in Figs. 2-3, by bootstrap case resampling. In order to do so, we drew 10,000 random resamples from each original sample with replacement. Each resample had the same number of individuals as the original sample (e.g., 15 individuals for Syria PPNB, 28 for Anatolia, etc.). For these 10,000 resamples, we computed a histogram with the number of resamples versus their %K. Then, with 80% CL, the error bar is limited by the 10% and 90% quartiles of this distribution (i.e. the values of the %K below which there are 10% and 90% of the histogram resamples, respectively). We performed these calculations using Mathematica, and checked them using Excel.

However, this bootstrap method cannot be applied to the case of populations with 0%K (e.g., Portugal coastal Early Neolithic), simply because then there are no individuals with haplogroup K, so all bootstrap samples have 0 individuals with haplogroup K. This would yield a vanishing error bar for the estimation 0%K, which is not reasonable, for the following reason. For example, for the sites in the sample called 'Portugal coastal Early Neolithic' there are only 10 individuals. None of them has haplogroup K, so its frequency is obviously 0%. However, if we had e.g. 100 individuals, and none of them had haplogroup K, its frequency would again be 0% but with more certainty, i.e., the error bar should be narrower. Thus, assigning a vanishing error bar to samples with 0% of a haplogroup is not justified. In order to deal with such samples, we could begin by introducing reasonable assumptions, e.g. by adding noise to the data[68-70]. However, such approaches would require hypotheses (on the kind of noise, its parameter values, etc.)[68-70]. Clearly, it would be better to find a solution without introducing such assumptions. With this aim, we devised the following method.

Although our method is general, for clarity let us consider a specific sample we are interested in, e.g. 'Portugal coastal Early Neolithic'. As mentioned above, in this sample there are only 10 individuals and none of them carried haplotypes from haplogroup K, so the K frequency is 0%. For the sake of simplicity, as a first step, imagine 11 possible populations (each of them composed of a very large number of individuals), with percentages of individuals with haplogroups different than K (which we call "0" individuals) equal to 100%, 90%, 80%, ..., 20%, 10% and 0%. We call those populations P_{100}, P_{90}, ..., P_{10}, P_0, respectively (note that they have %s of the K haplogroup equal to 0%, 10%, ..., 90% and 100%, respectively). Imagine that we choose at random 1 of these 11 populations, next we choose 10 individuals at random from it, and it turns out that all of them are "0" individuals (i.e., none of them has haplogroup K, as in the case of 'Portugal coastal Early Neolithic'). In such a situation, obviously it is more likely that we have chosen the population P_{100} than P_{90}, it is also more likely that we have chosen the

population P_{90} than P_{80}, etc. But what are the exact probabilities that we have chosen each population? The probability of population P_{100} for the situation considered (i.e., that in which all 10 individuals are "0") is

$$p(\mathrm{P}_{100}\,|\,0000000000) = \frac{p(\mathrm{P}_{100} \cap 0000000000)}{p(0000000000)}, \quad \text{(S30)}$$

where the symbol ∩ denotes intersection, i.e. co-occurrence of the two events, and

$$p(\mathrm{P}_{100} \cap\ 0000000000) = \frac{number\ of\ cases\ \mathrm{P}_{100}\ and\ 0000000000}{number\ of\ total\ cases}, \quad \text{(S31)}$$

$$p(0000000000) = \frac{number\ of\ cases\ 0000000000}{number\ of\ total\ cases}\quad , \quad \text{(S32)}$$

and the number of total cases includes all 11 possible populations and all possible outcomes besides 0000000000 (e.g., 1000000000, 010000000, 1100000000, etc.). Similarly for the other populations,

$$p(\mathrm{P}_{90}\,|\,0000000000) = \frac{p(\mathrm{P}_{90} \cap 0000000000)}{p(0000000000)}, \quad \text{(S33)}$$

$$p(\mathrm{P}_{80}\,|\,0000000000) = \frac{p(\mathrm{P}_{80} \cap 0000000000)}{p(0000000000)}, \quad \text{(S34)}$$

etc. Clearly, since they refer to two independent events, the numerators in equations (S30), (S33), (S34), etc. are equal to the probability that we have chosen the considered population P_i (namely $\frac{1}{11}$, because it has been chosen at random) times the probability that, if we have chosen this population, we have also chosen a sample in which all 10 individuals are "0". Thus

$$p(\mathrm{P}_{100} \cap\ 0000000000) = \frac{1}{11}1 = \frac{1}{11}, \quad \text{(S35)}$$

$$p(\mathrm{P}_{90} \cap\ 0000000000) = \frac{1}{11}(0.9^{10}) = \frac{0.9^{10}}{11}, \quad \text{(S36)}$$

$$p(\mathrm{P}_{80} \cap\ 0000000000) = \frac{1}{11}(0.8^{10}) = \frac{0.8^{10}}{11}, \quad \text{(S37)}$$

etc. By adding up these values, equation (S32) can be written as

$$p(0000000000) = \frac{1}{11}(1 + 0.9^{10} + 0.8^{10} + \cdots + 0.1^{10} + 0^{10}), \quad \text{(S38)}$$

and we find the final result for population P_{100} from equations (S30), (S35) and (S38) as

$$p(\mathrm{P}_{100}\,|\,0000000000) = \frac{1}{1+0.9^{10}+0.8^{10}+\cdots+0.1^{10}} = 0.6705. \quad \text{(S39)}$$

Similarly we find, for the other populations,

$$p(\mathrm{P}_{90}\,|\,0000000000) = \frac{0.9^{10}}{1+0.9^{10}+0.8^{10}+\cdots+0.1^{10}} = 0.2338, \quad \text{(S40)}$$

$$p(\mathrm{P}_{80}\,|\,0000000000) = \frac{0.8^{10}}{1+0.9^{10}+0.8^{10}+\cdots+0.1^{10}} = 0.0720, \tag{S41}$$

etc. As expected, the probability is highest for population P_{100} (i.e., for 0%K). The important point is that, in contrast to the bootstrap method with case resampling (which would predict that P_{100} is the only possible population), we have computed non-vanishing probabilities for the other populations (and they decrease with increasing %K, also as expected). Moreover, by adding equations (S39) and (S40), we find that

$$p(\mathrm{P}_{100}\,|\,0000000000) + p(\mathrm{P}_{90}\,|\,0000000000) = 0.9043, \tag{S42}$$

from which we can state that there is a probability of 90.43% that our sample comes from a population with a percentage of "0" individuals between 100% and 90%. In other words, we have found, with 90.43% confidence level, that our sample comes from a population in which the percentage of haplogroup K is in the range 0%-10%K. Note, however, that in this first computation (i.e., using 11 possible populations) there is a lot of uncertainty, because the closest possible result that we can possibly estimate would be obtained by adding $p(\mathrm{P}_{80}\,|\,0000000000)$ to equation (S42) and then, the range of the percentage of "0" individuals would be between 100% and 80%, i.e., the upper limit of of the % of haplogroup K would be 20%K, rather than 10%K as above. Thus, it is safe to accept that there is an error of up to 10% in the estimation of percentages using 11 populations (another way to see this is simply to note that our 11 possible populations are separated by increases of 10%K). Therefore, our conclusion should be that, with 90.43% confidence level, the original population had a frequency in the range 0%-20%K.

Note also that, for the CL we are interested in, namely 80% (because this is the range used in the main paper), this first calculation does not lead to a precise range of the %K, because we can only estimate such a range with a 67.05% CL (using equation (S39)), with a 90.43% CL (using equation (S42)), etc. We next show that we can solve this problem by considering a larger number of possible populations.

Secondly, we repeat the previous procedure with 101 populations (instead of 11 as above), with percentages of "0" individuals equal to 100%, 99%, 98%, ..., 2%, 1% and 0 %. Thirdly, we repeat the same approach with 1,001 populations (with percentages 100%, 99.9%, ..., 0.1% and 0%). And fourthly, we do the same with 10,001 populations (with percentages 100%, 99.99%, ..., 0.01% and 0%). The results (obtained using the Mathematica computer program) are shown in Table S2.

As expected, the higher the number of populations, the lower the error of the estimated %K, and we can choose a CL closer and closer to 80%. Note also that each error bar is within the previous one, as it should (because an estimation with more populations, as designed above, is obviously more precise). From the last column we can safely conclude, with 80% CL, that the percentage of haplogroup K in a population for which we have measured a sample of 10 "0" individuals (i.e., in which none of the 10 individuals has the haplogroup K), is within the range 0%-14%. Thus we have applied the error bar 0-14%K to the sample 'Portugal coastal Early Neolithic' in the main paper.

Number of possible populations	upper limit of the % of haplogroup K	error bar of the % of haplogroup K	confidence level (CL)
11	(10±10)%K	0%-20%K	90.40%
101	(13±1)%K	0%-14%K	80.80%
1,001	(13.6±0.1)%K	0%-13.7%K	80.21%
10,001	(13.61±0.01)%K	0%-13.62%K	80.02%

Table S2. Error bar estimation for the regional culture 'Portugal coastal Early Neolithic' (10 individuals and 0%K).

Our method could be also applied to cases in which the haplogroup percentage is different from 0%, but calculations would be more tedious (because it is less straightforward to compute, e.g., the probability of 4 "0"s and 6 "1"s than to compute that of 10 "0"s). If the haplogroup percentage is different from 0%, we prefer to use the bootstrap approach because it is a reasonable method which makes it possible to compare directly to the error bars estimated by other authors (e.g., references [1,37]).

Besides the sample 'Portugal coastal Early Neolithic' (which has 10 individuals, none of them with haplogroup K), in Fig. 2 (main paper) there is another sample with 0%K, namely 'Romanian Late-Middle Neolithic' (which has 9 individuals, none of them with haplogroup K). Repeating the same procedure as above for 9 (instead of 10) individuals, the result is that, with 80% CL, the percentage of haplogroup K in the original population is 0-15%K. The upper bound is higher than for 10 individuals, as it should, because with fewer individuals in a sample, inference about properties of the complete population (from which the sample has been drawn) is obviously more uncertain.

Text S11. A more complicated simulation model

In our main paper and in Text S5, the equations used to compute cultural transmission assume that both male and female hunter-gatherers are equally liable to form mixed couples with Neolithic individuals. However, ethnographic studies show that, in similar situations, mating takes place mostly between female hunter-gatherers and male farmers (see, e.g., reference [56]). If only female hunter-gatherers can mate with farmers, then none of the HN couples will contribute haplogroup K to the Neolithic gene pool, because mtDNA is inherited only from the mother. Note, however, that taking this point into account will not modify the genetic contribution of HX couples (because none of the parents has haplogroup K) neither NX couples (because both N and X are farmers, so the female can be either of them), neither of course HH, NN nor XX couples. On the other hand, the maximum possible number of both HN and HX couples will be smaller (by 50%) than in the model in the main paper and Text S5, because only female HGs can take part in them. Therefore, some genetic impact could in principle be expected if using this more realistic approach. Here we take this point into account, by means of an alternative cultural transmission scheme described below. We find, however, that the change in the results is in fact minimal, so the conclusions in the main paper do not change. We will also suggest some intuitive explanations of why this effect is so small.

In this model (Program S2, available at the journal web or at http://copernic.udg.es/QuimFort/2017_08_07r__Program_S2.zip) only part of the hunter-gatherer population (the females) can mate with farmers, so we have to consider separate sub-populations for men and women. Let $M_{HG}(x,y,t)$ and $W_{HG}(x,y,t)$ stand for the number of hunter-gatherer men and women, respectively, present in a cell after dispersal (step 1 in the main paper, Materials and Methods). Therefore, the total number of hunter-gatherers in the cell is $P_{HG}(x,y,t) = M_{HG}(x,y,t) + W_{HG}(x,y,t)$. Likewise, let $M_F(x,y,t)$ and $W_F(x,y,t)$ stand for the farmer sub-populations of men and women, which are in turn divided into $M_N(x,y,t)$ and $W_N(x,y,t)$ for the farmer population with haplogroup K present in the cell, and $M_X(x,y,t)$ and $W_X(x,y,t)$ farmers that do not have haplogroup K. In the computer code we assume that initially there is gender balance in all populations, i.e. that there is the same number of males and females, and that in the new generations there is also equal probability to be born male or female.

Cultural transmission

The cultural transmission process (step 2 in the main paper, Materials and Methods, and detailed in Text S5) is now replaced by the following.

Cross-matings between cultural groups. For cells with Mesolithic and Neolithic individuals, we first compute the mixed couples by taking into account that only hunter-gatherer women can mate into the farmer community. Let us first find, for example, the probability for a hunger-gatherer woman to mate with a farmer man who has haplogroup K. Under random mating (same tendency to mate with a hunter-gatherer man than with a farmer man), this probability would be simply the fraction of men with haplogroup K (relative to the whole male population in the cell). However, in general, this probability will be reduced by the interbreeding parameter η which, when $\eta < 1$, favors mating within the same population over mixed matings. Therefore, the probability for a hunger-gatherer woman to mate with a farmer man who has haplogroup K is given by[57]

$$\eta \frac{M_N}{M_N + M_X + M_{HG}}, \qquad \text{(S43)}$$

where $M_N + M_X + M_{HG}$ is the total male population in the cell. Multiplying this probability (S43) by the number of hunter-women, W_{HG}, we find the corresponding number of mixed couples

$$couples\ M_N W_{HG} = \eta \frac{W_{HG} \cdot M_N}{M_N + M_X + M_{HG}}. \qquad \text{(S44)}$$

Similarly, we find for the number of matings to farmer men who do not have the haplogroup K

$$couples\ M_X W_{HG} = \eta \frac{W_{HG} \cdot M_X}{M_N + M_X + M_{HG}}. \qquad \text{(S45)}$$

Note that equations (S44)-(S45) are similar to equations (1)-(2) in the main paper, so we are actually applying vertical cultural transmission, but only to the subgroups liable to form mixed couples.

Analogously to the model used in our main paper (equation (S7)-(S9)), we next compute the number of farmer men and hunter-gatherer women who do not take part in the mixed matings above,

$$M'_N = M_N - couples\ M_N W_{HG} \tag{S46}$$

$$M'_X = M_X - couples\ M_X W_{HG} \tag{S47}$$

$$W'_{HG} = W_{HG} - \ couples\ M_N W_{HG} - couples\ M_X W_{HG} \tag{S48}$$

Cross-matings between genetic groups of farmers. We now compute the number of couples between farmer individuals of different genetic groups (N and X). Since some farmer men have mated with hunter-gatherer women, we now have fewer farmer men than farmer women (remember that we initially had gender balance). We can find the probability for a farmer man to mate with a farmer women of the other genetic group. This will now just be the fraction of women of the other genetic group (relative to all farmer women). As argued above equation (3) in the main paper, there is no reason to assume any preference toward or against matings within the same genetic group, and therefore we can assume $\eta = 1$ (random mating). As a result, the number mixed genetic couples within the farmer community are given by

$$couples\ M_N W_X = \frac{M'_N \cdot W_X}{W_N + W_X}, \tag{S49}$$

$$couples\ M_X W_N = \frac{M'_X \cdot W_N}{W_N + W_X}, \tag{S50}$$

where $W_N + W_X = W_F$ is the total number of farmer women. Equations (S49)-(S50) are analogous to equation (S10) for the simpler model used in our main paper.

Matings within groups. Finally, the number of couples between farmers of the same genetic group is constrained by the number of unmated men (which are fewer in number than unmated women). In the same way, the number of couples between hunter-gatherers is constrained by the number of unmated women. Therefore,

$$couples\ M_N W_N = M'_N - couples\ M_N W_X\ , \tag{S51}$$

$$couples\ M_X W_X = M'_X - couples\ M_X W_N\ , \tag{S52}$$

$$couples\ M_{HG} W_{HG} = W'_{HG}. \tag{S53}$$

Note that, in contrast to the analogous equations in the simpler model applied in the main paper (equations (S11)-(S13)), here we do not need to divide by two because we are now dealing with men and women separately.

Reproduction

The following scheme replaces the reproduction step in the main paper (Materials and Methods) and Text S5. We apply the following rules. (i) Each couple will have $2R_{0,i}$ children, because $R_{0,i}$ is computed per individual and there are two individuals per mating. However, the net growth rate $R_{0,i}$ is different for farmers and HGs ($i = F, HG$). Applying that the children from cross matings between HG and F will be farmers[55,56], we use $R_{0,HG} = 1$ for matings in which both parents are HGs (assuming that the HG population is stationary), and $R_{0,F} = 2.45$ [54] for HN, HX, NN, XX and NX matings. (ii) Since mtDNA is inherited from the mother, all the children from each couple will become part of the same genetic group as the mother. (iii) We assume equal probability for the children being male or female, so 50% of the new population will be men and the other 50% women. Under these three rules, the number of men and women in the next generation is given by

$$M_{HG}(t+1) = W_{HG}(t+1) = R_{0,HG} \cdot couplesM_{HG}W_{HG}\,, \tag{S54}$$

$$M_N(t+1) = W_N(t+1) = R_{0,F}(couples\ M_N W_N + couples\ M_X W_N)\,, \tag{S55}$$

$$M_X(t+1) = W_X(t+1) = R_{0,F}(couples\ M_X W_X + couples\ M_N W_X + couplesM_X W_{HG} + couplesM_N W_{HG})\,. \tag{S56}$$

These equations are analogous to equations (S14)-(S16) for the simpler model applied in the main paper. Note that the couples *HN* appear in equation (S15) but not in equation (S55), because here all HGs in those matings are women and their mtDNA haplogroup is inherited by their children (so none of the latter will have haplogroup K and, therefore, never belong to population N but always to X). Finally, although this is not necessary to perform the simulations, using equation (S46)-(S48) into (S51)-(S53) and the results into (S54)-(S56) we can relate the population numbers at generation $t+1$ to those at the previous generation t,

$$M_{HG}(t+1) = W_{HG}(t+1) = R_{0,HG}\ (W_{HG}(t) - couplesM_N W_{HG} - couplesM_X W_{HG})\,, \tag{S57}$$

$$M_N(t+1) = W_N(t+1) = R_{0,F}\ (M_N(t) - couples\ M_N W_{HG} - couples\ M_N W_X + couples\ M_X W_N\ ), \tag{S58}$$

$$M_X(t+1) = W_X(t+1) = R_{0,F}\ (M_X(t) - couples\ M_X W_N + couples\ M_N W_X + couples\ M_N M_{HG})\,, \tag{S59}$$

which are analogous to equations (S17)-(S19) for the simpler model used in the main paper.

Simulation results

If we apply this new cultural transmission-reproduction scheme to the same initial conditions as in the main paper, we obtain basically the same results, as can be observed by comparing Fig. S19 below to Fig. S12 (i.e., Fig. 3 in the main paper). The absolute differences between the predicted fractions of individuals with haplogroup K are lower than 0.002. Therefore, although the scheme used in the main paper is more simplified, its results are close enough to those obtained here to validate the use of such an approximation. Also, because both models yield nearly the same results, the conclusions of the paper remain unchanged.

The fact that the refined model (this section) and the approximate one (main paper and Text S5) lead to much the same results does not seem very surprising, for the following reasons. It is true that in the more refined model (this section) only female hunter-gatherers (without haplogroup K) are incorporated into the farming populations (thus all of their children lack haplogroup K), whereas in the approximate model (main paper and Text S5) additional (male) hunter-gatherers are also incorporated. However, the children of the latter do not always have haplogroup K (they will have it if the mother belongs to group N, but not if she belongs to group X). Thus the *HN* matings that are not taken into account in the more refined model (this section) lead not only to children who have haplogroup K, but also to children who do not have in the approximate model (main paper and Text S5). Then it seems reasonable that the effect of this refinement on the percentage of haplogroup K is small. Moreover, the genetic contribution of most matings (i.e., HX, NX, HGHG, NN and XX) is unaffected by this refinement in the model. Another difference is that the maximum possible number of HN and HX matings is lower in the model in this section (because only women HGs and men farmers take part in them), but again all other matings (i.e., NX, HGHG, NN and XX) are unaffected and, moreover, in our simulations we have observed that the percentage of haplogroup K becomes almost constant many generations before all possible matings with HGs have taken place (i.e., before the local HGs extinguish in the model in the main paper and Text S5, or before the local HG women extinguish in the model in this section).

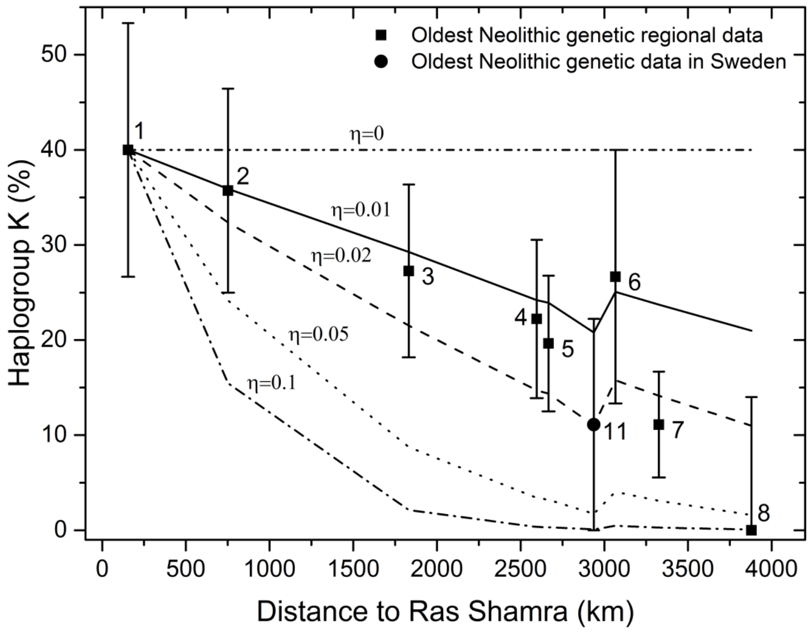

Figure S19. This figure is the same as Fig. S12 (i.e., Fig. 3 in the main paper), but applying the more refined model in Text S11. It shows the percentage of mtDNA haplogroup K present in the farmer population as a function of distance to Syria. Black squares correspond to the measured data. Lines correspond to the results of the simulations, using the model in Text S11, for several values of the interbreeding parameter η. The results that follow from this more precise model are almost the same as those from the model used in the main paper (compare this figure to Fig. S12, i.e. Fig. 3 in the main paper).

Text S12. Approximate, one-dimensional model

In this work we have concluded that a value of the interbreeding parameter η as low as $\eta = 0.02$ (which is very small, as compared to the maximum possible value $\eta = 1$ [57]) explains the observed cline of haplogroup K in aDNA data (main paper, Fig. 3). In order to perform a check to the validity of this new result, we conceived an approximate, simpler model as follows. The model in the main paper (detailed in Text S5), as well as the more elaborate one in Text S11 above, considers a two-dimensional (2D) grid, and distinguishes sea, mountain, coast and inland cells to simulate a real map of Europe. On this 2D grid, individuals are exchanged between cells via *sea* travels (up to 150 km, as implied by archaeological data; see Text S6) and also via *inland* travels (of 50 km, as implied by ethnographic data[52]). We reasoned that, since *sea* travels can be substantially longer than *inland* travels, a one-dimensional (1D) model (representing the Mediterranean coast) could be a simpler way to describe roughly the dynamics of the system. Although this is admittedly a simplification, and will obviously lead to less precise results, it seems reasonable to expect that it can be useful to check the main conclusion of our work (namely, that $\eta \ll 1$, as explained above).

In this one-dimensional model (Program S3, available at the journal web or at http://copernic.udg.es/QuimFort/2017_08_07r__Program_S3.zip) we assume a line of 150 nodes, each one separated 150 km from their two neighbors. This corresponds to a total of 22,350 km between the two extreme nodes (150 km multiplied by 149 jumps between nodes). As in the main model, initially only one node has Neolithic population (3,200 individuals) but no Mesolithic population, and all other nodes have no Neolithic population and 160 Mesolithic individuals per node (values obtained from ethnographic data[13] and the area of a cell in the main model). From the node with Neolithic individuals, the Neolithic population expands along the line (which corresponds to the Mediterranean coast) by performing, each generation, the steps of population dispersal, cultural transmission and reproduction. The latter two steps are treated here in the same way as in the main paper (detailed in Text S5). Dispersal, on the other hand, needs to be treated differently because of the unidimensionality of space.

Dispersal

The nodes in the 1D grid (this section) are equivalent to coastal nodes in the 2D grid (main paper and Text S5). The population present at a coastal cell in the 2D model can stay with a 38% probability (persistence)[52], or it can travel either inland or by sea (with the number of individuals taking each route depending on the number of sea neighbors). In general, in the 2D model we have three possibilities (we ignore the cases where a neighbor is a mountain cell), depending on the number of sea neighbors: (i) one sea neighbor implies that 25% of the population that travels (15.5% of the total population, computed as $(1/4)(1-p_e)$) moves by sea, (ii) two sea neighbors means that 50% of the traveling population (31% of the total population, computed as $(1/2)(1-p_e)$) will travel by sea, and (iii) three sea neighbors means that 75% of the traveling population (46.5% of the total population, computed as $(3/4)(1-p_e)$) will travel by sea.

In the approximate 1D model (this section), we are only considering sea travel. In contrast, in the 2D model (main paper and Text S5) we consider both sea and inland travel. For this reason, obviously in the 1D model if we allowed for all of the population that can travel (62% of the cell population) to migrate by sea, the speed of the front would largely overestimate the results obtained with the more realistic 2D model (which agree with the archaeological data, see Text S6). In addition, nodes in the 1D model are separated 150 km, so shorter jumps are not possible. In contrast, in the 2D model not all of the population that travels by sea moves 150 km away from the origin, because part of it moves to closer coastal locations. Therefore, for the 1D model to provide equivalent results, it is important that a lower fraction of the population travels by sea, so that the results are realistic. This implies that part of the population has to disappear from the system in the 1D model, in order to take care of the fraction of the population that travels inland (and, therefore, does not contribute to the coastal expansion) in the 2D model. Hence, in our 1D model a fraction $\alpha(1-p_e)$ of the population travels by sea, and a fraction $(1-\alpha)(1-p_e)$ of the population disappears. We find by trial and error a fair approximation to the value of α by setting the following constraint. For the 1D model to be a good approximation of what happens in a real geography, the arrival times for the 1D and the 2D models must be the same. We have chosen a coastal cell as a test origin for the 2D model, and a cell located 5,100 km away (distance measured along the coast) to calibrate the 1D model (5,100 km correspond to 34 jumps of 150 km each). As in Text S6, the arrival time of the Neolithic to a cell is recorded by the simulations as the time

when the population of farmers is 10% of its saturation value. In the 2D model, and with jumps of 150 km, a node located at 5,100 km is reached within 75 generations. With the 1D model, a node located 5,100 km away from the origin (i.e., 34 cells away) is reached within 52 generations if we assume that $(1/2)(1-p_e)$ individuals travel by sea, within 69 generations if $(1/3)(1-p_e)$ individuals travel by sea, and within 83 generations if we assume that $(1/4)(1-p_e)$ individuals travel by sea. This allows us to fine tune the best approach to a fraction of the population that must travel by sea in the 1D model to $0.3(1-p_e)$, which yields an arrival time within 75 generations, equivalent to the one measured in the 2D model.

Therefore, in the dispersion process of the 1D model, 38% of the population stays in the same node, and a fraction 0.3 of the remaining population ($0.3(1-p_e)$, i.e 18.6% of the total population) will travel by sea, half of them forward and the other half backward (similarly to the 2D model, where all possible destinations receive equal fraction of the sea travelling population). The rest of the population, as mentioned above, disappears from the system, representing the population that would travel inland (in the 2D model).

Simulation results

We now run the 1D model (Program S3) and the 2D one (Program S1), under the initial condition observed from ancient mtDNA data in Syria, namely that 40% of the initial farmer population has haplogroup K, and we compare the results at several distances from the origin. For the 2D model, we choose a coastal node as origin and measure the distances along the coast, rather than with straight lines.

We show the results for several values of η in Fig. S20, where we have measured the fraction of the population with haplogroup K at several locations, 10 generations after the local Neolithic arrival (according to the simulations). From Fig. S20 we can see that the 2D model (lines + symbols) always predicts a lower fraction of population with haplogroup K than the 1D model (lines). However, given that the 1D model is just an approximation, it is interesting to see that the results from both models have similar behaviors and are close enough, so the 1D model is a useful check of the results of the 2D model (especially, the conclusion that very low values of η are necessary in order for the genetic cline to extend across a distance similar to that from Syria to Portugal).

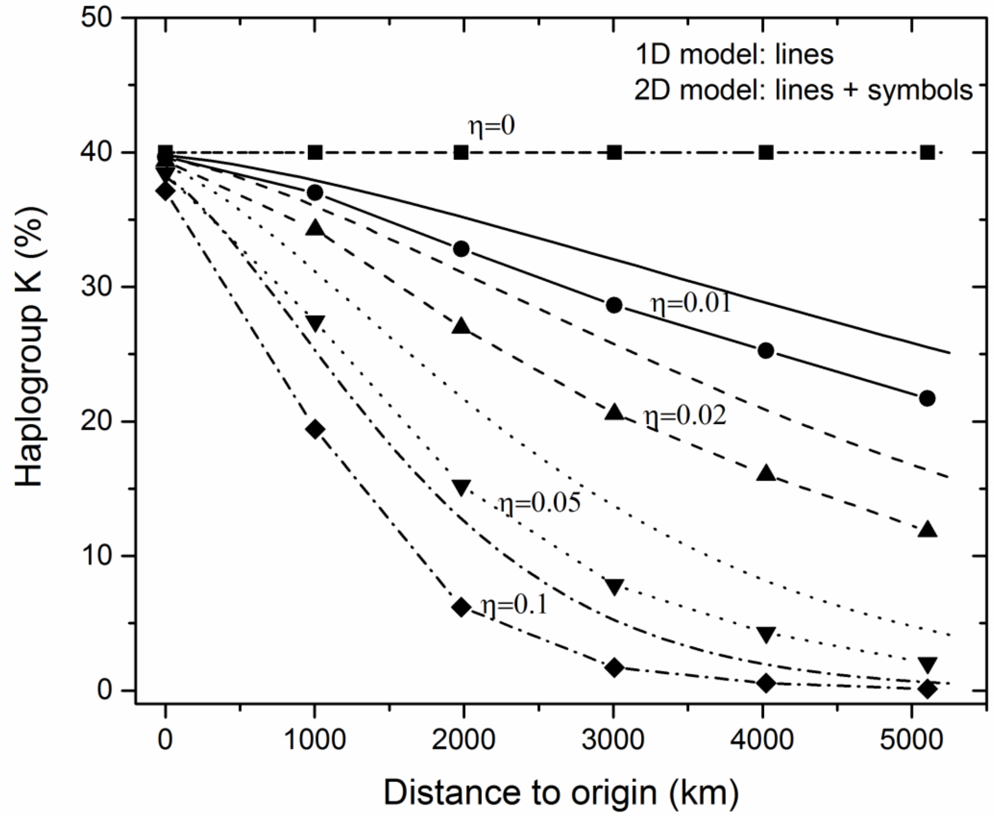

Figure S20. Percentage of mtDNA haplogroup K present in the farmer population that disperses along the coast, as a function of distance to an origin coastal node. Lines correspond to the approximate 1D model developed in Text S12. Lines with symbols correspond to the 2D model on a real map of Europe used in the main paper. All results are measured 10 generations after the local arrival of the Neolithic front (according to the simulations).

.

Text S13. The speed of waves of advance in homogeneous space

In order to perform a check of our simulations we recall that, in two-dimensional homogeneous space (i.e., without seas neither mountains), the speed of the waves of advance of farmers corresponding to our reproduction-dispersal-interbreeding scheme is[57]

$$speed = min_{\lambda>0} \frac{ln\{R_{0,F}(1+\eta)[p_e + (1-p_e)I_0(\lambda r)]\}}{T\lambda}, \tag{S60}$$

where $I_0(\lambda r)$ is the modified Bessel function of the first kind and order zero, r is the average distance that an individual moves per generation, $R_{0,F}$ is the net reproduction rate for farmers, p_e is the persistence, and T is the generation time. Figure 21 shows the results (lines) obtained from equation (S60) when using the same values as in the main paper, Materials and Methods, i.e. $r = 50$ km [52,55], $R_{0,F} = 2.45$ [54], $p_e = 0.38$ [52] and $T = 32$ yr [51]).

We performed additional simulations using, instead of a map of Europe, a homogeneous grid of land nodes (i.e., without sea-travel neither mountain barrier effects), as we did in Fig. S18. However, in order to compare to equation (S60), now we will analyze the spread rate of the front (not the genetic cline as in Fig. S18). We perform our simulations with Program S5 (available at the journal web or at http://copernic.udg.es/QuimFort/2017_08_07r__Program_S5.zip), which performs the same logic as Program S1 (Text S5), but on a homogeneous grid where the Neolithic spreads from its center (Program S5 differs from Program S4, Text S8, in how the initial conditions are set). Initially there are hunter-gatherers (at their saturation density) in all cells but the central one, where there are only farmers (also at their saturation density). Since we are now only interested in the arrival time, the genetic composition of the initial farming population does not affect the results, so we set it at 100%K. In each simulation, a wave of advance of farmers propagates outwards from the center of the grid. In order to determine its speed, for each cell along the x-axis we record the time when the farmer population reaches a population number equal to 10% of its saturation value (however, the wave-of-advance speed is not affected by this percentage, i.e. we would obtain the same speeds by using, e.g., 90%). The speed is then computed as the slope of the linear fit of the distances from the origin versus arrival times. Figure S21 shows the speed of the waves of advance along the horizontal direction (symbols), obtained from those simulations, as a function of the interbreeding parameter η (with $0 \leq \eta \leq 1$, see main paper, Materials and Methods, Cultural transmission). Errors are below 6% (see Data S5), which is reasonable because, in contrast to equation (S60), which assumes a continuous space, simulations are necessarily performed on a grid, i.e., using only a finite number of spatial locations. This confirms the validity of our simulations.

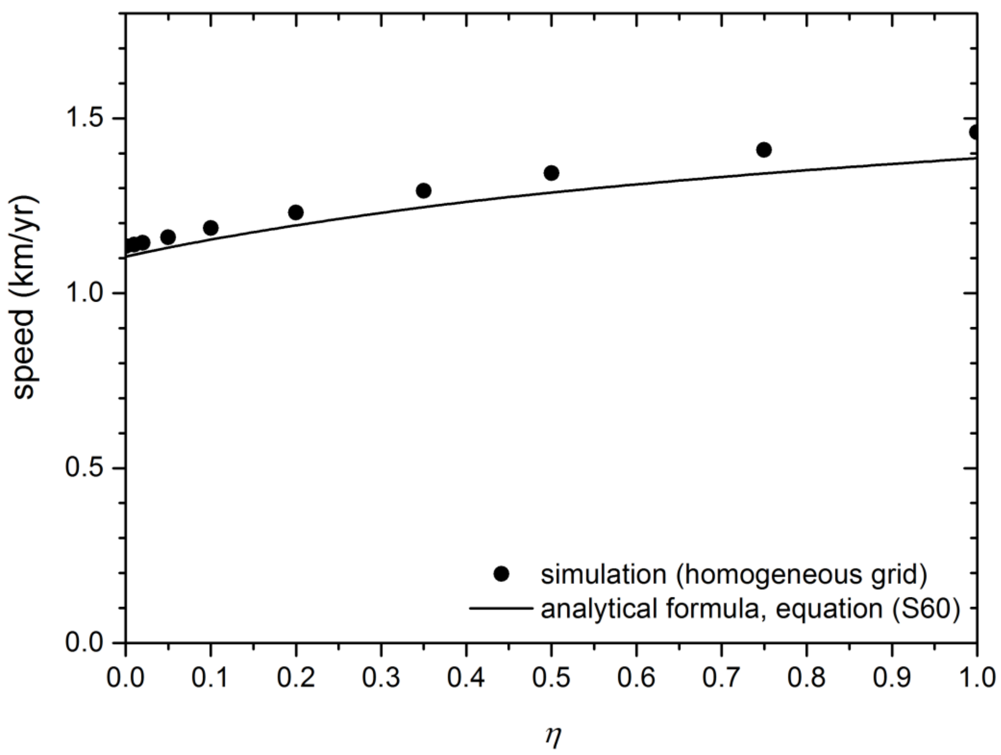

Figure S21. Predicted front speed from the computational model (Program S5) and an analytical approximation on a homogeneous grid. Our simulations on a homogenous grid, i.e. without seas nor mountains (symbols), agree with and the corresponding analytical formula, Eq. (S60) (curve). This is a useful check of our simulations. All results have been obtained using $r = 50$ km [52,55], $R_{0,F} = 2.45$ [54], $p_e = 0.38$ [52] and $T = 32$ yr [51].

Text S14. Pre-Neolithic haplogroups in Neolithic communities

The analysis performed in the main paper indicates, based on the variation of the mitochondrial haplogroup K, that the Neolithic expansion was mostly demic, although with a low contribution of cultural diffusion. Under these circumstances, in addition to the decay in the presence of haplogroup K, we should also be able to observe an increase in the presence of hunter-gatherer haplogroups in the Neolithic communities. Mitochondrial DNA from hunter-gatherers in Central-European was limited to haplogroups U, U4, U5 and U8 [1,35,42,71]. These lineages showed also a high frequency among the western Mediterranean hunter-gatherers[72,73], but the latter also presented important frequencies of haplogroup H lineages[1, 72]; especially haplogroups H1 and H3, which are related to a post glacial expansion from an Iberian refugium[73-75] (in central Europe, on the other hand, H lineages are linked to the spread of the Neolithic[76]).

Figure S22 shows the percentage of hunter-gatherer haplogroups (i.e., haplogroups U, U4, U5 and U8 for regions in the Near East and Eastern and Central Europe, and haplogroups U, U4, U5, U8, H1 and H3, for regions in Iberia and Southern France) with their error bars, for the same regions as in Fig. 2 in the main paper. In Fig. 2, we have fitted a straight line because this is the simplest fit such that it crosses all error bars of the oldest Neolithic cultures (squares and circle). However, this is not possible for Fig. S22, so we fit more appropriate curves, namely a power and an exponential function. This is reasonable since, as we mentioned in the main text, there is no reason why a genetic cline should be linear, and we could actually also fit a decreasing power or exponential curve to Fig. 2. We see that both fits in Fig. S22 show that the percentage of hunter-gatherer lineages present in the Early Neolithic populations increases with distance once the Neolithic front reaches the Central European area (Region '3 Hungary-Croatia Stracevo'), which agrees with our hypothesis that the hunter-gatherer contribution to the Neolithic pool would have increased away from the origin of expansion.

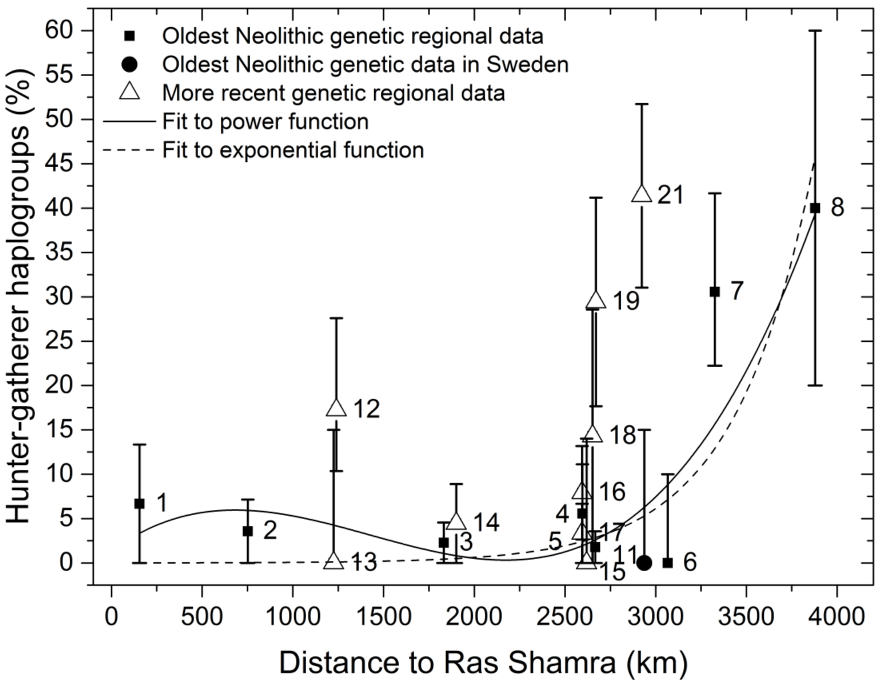

Figure S22. Observed percentage of hunter-gatherer mtDNA haplogroups as a function of the great-circle distance from Ras Shamra (Syria). The haplogroups considered in all regions are U, U4, U8 and U8, while haplogroups H1 and H3 are also included in western Mediterranean regions: 6 North-Eastern Spain Cardial, 7 Spain Navarre, 8 Portugal coastal Early Neolithic and 21 South-Eastern France Treilles. Each number denotes the same cultures as in Fig. 1 (as in Fig. 2, regions with fewer than 8 individuals have been ignored to avoid very large error bars). The solid and dashed lines are regression fits to the 8 oldest regional data (squares) and the oldest data in Sweden (circle). Error bars display 80% confidence-level intervals (see Materials and Methods, Statistical analysis).

In Fig. S22, for distances below 2,000 km the two considered fits show different behaviors, both consistent with the data and their error bars, and both yielding a similar goodness of fit. Therefore, it is not possible to establish which fit is more reasonable. But this does not change our conclusion that the percentage of HG haplogroups increases with distance, as expected. Our results in the main paper attempt to provide an estimation of the average intensity of cultural diffusion at the continental scale, i.e., our purpose is to analyze the overall process, not regional differences. However, it is worth to note that recent studies have suggested that the effect of cultural diffusion increased as farmers spread to further locations[77], which would agree nicely with our observations in Fig. S22.

From Fig. S22 we can also see that, in general, in later periods (triangles) the presence of hunter-gatherer haplogroups increases, since most triangles are located above the lines fitting the data for Early Neolithic populations (black squares and circle). This behavior is consistent with the conclusion in our main paper that after the first arrival, the farmer populations continue incorporating local hunter-gatherer individuals, and therefore the presence of hunter-gatherer haplogroups in the Neolithic populations should increase (as observed in Fig. S22).

We would like to stress that the observed increase at longer distances is not an artificial effect of including H lineages; if we considered only U lineages we would again obtain an increase of hunter-gatherer haplogroups in the early farmer populations, as shown in Fig. S23.

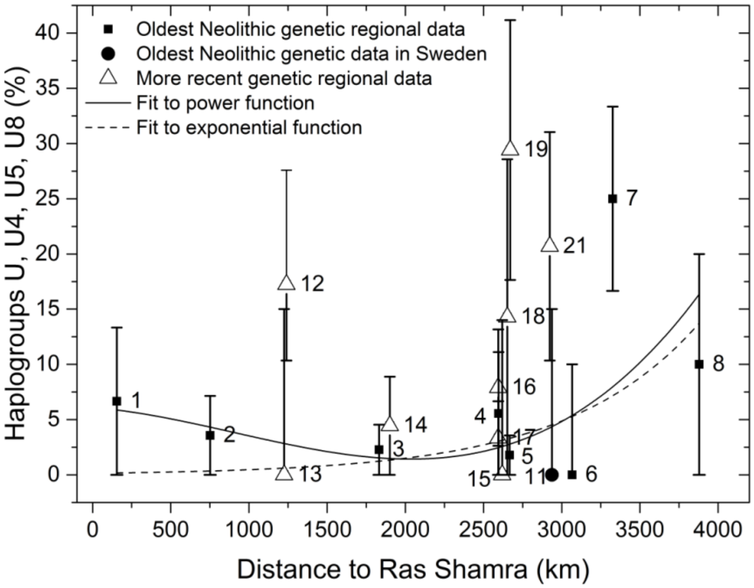

Figure S23. Observed percentage of U haplogroups in Neolithic populations as a function of the great-circle distance from Ras Shamra (Syria). Labels denote the same cultures as in Fig. 1 (as in Fig. 2, regions with fewer than 8 individuals have been ignored to avoid very large error bars). The solid and dashed lines are regression fits to the 8 oldest regional data (squares) and the oldest data in Sweden (circle). Error bars display 80% confidence-level intervals (see Materials and Methods, Statistical analysis).

References

1. Brandt, G. *et al.* Ancient DNA reveals key stages in the formation of central European mitochondrial genetic diversity. *Science* **342**, 257–261 (2013).

2. Excoffier, L., Laval, G. & Schneider, S. Arlequin ver. 3.0: An integrated software package for population genetics data analysis. *Evol. Bioinform. Online* **1**, 47-50 (2005).

3. Tajima, F. Statistical method for testing neutral mutation hypothesis by DNA polymorphism. Genetics **123**, 585-595, (1989).

4. Fu, Y.-X. Statistical tests of neutrality of mutations against population growth, hitchhiking and background selection. Genetics, **147**, 915-925 (1997).

5. Excoffier, L. & Schneider, S.Why hunter-gatherer populations do not show signs of Pleistocene demographic expansions. *Proc. Natl. Acad. Sci.* **96**, 10597-10602 (1999).

6. Haak, W. *et al.* Ancient DNA from European early Neolithic farmers reveals their Near Eastern affinities. *PLoS Biol.* **8**, e1000536 (2010).

7. Pereira, L., Dupanloup, I., Rosser, Z. H., Joblins, M. A. & Barbujani G. Y-Chromosome Mismatch Distributions in Europe. *Mol. Biol. Evol.* **18**, 1259-1271 (2001).

8. Andrews, R. M. *et al.* Reanalysis and revision of the Cambridge reference sequence for human mitochondrial DNA. *Nat. Genet.* **23**, 147 (1999).

9. Nei, M. *Molecular evolutionary genetics*. (Columbia University Press, 1987).

10. Elsner, J., Hofreiter, M., Schibler, J. & Schlumbaum, A. Ancient mtDNA diversity reveals specific population development of wild horses in Switzerland after the Last Glacial Maximum. *PLoS One* **12**, e0177458 (2017).

11. Mathieson, I. *et al.* Genome-wide patterns of selection in 230 ancient Eurasians. *Nature* **528**, 499–503 (2015).

12. Rogers, A. R. & Harpending, H. Population growth makes waves in the distribution of pairwise genetiv differences. *Mol. Biol. Evol.* **9**, 552-569 (1992).

13. Currat, M. & Excoffier, L. The effect of the Neolithic expansion on European molecular diversity. *Proc. R. Soc. B* **272**, 679–688 (2005).

14. Ray, N., Currat, M. & Excoffier, L. Intra-deme molecular diversity in spatially expanding populations. *Mol. Biol. Evol.* **20**, 76-86, (2003).

15. Excofier, L. Patterns of DNA sequence diversity and genetic structure after a range expansion: lessons from the infinite-island model. *Mol. Ecol.* **13**, 853-864 (2004).

16. Ramachandran, S. *et al.* Support from the relationship of genetic and geographic distance in human populations for a serial founder effect originatin in Africa. *Proc. Natl. Acad. Sci.* **102**, 15942-15947 (2005).

17. Slatkin, M. Isolation by distance in equilibrium and non-equilibrium populations. Evolution **47**, 264-279 (1993).

18. Mantel, M. The detection of disease clustering and a generalized regression approach. *Cancer Res.* **27**, 209-220, (1967).

19. Smouse, P. E., Long, J. C. & Sokal, R. R. Multiple regression and correlation extensions of the Mantel Test of matrix correspondence. *Systematic Zoology* **35**, 627-632 (1986).

20. Messina, F. *et al.* Linking between genetic tructure and geographical distance: Study of the maternal gene pool in the Ethiopian population. *Ann. Hum. Biol.* **44**, 53-69 (2017).

21. Legendre, P. & Fortin, M.-J. Comparison of the Mantel test and alternative approaches for detecting complex multivariate relationships in the spatial analysis of genetic data. *Mol. Ecol. Resour.* **10**, 831-844 (2010).

22. Hammer, Ø., Harper, D. A. T. & Ryan, P. D. PAST: Paleontological Statistics Software Package for Education and Data Analysis. *Palaeontol. Electron.* **4**, 1-9 (2001).

23. Slatkin, M. & Hudson, R. R. Pairwise comparisons of mitochondrial DNA sequences in stable and exponentially growing populations. *Genetics* **129**, 555-562 (1991).

24. Bandelt, H.-J., Forster, P. & Röhl, A. Median-joining networks for inferring intraspecific phylogenies. *Mol. Biol. Evol.* **16**, 37-48 (1999).

25. Torroni, A. *et al.* mtDNA analysis reveals a major Late Paleolithic population expansion from southwestern to northwestern Europe. *Am. J. Hum. Genet.* **62**, 1137-1152 (1998).

26. Barbanas, S., Shouche, Y., & Suresh, C. G. High-resolution mtDNA studies of the Indian population: Implications for Palaeolithic settlement of the Indian subcontinent. *Ann. Hum. Genet.* **70**, 42-58 (2006).

27. Drummond, A. J., Rambaut, A., Shapiro, B. & Pybus, O. G. Bayesian coalescent inference of past population dynamics from molecular sequences. *Mol. Biol. Evol.* **22**, 1185-1192 (2005).

28. Bouckaert, R. *et al.* BEAST 2: A Software Platform for Bayesian Evolutionary Analysis. *PLoS Comp. Biol.* **10**, e1003537 (2014).

29. Rambaut, A., Suchard, M. & Drummond, A. J. Tracer v1.6. http://tree.bio.ed.ac.uk/software/tracer/ (2013).

30. Soares, P. *et al*. Correcting for purifying selection: An improved human mitochondrial molecular clock. *Am. J. Hum. Genet.* **84**, 740-759 (2009).

31. Zenger, K., Richardson, B. & Vachot-Griffin, A. A rapid population expansion retains genetic diversity within European rabbits in Australia. *Mol. Ecol.* **12**, 789-794 (2003).

32. Xu, L. *et al.* Variation of Genetic Diversity in a Rapidly Expanding Population of the Greater Long-Tailed Hamster (Tscherskia triton) as Revealed by Microsatellites. *PLoS One* **8**, e54171 (2013).

33. Murphy, S. M. *et al.* Rapid growth and genetic diversity retention in an isolated reintroduced black bear population in the central appalachians *J. Wildlife Manage.* **79**, 807-818, (2015).

34. Coia, G. C. *et al.* Whole mitochondrial DNA sequencing in the Alpine populations and the genetic history of the Neolithic Tyrolean Iceman. *Sci. Rep.* **6**, 18932 (2015).

35. Bramanti, B. *et al.* Genetic discontinuity between local hunter-gatherers and central Europe's first farmers. *Science* **326**, 137–140 (2009).

36. Malmström, H. *et al.* Ancient DNA Reveals Lack of Continuity between Neolithic Hunter-Gatherers and Contemporary Scandinavians. *Curr. Biol.* **19**, 1758-1762 (2009).

37. Malmström, H. *et al.* Ancient mitochondrial DNA from the northern fringe of the Neolithic farming expansion in Europe sheds light on the dispersion process. *Phil. Trans. R. Soc. B* **370**, 20130373 (2015).

38. Eriksson, G. *et al.* Same island, different diet: cultural evolution of food practice on Öland, Sweden, from the Mesolithic to the Roman Period. *J Anthropol Archaeol* 27:520-543 (2008).

39. Malmer, M. P. *The Neolithic of south Sweden: TRB, GRK, and STR* (Royal Swedish Academy of Letters History and Antiquities. Almquist & Wiksell International, 2002).

40. Skoglund, P. *et al.* Genomic diversity and admixture differs for stone-age Scandinavian foragers and farmers. *Science* **344**, 747-750 (2014).

41. Hofmanová, Z. *et al.* Early farmers from across Europe directly descended from Neolithic Aegeans. *Proc. Natl. Acad. Sci.* **113**, 6886–6891 (2016).

42. Fu, Q. *et al.* The genetic history of Ice Age Europe. *Nature* **534**, 200–205 (2016).

43. Olalde, I. *et al.* A common genetic origin for early farmers from Mediterranean Cardial and Central European LBK cultures. *Mol. Biol. Evol.* **32**, 3132-3142 (2015).

44. Lazaridis, I. *et al.* Genomic insights into the origin of farming in the ancient Near East. *Nature* **536**, 419–424 (2016).

45. Fernández, E. *et al.* Ancient DNA analysis of 8,000 B.C. Near Eastern farmers supports an early Neolithic pioneer maritime colonization of mainland Europe through Cyprus and the Aegean Islands. *PLoS Genet.* **10**, e1004401 (2014).

46. Fort, J., Pujol, T. & vander Linden, M. Modelling the Neolithic transition in the Near East and Europe. *Am. Antiq.* **77**, 203–220 (2012).

47. Legendre, P. & Legendre, L. *Numerical Ecology*. (Elsevier, 2012).

48. Messina, F. *et al.* Spatially explicit models to investigate geographic patterns in the distribution of forensic STRs: Application to the North-Eastern Mediterranean. *PLoS One* **11**, e0167065 (2016).

49. Rosenberg, M. S. & Anderson, C. D. PASSaGE: Pattern Analysis, Spatial Statistics and Geographic Exegesis. Version 2. *Meth. Ecol. Evol.* **2**, 229-232 (2011).

50. Olden, N. L. Assessing the significance of a spatial correlogram. *Geogr. Anal.* **16**, 1-16 (1984).

51. Fort, J., Jana, D. & Humet, J. M. Multidelayed random walks: Theory and application to the Neolithic transition in Europe. *Phys. Rev. E* **70**, 031913 (2004).

52. Fort, J., Pérez-Losada, J. & Isern, N. Fronts from integrodifference equations and persistence effects on the Neolithic transition. *Phys. Rev. E* **76**, 031913 (2007).

53. Fort, J. Synthesis between demic and cultural diffusion in the Neolithic transition in Europe. *Proc. Natl. Acad. Sci.* **109**, 18669–18673 (2012).

54. Isern, N., Fort, J. & Pérez-Losada, J. Realistic dispersion kernels applied to cohabitation reaction-dispersion equations. *J. Stat. Mech. Theor. Exp.* **2008**, P10012 (2008).

55. Ammerman, A. J. & Cavalli-Sforza, L. L. *The Neolithic transition and the genetics of populations of Europe* (Princeton University Press, 1984).

56. Cronk, L. From hunters to herders: Subsistence change as a reproductive strategy among the Mukogodo. *Curr. Anthropol.* **30**, 224–234 (1989).

57. Fort, J. Vertical cultural transmission effects on demic front propagation: Theory and application to the Neolithic transition in Europe. *Phys. Rev. E* **83**, 056124 (2011).

58. Early, J. D. & Headland, T. N. *Population dynamics of a Philippine rain forest people: The San Ildefonso Agta* (University of Florida Press, 1998).

59. Cavalli-Sforza, L. L. & Feldman, M. W. *Cultural transmission and evolution: A quantitative approach* (Princeton University Press, 1981).

60. Murray, J.D. *Mathematical Biology*, Vol 2 (Springer-Verlag, 2001).

61. Zilhão, J. Radiocarbon evidence for maritime pioneer colonization at the origins of farming in west Mediterranean Europe. *Proc. Natl. Acad. Sci.* **98**, 14180-14185 (2001).

62. Isern, N., Zilhão, J., Fort, J. & Ammerman, A. J. Modelling the role of voyaging in the coastal spread of the early Neolithic in the West Mediterranean. *Proc. Natl. Acad. Sci.* **114**, 897-902 (2017).

63. Fort, J. & Méndez, V. Time-delayed theory of the Neolithic transition in Europe. *Phys. Rev. Lett.* **82**, 867-870 (1999).

64. Isern, N., Fort, J., Carvalho, A. F., Gibaja, J. F. & Ibañez, J. J. The Neolithic transition in the Iberian Peninsula: data analysis and modelling. *J. Archaeol. Method. Theory.* **21**, 447-460 (2014).

65. Wobst, M. Boundary conditions for Paleolithic social systems: a simulation approach. *Am. Antiq.* **39**, 147-178 (1974).

66. Fort, J. Demic and cultural diffusion propagated the Neolithic transition across different regions of Europe. *J. Roy. Soc. Interface* **12**, 20150166 (2015).

67. Semino, O. *et al.* The genetic legacy of Paleolithic Homo sapiens sapiens in extant Europeans: a Y chromosome perspective. *Science* **290**, 1155-1159 (2000).

68. Raviv, Y. & Intrator, N. Bootstrapping with Noise: An Effective Regularization Technique. *Connection Science* **8**, 355-372 (1996).

69. Efron, B. *The jackknife, the bootstrap and other resampling plans* (Society of Industrial and Applied Mathematics, 1982).

70. Dixon, P. M. The bootstrap and the jackknife: describing the precision of ecological indexes in *Design and analysis of ecological experiments* (eds. Scheiner, S.M. & Gurevitch, J.) 290-318 (Chapman and Hall, 1993).

71. Fu, Q. *et al.* A revised timescale for human evolution based on ancient mitochondrial genomes. *Curr. Biol.* **23**, 553-559 (2013).

72. Hervella, M. *et al.* Ancient DNA from hunter-gatherer and farmers groups from Northern Spain supports a random dispersion model for the Neolithic expansion into Europe. *PLoS One* **7**, e34417 (2012).

73. Lacan, M. Ancient DNA reveals male diffusion through the Neolithic Mediterranean route. *Proc. Natl. Acad. Sci.* **108**, 9788-9791 (2011).

74. Gamba, C. *el al.* Ancient DNA from an Early Neolithic Iberian population supports a pioneer colonization by first farmers. *Mol. Ecol.* **21**, 45-56 (2012).

75. Brotherton, P. *et al*. Neolithic mitochondrial haplogroup H genomes and the genetic origins of Europeans. *Nat. Comm.* **4**, 1764 (2013).

76. Fu, Q., Rudan, P., Pääbo, S. & Krause J. Complete Mitochondrial Genomes Reveal Neolithic Expansion into Europe. *PLoS One* **7**, e32473 (2012).

77. Rivollat, M. *et al.* Investigating mitochondrial DNA relationships in Neolithic Western Europe through serial coalescent simulations. *Eur. J. Hum. Gen.* **25**, 388-392 (2017).
